# Inhibition of fatty acid amide hydrolase prevents pathology in neurovisceral acid sphingomyelinase deficiency by rescuing defective endocannabinoid signaling

**DOI:** 10.15252/emmm.201911776

**Published:** 2020-10-05

**Authors:** Adrián Bartoll, Ana Toledano‐Zaragoza, Josefina Casas, Manuel Guzmán, Edward H Schuchman, María Dolores Ledesma

**Affiliations:** ^1^ Centro Biologia Molecular Severo Ochoa (CSIC‐UAM) Madrid Spain; ^2^ RUBAM IQAC‐CSIC & CIBEREHD Barcelona Spain; ^3^ Department of Biochemistry and Molecular Biology Centro de Investigación Biomédica en Red sobre Enfermedades Neurodegenerativas (CIBERNED) Instituto Ramón y Cajal de Investigación Sanitaria (IRYCIS) Instituto Universitario de Investigación Neuroquímica (IUIN) Complutense University Madrid Spain; ^4^ Department of Genetics & Genomic Sciences Icahn School of Medicine at Mount Sinai New York NY USA

**Keywords:** endocannabinoids, neurodegeneration, Niemann–Pick, sphingomyelin, Genetics, Gene Therapy & Genetic Disease, Neuroscience, Chemical Biology

## Abstract

Acid sphingomyelinase deficiency (ASMD) leads to cellular accumulation of sphingomyelin (SM), neurodegeneration, and early death. Here, we describe the downregulation of the endocannabinoid (eCB) system in neurons of ASM knockout (ASM‐KO) mice and a ASMD patient. High SM reduced expression of the eCB receptor CB_1_ in neuronal processes and induced its accumulation in lysosomes. Activation of CB_1_ receptor signaling, through inhibition of the eCB‐degrading enzyme fatty acid amide hydrolase (FAAH), reduced SM levels in ASM‐KO neurons. Oral treatment of ASM‐KO mice with a FAAH inhibitor prevented SM buildup; alleviated inflammation, neurodegeneration, and behavioral alterations; and extended lifespan. This treatment showed benefits even after a single administration at advanced disease stages. We also found CB_1_ receptor downregulation in neurons of a mouse model and a patient of another sphingolipid storage disorder, Niemann–Pick disease type C (NPC). We showed the efficacy of FAAH inhibition to reduce SM and cholesterol levels in NPC patient‐derived cells and in the brain of a NPC mouse model. Our findings reveal a pathophysiological crosstalk between neuronal SM and the eCB system and offer a new treatment for ASMD and other sphingolipidoses.

## Graphical abstract

This study shows the downregulation of the endocannabinoid (eCB) system in neurons of two lysosomal storage disorders: acid sphingomyelinase deficiency (ASMD) and Niemann Pick type C (NPC). Brain pathology in mouse models of these diseases was prevented by eCB pharmacological enhancement.

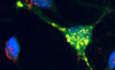

**The paper explained**

**Problem**

Acid sphingomyelinase deficiency (ASMD) and Niemann–Pick type C (NPC) are fatal lysosomal storage disorders in which lipids like sphingomyelin accumulate in cells leading to severe neurological involvement and early death.

**Results**

Here, we describe the sphingomyelin‐induced downregulation of the eCB receptor CB_1_ in neurons of mouse models and patients of these diseases. Enhancement of the eCB system by oral treatment with inhibitors of the eCB‐degrading enzyme fatty acid amide hydrolase (FAAH), reduced lipid storage, prevented neurodegeneration, and increased life span in the mouse models.

**Impact**

These results discover a novel therapeutic target and a non‐invasive strategy for ASMD and NPC with potential for the treatment of other sphingolipid storage disorders.

## Introduction

The endocannabinoid (eCB) system consists of a family of modulatory lipid messengers together with their specific receptors and metabolic enzymes. This cell signaling system regulates many aspects of neuronal development and function (Maccarrone *et al*, 2014) and influences synaptic communication in a plethora of physiopathological events, including cognitive and emotional processes (Katona & Freund, 2012; Mechoulam & Parker, 2013). Both anandamide (*N*‐arachidonoylethanolamine, AEA) and 2‐arachidonoylglycerol (2‐AG), the major eCBs, bind to the G protein‐coupled receptors CB_1_ and CB_2_, of which CB_1_ is by large the most abundant in the brain (Pertwee *et al*, 2010). Concertedly, the enzymes fatty acid amide hydrolase (FAAH) and monoacylglycerol lipase (MAGL) contribute to the bulk of AEA and 2‐AG degradation, respectively, and are essential for maintaining appropriate levels of both molecules (Di Marzo, 2009; Shin *et al*, 2019).

The neuromodulatory actions of eCBs are believed to rely on their release from a postsynaptic neuron upon stimulation and their subsequent retrograde action on presynaptic terminals, where they activate CB receptors, thus modulating plasma membrane ion conductivity and depressing neurotransmitter release (Castillo *et al*, 2012). CB_1_ receptor activation has been long related to inhibitory and excitatory synaptic plasticity underlying learning and memory (Sullivan, 2000; Domenici *et al*, 2006; Kawamura *et al*, 2006; Takahashi & Castillo, 2006). Alterations in the brain eCB system have been linked to various psychiatric conditions. For example, impaired AEA degradation underlies attention deficits and hyperactivity disorder (Centonze *et al*, 2009). It has also been proposed that endogenous CB_1_ receptor activation serves as a buffer against depression, while its downregulation results in depressive symptoms (Mangieri & Piomelli, 2007; Patel & Hillard, 2009; Fowler, 2015). In addition, the eCB system plays important prohomeostatic, anti‐inflammatory, and neuroprotective functions (Sarne & Mechoulam, 2005; van der Stelt & Di Marzo, 2005; Chiarlone *et al*, 2014; Gomez‐Galvez *et al*, 2016), and its possible involvement in different neurodegenerative diseases (van der Stelt *et al*, 2005; Blazquez *et al*, 2011; Aymerich *et al*, 2018) has stimulated the therapeutic interest of eCB modulation for the treatment of neurological disorders.

A series of previous studies point to the membrane environment as a critical factor for the regulation of the eCB system. For example, acute cholesterol depletion increases CB_1_‐dependent signaling in neuronal cells (Bari *et al*, 2005a,b), and lipid raft‐dependent and non‐dependent endocytosis might be differentially involved in the transport of AEA and the axonal sorting of CB_1_ (McFarland & Barker, 2004; Leterrier *et al*, 2006; Fletcher‐Jones *et al*, 2020). Moreover, eCBs are believed to bind to and activate CB_1_ receptors by lateral diffusion within the lipid bilayer, rather than reaching the binding site from the extracellular surface (Lynch & Reggio, 2006; Hua *et al*, 2017). These observations highlight the relevance of membrane lipids to eCB function and the need to understand in further detail their contribution.

The infantile, neurovisceral form of acid sphingomyelinase deficiency (ASMD also known as acute neuronopathic ASMD or Niemann–Pick type A) is a fatal lipidosis that leads to hepatosplenomegaly, pulmonary involvement, impaired psychomotor development, and neurodegeneration (McGovern *et al*, 2017). Disease‐causing mutations in the gene encoding ASM (*SMPD1*), a key enzyme responsible for SM degradation, induce the accumulation of this lipid in all body cells (Schuchman & Desnick, 2017). In neurons from ASM knockout (ASM‐KO) mice, high SM levels in the lysosomes and the synaptic and plasma membrane lead to lysosomal permeabilization and autophagy impairment (Gabande‐Rodriguez *et al*, 2014), unpolarized distribution of proteins (Galvan *et al*, 2008), dendritic spine anomalies (Arroyo *et al*, 2014), and Ca^2+^ imbalance (Perez‐Canamas *et al*, 2016). Enzyme replacement therapy (ERT) by intravenous infusion of recombinant human ASM efficiently treats the non‐neurological pathology in ASM‐KO mice (Miranda *et al*, 2000) and in ASMD patients (Wasserstein *et al*, 2015). Two clinical trials (ASCEND and ASCEND‐Peds) are currently ongoing (NCT02004691 and NCT02292654, respectively) for ASMD patients that lack neurological involvement (i.e., Niemann–Pick type B or chronic visceral ASMD) (McGovern *et al*, 2017). However, the inability of the recombinant enzyme to cross the blood–brain barrier and the absence of improvement in the neurological component after ERT in ASM‐KO mice (Miranda *et al*, 2000) indicate that this approach would be inadequate for the neurovisceral forms.

In addition to ASM, there are other sphingomyelinases that are active in the mammalian brain and can degrade SM. Indeed, pharmacological enhancement of the neutral sphingomyelinase (NSM) by the glucocorticoid dexamethasone reduced SM levels in synapses of ASM‐KO mice and improved their memory and learning capabilities (Arroyo *et al*, 2014). These findings pointed to the activation of NSM, an enzyme that is not genetically affected in ASMD, as a suitable strategy to diminish high brain SM levels in the disease. It has been shown that cannabinoid‐evoked stimulation of CB_1_ receptors activates NSM through the adaptor protein FAN, thus reducing SM levels in cultured rat astrocytes (Sanchez *et al*, 2001). Early work in cultured fibroblasts from a severe ASMD patient also showed the capacity of cannabidiol to reduce SM levels (Burstein *et al*, 1984). We thus hypothesized that enhancing the activity of the eCB system in brain and peripheral organs could be therapeutically beneficial for patients with both the neurovisceral and visceral forms of ASMD. In this study, we provide proof of concept for this hypothesis by showing that (i) elevated SM leads to reduced CB_1_ expression and altered localization in neurons, (ii) there is a pathological downregulation of the eCB system in neurons of ASM‐KO mice and of an infantile ASMD patient, and (iii) inhibitors of the eCB hydrolytic enzyme FAAH exhibit safety and efficacy to treat brain and peripheral pathology in ASM‐KO mice. We also extend this concept to another sphingolipid storage disease, NPC, by showing CB_1_ receptor downregulation in a mouse model and a patient and that FAAH inhibition reduces SM and cholesterol levels in NPC patient‐derived cells and in the brain of a NPC mouse model.

## Results

### Characterization of CB_1_ receptor anomalies in the brains of ASM‐KO mice and a infantile neurovisceral ASMD patient

**Figure 1:**
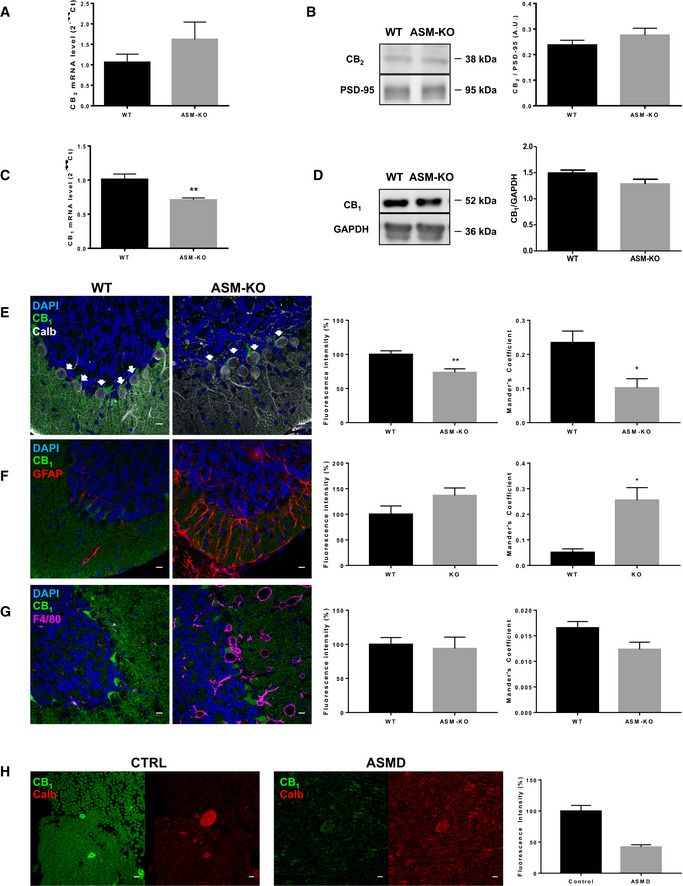
eCB alterations in the cerebellum of ASM‐KO mice and an infantile, neurovisceral ASMD patient AMean ± SEM CB_2_ mRNA levels in cerebellar extracts of WT and ASM‐KO mice (*n *=* *5 mice per group).BWestern blot against CB_2_ and PSD‐95 (used as loading control) and graph showing mean ± SEM CB_2_ protein levels in cerebellar extracts of WT and ASM‐KO mice (*n *=* *6 mice per group).CMean ± SEM CB_1_ mRNA levels in cerebellar extracts of WT and ASM‐KO mice (**P =* *0.0013, *n *=* *6 mice per group, Student's *t*‐test).DWestern blot against CB_1_ and GAPDH (used as loading control) and graph showing mean ± SEM CB_1_ protein levels in cerebellar extracts of WT and ASM‐KO mice (*n *=* *3 mice per group).E–GImmunofluorescence images against CB_1_ and the following cellular markers: calbindin for Purkinje cells (E), GFAP for astrocytes (F), and F4/80 for microglia (G) in the cerebellum of WT and ASM‐KO mice. DAPI stains cell nuclei. Graphs to the left show mean ± SEM intensity associated with CB_1_ in the Purkinje cells (shown by white arrows, E), astrocytes (F), and microglia (G) expressed as percentage of the values obtained in WT mice. Graphs to the right show the Mander's coefficient that indicates degree of co‐localization between CB1 and the cellular markers for Purkinje cells (E), astrocytes (F), and microglia (G) (E: **P_Fluorecence_ _intensity_ = 0.0082, *P_Mander's Coefficient_ = 0.0158; F: *P_Mander's Coefficient_ = 0.0115; *n *=* *5 mice per group, Student's *t*‐test). Scale bar, 100 μm.HImmunofluorescence images against CB_1_ and the Purkinje cell marker calbindin in the cerebellum of age‐matched control and ASMD‐affected children. Graph shows mean ± SEM intensity associated with CB_1_ in the Purkinje cells expressed as percentage of the values obtained in the control child (16 and 15 replicates in control and ASMD, respectively). Scale bar, 10 μm. Source data are available online for this figure.

CB_1_ and CB_2_ receptors mediate eCB signaling in the mammalian brain. To determine whether these receptors are altered in ASM‐KO mice, we measured their mRNA and protein levels in the brains of age‐matched, 4‐month‐old WT and ASM‐KO mice. We focused on the analysis of the cerebellum, which is the most affected brain area in the disease. Real‐time quantitative PCR (RT‐qPCR) showed no significant differences in the levels of CB_2_ mRNA (Fig 1A). Protein levels of this receptor were also unchanged as determined by Western blot in cerebellar extracts of ASM‐KO mice compared to WT littermates (Fig 1B). In contrast, mRNA levels of CB_1_ were 30% diminished in the cerebellum (Fig 1C). Similar reductions (40 and 30%, respectively) were also found in other brain areas such as the hippocampus and prefrontal cortex (Appendix Fig S1A). Western blot of cerebellar extracts showed a slight 13% reduction in CB_1_ protein levels that was not statistically significant (Fig 1D). Similar results were found by Western blot of hippocampal and cortical extracts (Appendix Fig S1B). Slight decreases in CB_1_ protein levels were detected by immunofluorescence in these two brain areas (Appendix Fig S1C). To monitor the cell type specificity of CB_1_ protein expression, we performed co‐labeling with cellular markers by immunofluorescence in the cerebellum. This analysis indicated a significant 26% reduction in the levels of CB_1_ protein in the Purkinje neurons, identified by calbindin staining, and a reduced co‐localization between CB_1_ and these cells as indicated by the Mander's coefficient, which reflects the amount of CB_1_ in this particular cell population with respect to the total CB_1_ staining in the tissue (Fig 1E). CB_1_‐associated intensity was not significantly changed in astrocytes, identified by GFAP staining. However, the Mander's coefficient between CB_1_ and astrocytes increased, probably due to the elevated number of these cells in the ASM‐KO compared to WT cerebellum (Fig 1F and Appendix Fig S2A). CB_1_ levels and co‐localization did not change in microglia, identified by F4/80 staining, which also increased their number in the ASM‐KO cerebellum (Fig 1G and Appendix Fig S2B). These changes in the balance among cell populations in the ASM‐KO mouse brains might prevent the detection of the neuronal‐specific CB_1_ reduction in the biochemical experiments. To determine whether the reduction in CB_1_ protein levels observed in the Purkinje neurons of the ASM‐KO mouse also occurred in patients, we gained access to fixed tissue from the cerebellum of a 3‐year‐old child affected by infantile neurovisceral ASMD and compared CB_1_ levels in the calbindin‐positive cells by immunofluorescence with an age‐matched non‐affected child. CB_1_ protein was notably reduced in the Purkinje cells of the ASMD patient (Fig 1H). Reduction of CB_1_ levels was also observed in neurons (identified by MAP2 staining) of the medium bulb, the other brain area to which we had access in the ASMD patient (Appendix Fig S3). While these observations are not conclusive, since they were made in a single patient, they suggest common CB_1_ alterations in ASMD‐affected humans and mice.

### High SM induces CB_1_ receptor reduction and misdistribution in ASM‐KO neurons

**Figure 2:**
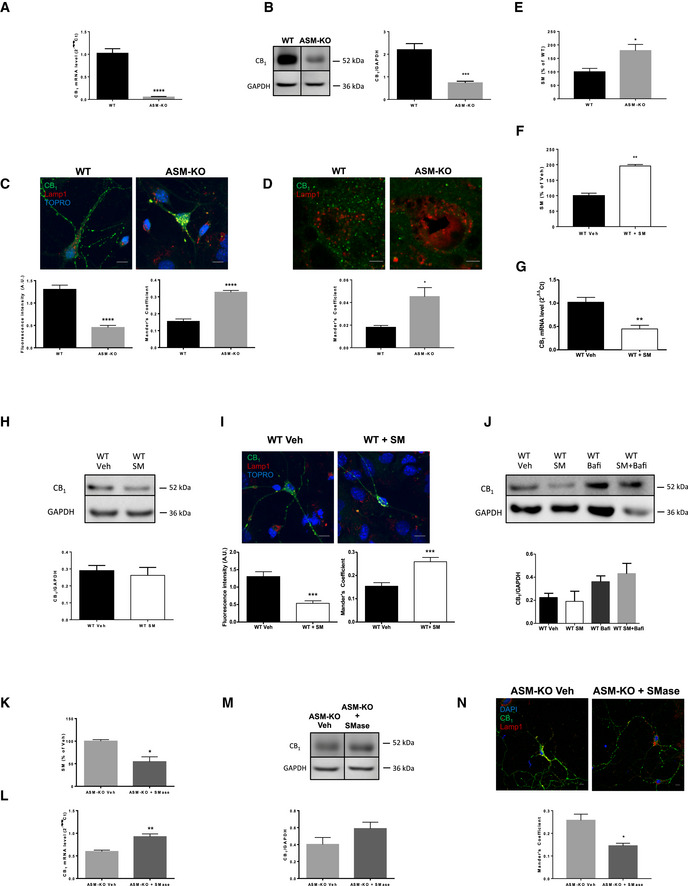
CB_1_ alterations in ASM‐KO neurons are due to high SM levels AMean ± SEM CB_1_ mRNA levels in cultured hippocampal neurons from WT and ASM‐KO mice (****P <* *0.0001, *n *=* *5 independent cultures, Student's *t*‐test).BWestern blot against CB_1_ and GAPDH (used as loading control). Images belong to the same blot but to non‐consecutive lanes as indicated by the boxes. Graph showing mean ± SEM CB_1_ protein levels in cultured neurons from WT and ASM‐KO mice (***P =* *0.0009, *n *=* *5 independent cultures, Student's *t*‐test).CImmunofluorescence images against CB_1_ and the lysosomal marker Lamp1 in cultured neurons from WT and ASM‐KO mice. TOPRO stains cell nuclei. Graphs show mean ± SEM intensity associated with CB_1_ in the neuronal processes expressed in arbitrary units (left) or the Mander's coefficient for the co‐localization of CB_1_ with Lamp1 (right; ****P <* *0.0001, *n *=* *3 independent cultures, > 30 neurons per culture, Student's *t*‐test). Scale bar, 10 μm.DImmunofluorescence images against CB_1_ and the lysosomal marker Lamp1 in Purkinje cells of the cerebellum from WT and ASM‐KO mice. Graph shows mean ± SEM Mander's coefficient indicative of the degree of co‐localization of CB_1_ and Lamp1 (*P =* *0.0213, *n* = 4 mice per group, Student's *t*‐test). Scale bar, 5 μm.E, FGraphs show mean ± SEM SM levels, expressed as percentage of WT values, in extracts containing the same amount of protein of cultured neurons from WT and ASM‐KO mice (E) or from WT mice incubated or not with 40μΜ SM (F). (E: *P =* *0.0405; F: **P =* *0.0100, *n *=* *2 independent cultures, Student's *t*‐test).GMean ± SEM CB_1_ mRNA levels in cultured neurons from WT mice incubated or not with 40 μM SM (**P =* *0.0021, *n *=* *5 independent cultures, Student's *t*‐test).HWestern blot against CB_1_ and GAPDH (used as loading control) and graph showing mean ± SEM CB_1_ protein levels in cultured neurons from WT mice incubated or not with 40 μΜ SM (*n *=* *2 independent cultures, Student's *t*‐test)IImmunofluorescence images against CB_1_ and Lamp1 in cultured neurons from WT mice incubated or not with 40 μΜ SM. Graphs show mean ± SEM intensity associated with CB_1_ in the neuronal processes expressed in arbitrary units (left) or the Mander's coefficient for the co‐localization of CB_1_ with Lamp1 (right; ***P <* *0.0001, *n *=* *3 independent cultures, > 30 neurons per culture, Student's *t*‐test). Scale bar, 10 μm.JWestern blot against CB_1_ and GAPDH (used as loading control) and graph showing mean ± SEM CB_1_ protein levels in cultured neurons from WT mice treated with vehicle (veh), with 40 μM SM (SM), with 0.1 μM bafilomycin (BafA1), or with 40 μM SM and 0.1 μM bafilomycin (SM + BafA1). Graph shows mean ± SEM CB_1_ levels normalized to GAPDH in arbitrary units (*n *=* *2 independent cultures, one‐way ANOVA).KMean ± SEM SM levels in cultured neurons from ASM‐KO mice incubated or not with exogenous SMase (*P =* *0.0157, *n *=* *3 independent cultures, Student's *t*‐test).LMean ± SEM CB_1_ mRNA levels in cultured neurons from ASM‐KO mice incubated or not with exogenous SMase (**P =* *0.0015, *n *=* *5 independent cultures, Student's *t*‐test).MWestern blot against CB_1_ and GAPDH (used as loading control). Images belong to the same blot but to non‐consecutive lanes as indicated by the boxes. Graph showing mean ± SEM CB_1_ protein levels normalized to GAPDH in extracts from cultured neurons from ASM‐KO mice incubated or not with exogenous SMase (*n *=* *5 independent cultures, Student's *t*‐test).NImmunofluorescence images against CB_1_ and the lysosomal marker Lamp1 in cultured neurons from ASM‐KO mice incubated or not with exogenous SMase. DAPI stains cell nuclei. Graph shows mean ± SEM Mander's coefficient for the co‐localization of CB_1_ with Lamp1 (*P =* *0.0189, *n *=* *3 independent cultures, > 30 neurons per culture, Student's *t*‐test). Scale bar, 10 μm. Source data are available online for this figure.

To further understand the impact of ASM deficiency on CB_1_ expression in neurons, the cell type in which we observed alterations in the tissue analysis (Fig 1), we analyzed CB_1_ levels and distribution in cultured primary neurons from WT and ASM‐KO mice. CB_1_ mRNA levels were drastically reduced (95%) in the ASM‐KO hippocampal neurons (Fig 2A). This was accompanied by a significant 66% reduction of CB_1_ protein levels measured by Western blot of neuronal extracts (Fig 2B). The immunofluorescence analysis also indicated a change in the distribution of CB_1_. Thus, while the receptor was preferentially lost from the neuronal processes (65% reduction), its localization in the lysosomal compartment (as visualized by the lysosomal marker Lamp1) increased in the ASM‐KO neurons (Mander's coefficient 0.15 and 0.33 in WT and ASM‐KO neurons, respectively) (Fig 2C). A higher co‐localization of CB_1_ with lysosomes was also observed *in vivo* in the Purkinje cells of the cerebellum of ASM‐KO compared to WT mice (Mander's coefficient 0.019 and 0.042 in WT and ASM‐KO mice, respectively [Fig 2D]).

Sphingomyelin accumulation is a key pathological hallmark in ASM‐KO neurons, which we confirmed in our hippocampal neuronal cultures (Fig 2E). To determine whether this was responsible for the aforementioned alterations in CB_1_ expression, cultured WT neurons were incubated with 40 μM SM for 48 h. These conditions increased SM to levels similar to those found in ASM‐KO neurons (Fig 2F). SM addition produced a 60% decrease in CB_1_ mRNA, as measured by RT‐qPCR (Fig 2G). CB_1_ protein levels were reduced by 10% as determined by Western blot, although this value was not statistically significant (Fig 2H). Immunofluorescence analysis in the cultured WT neurons incubated with SM showed CB_1_ protein reduction from the neuronal processes (by 58%) and increased localization of the receptor in the lysosomes (Mander's coefficient raised from 0.15 to 0.26) (Fig 2I). To determine whether increased co‐localization of CB_1_ in lysosomes could be responsible for its overall low levels due to increased degradation, we measured the amount of the protein in WT neurons incubated or not with 40 μM SM for 48 h in the presence or absence of the lysosomal inhibitor bafilomycin. This drug prevented by 61% the SM‐induced CB_1_ reduction (Fig 2J). In a set of complementary experiments to those of SM addition to WT neurons, we diminished SM levels in cultured ASM‐KO neurons by exogenous addition of sphingomyelinase. This treatment, which lowered SM levels by 46% (Fig 2K), resulted in a 55% increase in CB_1_ mRNA (Fig 2L). CB_1_ protein levels also increased by 45%, although this value was not statistically significant (Fig 2M). Immunofluorescence analyses showed the decreased co‐localization of CB_1_ and Lamp1 upon sphingomyelinase treatment in the ASM‐KO neurons (Mander's coefficient 0.26 in non‐treated cells and 0.15 in sphingomyelinase‐treated cells; Fig 2N). This treatment induced reductions on the SM amount and CB_1_ mRNA levels, although not statistically significant, and no evident effect on CB_1_ protein levels in WT neurons (Appendix Fig S4).

Altogether, these data show that reduced expression of CB_1_ in ASM‐KO neurons correlated with elevated SM and increased delivery of CB_1_ to lysosomes, leading to its degradation. CB_1_ expression and localization could be recovered in ASM‐KO neurons by reducing SM levels.

### eCB signaling reduces SM levels in cultured ASM‐KO neurons

**Figure 3:**
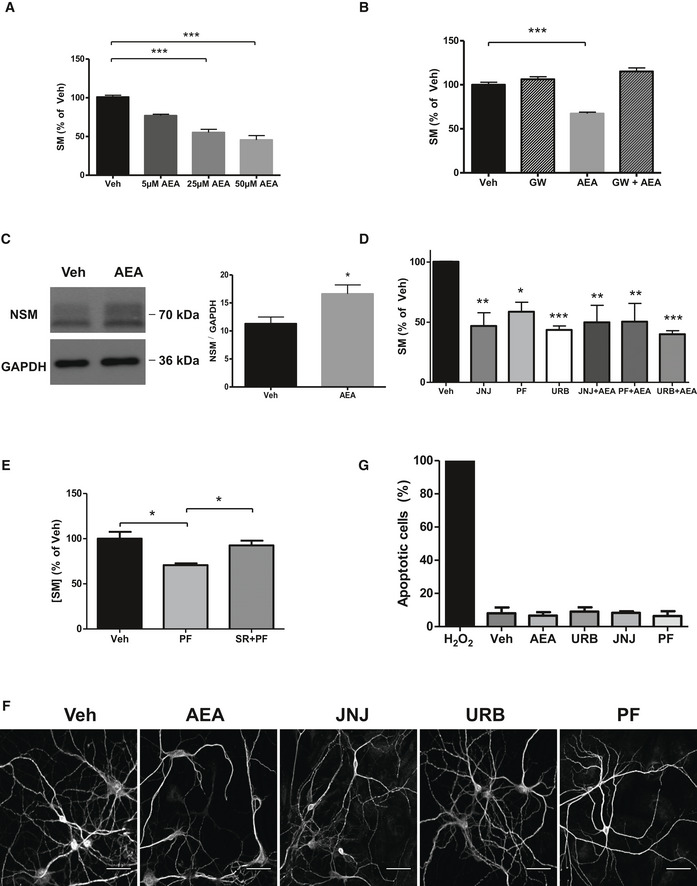
eCB enhancement reduces SM levels in cultured ASM‐KO neurons AMean ± SEM SM levels, expressed as percentage of vehicle values, in cultured neurons from ASM‐KO mice treated with vehicle or with the indicated concentrations of AEA (***P <* *0.0001, *n *=* *3 independent cultures, one‐way ANOVA, Bonferroni *post hoc*).BMean ± SEM SM levels, expressed as percentage of vehicle values, in cultured neurons from ASM‐KO mice treated with vehicle, the inhibitor of NSM GW, AEA, or the combination of GW and AEA (***P <* *0.0001, *n *=* *3 independent cultures, one‐way ANOVA, Bonferroni *post hoc*).CWestern blot against NSM and GAPDH (used as loading control) and graph showing mean ± SEM NSM protein levels in extracts from cultured neurons treated with vehicle or with 50 μM AEA (*P =* *0.0230, *n *=* *6 independent cultures, Student's *t*‐test).DMean ± SEM SM levels, expressed as percentage of vehicle values, in cultured neurons from ASM‐KO mice treated with vehicle, or with JNJ, PF, or URB in the presence or absence of AEA (**P_JNJ_ = 0.0013, *P_PF_ = 0.0143, ***P_URB_ = 0.0002, **P_JNJ + AEA_ = 0.0069, **P_PF + AEA_ = 0.0077, ***P_URB + AEA_ < 0.0001, *n *=* *3 independent cultures, one‐way ANOVA, Bonferroni *post hoc*).EMean ± SEM SM levels, expressed as percentage of vehicle values, in cultured neurons from ASM‐KO mice treated with vehicle, with PF, or with SR141716 + PF (*P_PF_ = 0.0109, *P_SR + PF_ = 0.0343, *n *=* *3 independent cultures, one‐way ANOVA, Bonferroni *post hoc*).FImmunofluorescences against the dendritic marker MAP2 of cultured neurons from ASM‐KO mice treated with vehicle, AEA, JNJ, PF, or URB. Scale bar, 50 μm.GMean ± SEM number of apoptotic cells measured by TUNEL assays in cultured neurons from ASM‐KO mice treated with H_2_O_2_ (as positive control), vehicle, AEA, JNJ, PF, or URB (*n *=* *3 independent cultures). Source data are available online for this figure.

The reduced levels of CB_1_ observed in ASM‐KO neurons, especially evident in the neuronal processes, and the reported ability of this receptor to activate NSM and hydrolyze SM in astrocytes (Sanchez *et al*, 2001) prompted us to assess the therapeutic potential of enhancing eCB action in ASMD. To this end, we first treated cultured ASM‐KO neurons with different doses of AEA. This treatment reduced SM levels in a dose‐dependent manner (Fig 3A). To determine whether NSM was involved in this effect, we treated ASM‐KO neurons with AEA in the presence or absence of the NSM inhibitor GW4869. This compound blocked the effect of AEA on SM levels (Fig 3B). Also consistent with the contribution of NSM, we observed increased levels of this SM‐degrading enzyme upon AEA treatment (Fig 3C). This treatment did not have significant effects on CB_1_ levels as assessed by Western blot (Appendix Fig S5A). Altogether, these observations point to the enhancement of eCB signaling as a potential intervention to increase NSM and reduce SM levels in neurons of ASM‐KO mice and ASMD patients.

To boost eCB signaling in disease settings, selective inhibition of eCB degradation is currently being considered as a better therapeutic avenue than direct CB_1_ receptor agonism since the former avoids the psychoactive side effects of the latter (Di Marzo, 2008; Pertwee, 2012). Thus, we tested the ability of three different FAAH inhibitors (FAAHi) to reduce SM levels in ASM‐KO neuronal cultures. Among the different currently available FAAHi, JNJ‐1661010 (JNJ), PF‐04457845 (PF), and URB‐597 (URB) were chosen because they can readily cross the blood–brain barrier and have shown good tolerability in preclinical and clinical studies (Ahn *et al*, 2009, 2011; D'Souza *et al*, 2019). All three FAAHi reduced SM levels, an effect that was independent of the presence of AEA in the incubation medium (Fig 3D). URB treatment also resulted in significant elevation of CB_1_ protein levels (Appendix Fig S5B). The CB_1_ antagonist SR141716 (Rinaldi‐Carmona *et al*, 1994) abrogated PF‐induced SM reduction (Fig 3E) without altering CB_1_ levels (Appendix Fig S5C). Two parameters were monitored to rule out the toxicity of the treatments. First, the morphology of the neurons was determined by staining with the dendritic marker MAP2 and was not affected by any of the treatments (Fig 3F). In addition, the number of apoptotic cells quantified by the TUNEL assay was not increased (Fig 3G). Incubation with AEA or the different FAAHi in the presence or absence of the NSM inhibitor or the CB_1_ antagonist did not have significant effects on SM levels in WT neurons (Appendix Fig S6). Overall, these findings demonstrate that activation of CB_1_ *via* FAAH inhibition in ASM‐KO neurons leads to activation of NSM and degradation of SM without any associated cell toxicity.

### Chronic oral treatment with the FAAHi PF improves behavior and extends lifespan in ASM‐KO mice

**Figure 4:**
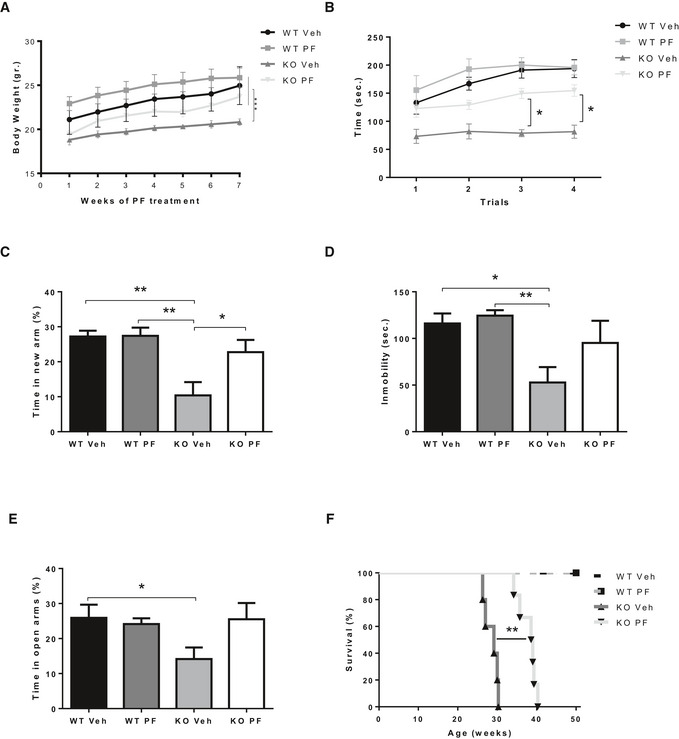
Chronic oral treatment with PF improves behavior and extends life span in ASM‐KO mice AWeekly mean ± SEM body weight of WT and ASM‐KO mice treated or not with PF (***P <* *0.0001, *n *=* *7 mice per group, statistics reflect the significant difference between the slope of the data in the PF‐treated ASM‐KO with respect to the other three groups, linear regression test).BMean ± SEM time spent on the rod in the 4 trials of the rotarod test by WT and ASM‐KO mice after 2 months of vehicle or PF treatment (*P_T3_ = 0.0488, *P_T4 _= 0.361, *n *=* *4 mice per group, two‐way ANOVA, Bonferroni *post hoc*).CMean ± SEM time spent in the new arm of the Y‐maze test by WT and ASM‐KO mice after 2 months of vehicle or PF treatment (**P_WTVeh Vs KO Veh_ = 0.0024, **P_WT PF Vs KO Veh_ = 0.0016, *P_KO PF Vs KO Veh_ = 0.0391, *n *=* *9 mice per group, two‐way ANOVA, Bonferroni *post hoc*).DMean ± SEM time of immobility in the tail suspension test of WT and ASM‐KO mice after 2 months of vehicle or PF treatment (*P_WTVeh Vs KO Veh _= 0.0198, **P_WT PF Vs KO Veh_ = 0.0067, *n *=* *6 mice per group, two‐way ANOVA, Bonferroni *post hoc*).EMean ± SEM time spent in the open arms of the elevated plus maze by WT and ASM‐KO mice after 2 months of vehicle or PF treatment (*P_WTVeh Vs KO Veh_ = 0.0342, *n *=* *7 mice per group, two‐way ANOVA, Bonferroni *post hoc*).FPercentage of survival with respect to weeks of age in WT and ASM‐KO mice treated with vehicle or PF (**P =* *0.0031, *n *=* *5 mice in WT vehicle, WT PF‐treated and KO vehicle, 6 mice in KO PF‐treated, Mantel–Cox) Source data are available online for this figure.

The ability to reduce SM levels and the lack of toxicity of FAAHi in cultured ASM‐KO neurons encouraged us to assess this strategy *in vivo*. Among the FAAHi used *in vitro*, we selected PF for *in vivo* studies owing to its high selectivity and efficacy, adequate oral bioavailability, optimal pharmacodynamic properties, and good tolerance in healthy subjects (Ahn *et al*, 2009, 2011; Li *et al*, 2012; D'Souza *et al*, 2019). PF treatment was started in WT and ASM‐KO mice at 6 weeks of age, when the initial disease symptoms (e.g., mild tremors) start to appear in the ASM‐KO mice. PF was administered at 0.3 mg/kg by oral gavage every 3 days for 8 weeks. This dose was chosen based on human studies (Li *et al*, 2012). Control ASM‐KO mice were treated in parallel with vehicle (0.9% NaCl‐0.05% DMSO). PF treatment improved body weight gain in the ASM‐KO mice (Fig 4A). Behavioral tests were also performed, confirming the motor and memory deficits in ASM‐KO mice. For example, vehicle‐treated ASM‐KO mice spent 57% less time than WT mice on the accelerating rod in the rotarod test, which measures motor abilities (Fig 4B), and 62% less time in the new arm of the Y‐maze test, which measures hippocampal‐dependent memory (Fig 4C). We also evaluated behavioral alterations not previously reported in ASM‐KO mice. Specifically, the time that ASM‐KO mice spent moving was reduced by 55% compared to WT littermates in the tail suspension test, which is indicative of a depressive state (Fig 4D). ASM‐KO mice also spent 11% less time in the open arm of the elevated plus maze, suggesting increased anxiety (Fig 4E). PF treatment of the ASM‐KO mice improved motor abilities and increased the time in the new arm in the Y‐maze (from 10% of the total time in the vehicle‐treated ASM‐KO mice to 23% in the PF‐treated group), the mobility in the tail suspension test (from 53 to 95 s), and the time spent in the open arm in the elevated plus maze (from 14% of the total time in the vehicle‐treated ASM‐KO mice to 26% in the PF‐treated group; Fig 4D and E). PF treatment in WT mice did not have significant effects in any of the tests analyzed (Fig 4B–E). These encouraging results obtained in behavior traits prompted us to extend PF treatment in a group of WT and ASM‐KO mice in order to monitor survival. PF treatment increased life span by 31% in the ASM‐KO mice (from 29 ± 2 weeks in vehicle‐treated ASM‐KO mice to 38 ± 2 weeks in the PF‐treated group), without having any effect on WT mice survival (Fig 4F).

### Chronic oral treatment with the FAAHi PF diminishes SM accumulation, lysosomal size increase, neurodegeneration, and inflammation in ASM‐KO mice

**Figure 5:**
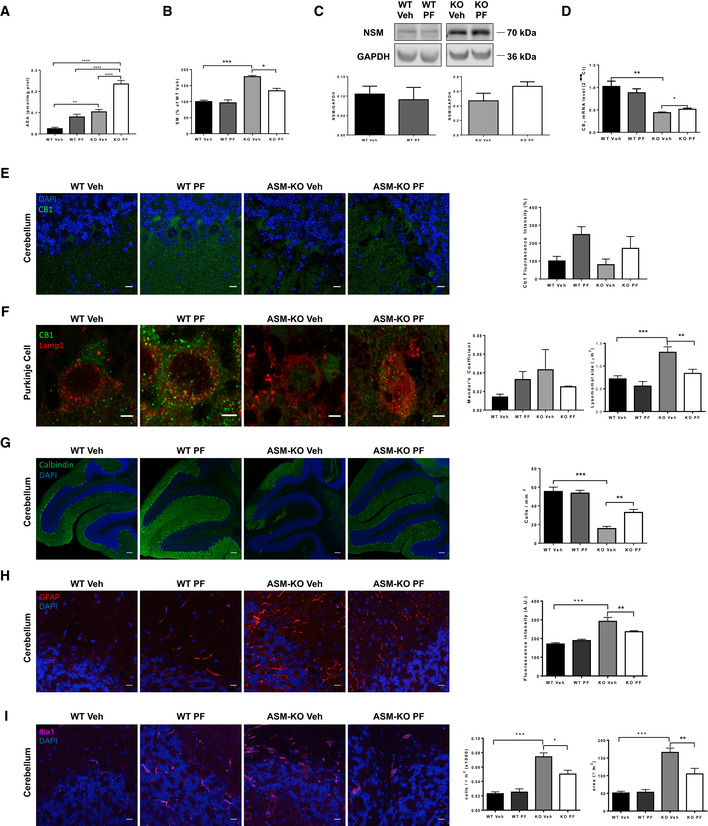
Chronic oral treatment with PF reduces cellular and molecular pathology in ASM‐KO mice AMean ± SEM AEA levels in cerebellar extracts from WT and ASM‐KO mice after 2 months of vehicle or PF treatment (**P_WT Veh vs KO Veh_ = 0.0063; ****P_WT Veh vs KO PF_ < 0.0001; ****P_WT PF vs KO PF_ < 0.0001; ****P_KO Veh vs KO PF_ < 0.0001, *n *=* *4 mice per group, one‐way ANOVA, Bonferroni *post hoc*).BMean ± SEM SM levels, expressed as percentage of vehicle values, in cerebellar extracts from WT and ASM‐KO mice after 2 months of vehicle or PF treatment (***P_WTVeh Vs KO Veh _< 0.0001, *P_KO Veh Vs KO PF_ = 0.0465), *n *=* *4 mice per group, one‐way ANOVA, Bonferroni *post hoc*).CWestern blot against NSM and GAPDH (used as loading control) and graph showing mean ± SEM NSM protein levels in cerebellar extracts from WT and ASM‐KO mice after 2 months of vehicle or PF treatment (*n *=* *3 mice per group, one‐way ANOVA, Bonferroni *post hoc*).DMean ± SEM CB_1_ mRNA levels in cerebellar extracts from WT and ASM‐KO mice after 2 months of vehicle or PF treatment (**P_WTVeh Vs KO Veh_ = 0.0026, *P_KO Veh Vs KO PF_ = 0.0345), *n *=* *4 mice per group, one‐way ANOVA, Bonferroni *post hoc*).EImmunofluorescence against CB_1_ in Purkinje cells of the cerebellum of WT and ASM‐KO mice after 2 months of vehicle or PF treatment. Graph shows mean ± SEM intensity associated with CB_1_, expressed as percentage of values in vehicle‐treated mice (*n *=* *4 mice per group, > 20 Purkinje cells per mouse, one‐way ANOVA, Bonferroni *post hoc*). Scale bar, 10 μm.FImmunofluorescence against CB_1_ and Lamp1 in the Purkinje cell layer of the cerebellum of WT and ASM‐KO mice after 2 months of vehicle or PF treatment. Graphs show Mander's coefficient for the co‐localization of CB_1_ with Lamp1 (left) and mean ± SEM lysosomal size (right; ***P_WT Veh vs KO Veh_ = 0.0002; **P_KO Veh vs KO PF_ = 0.0052, *n *=* *4 mice per group, > 20 lysosomes per mouse, one‐way ANOVA, Bonferroni *post hoc*). Scale bar, 5 μm.GImmunofluorescence against the Purkinje cell marker calbindin in the cerebellum of WT and ASM‐KO mice after 2 months of vehicle or PF treatment. Graph shows mean ± SEM number of calbindin‐positive cells per area (***P_WTVeh Vs KO Veh_ < 0.0001, **P_KO PF Vs KO Veh_ = 0.00642), *n *=* *4 mice per group, one‐way ANOVA, Bonferroni *post hoc*). Scale bar, 100 μm.HImmunofluorescence against the astrocytic marker GFAP in the cerebellum of WT and ASM‐KO mice after 2 months of vehicle or PF treatment. Graph shows mean ± SEM intensity associated with GFAP in arbitrary units (***P_WTVeh Vs KO Veh_ < 0.0001, **P_KO PF Vs KO Veh_ = 0.0083), *n *=* *4 mice per group, one‐way ANOVA, Bonferroni *post hoc*). Scale bar, 10 μm.IImmunofluorescence against the microglia marker Iba1 in the cerebellum of WT and ASM‐KO mice after 2 months of vehicle or PF treatment. Graph shows mean ± SEM number (left) and area (right) of Iba1‐positive cells (left: ***P_WTVeh Vs KO Veh_ < 0.0001, **P_KO PF Vs KO Veh_ = 0.0034; right: ***P_WTVeh Vs KO Veh_ < 0.0001, *P*_KO PF Vs KO Veh_ = 0.00123, *n *=* *4 mice per group, > 20 cells analyzed per mouse, one‐way ANOVA, Bonferroni *post hoc*). Scale bar, 10 μm. Source data are available online for this figure.

Other groups of ASM‐KO and WT mice were sacrificed after 8 weeks of PF treatment to monitor molecular and cellular hallmarks in different tissues. Mass spectrometry and HPLC showed increased levels of AEA in the cerebellum of ASM‐KO compared to WT mice and confirmed the ability of oral PF intake to increase the amount of AEA in cerebellar extracts of WT (3.2‐fold increase) and ASM‐KO (2.2‐fold increase) mice (Fig 5A). PF treatment also reduced SM levels by 33% in the ASM‐KO mice but not in the WT mice (Fig 5B). Western blot analysis showed a 42% enhancement of NSM expression in the cerebellar extracts of PF‐treated compared to vehicle‐treated ASM‐KO mice, which was not observed in the PF‐treated WT mice (Fig 5C). RT‐qPCR analysis showed 10% increase in cerebellar CB_1_ mRNA (Fig 5D) and immunofluorescence analysis a 90% increase in CB_1_ protein levels in the Purkinje cells of the PF‐treated compared to vehicle‐treated ASM‐KO mice (Fig 5E). Moreover, PF treatment limited the co‐localization of CB_1_ with the lysosomal marker Lamp1 (Mander's coefficient of 0.04 in vehicle‐treated and of 0.02 in PF‐treated ASM‐KO mice) and reduced by 36% the size of the lysosomes in the Purkinje cells of the ASM‐KO mice (Fig 5F). It also prevented the drastic loss (71%) of these cells in vehicle‐treated ASM‐KO mice by 31% (Fig 5G). Brain inflammation was analyzed by immunofluorescence against the astroglial marker GFAP and the microglial marker Iba‐1. While GFAP‐associated intensity was 80% higher in vehicle‐treated ASM‐KO compared to WT mice, PF treatment reduced this intensity by 45% (Fig 5H). While the number of microglial cells was increased by 200% in vehicle‐treated ASM‐KO compared to vehicle‐treated WT mice, PF treatment diminished this value by 30% (Fig 5I). Likewise, the acquisition of amoeboid morphology, indicative of activated microglia, was prevented by PF as indicated by the 30% decrease in microglial area found in PF‐treated compared to vehicle‐treated ASM‐KO mice (Fig 5I). PF treatment did not have significant effects in WT mice for any of the parameters analyzed, except for the aforementioned increment in AEA and CB_1_ levels (Fig 5).

To determine whether oral PF intake had any effect in peripheral organs, we measured SM levels in liver, spleen, and lungs, which are particularly affected in ASMD. PF treatment significantly reduced SM levels in the liver (35%) and spleen (30%). In lungs, we found a tendency to decrease of 25% (Appendix Fig S7). PF treatment had no effect on SM levels in the liver, spleen, and lungs of WT mice (Appendix Fig S7).

### Safety of acute oral treatment with the FAAHi PF at late disease stages in ASM‐KO mice

The findings described above demonstrate that chronic PF treatment of ASM‐KO mice starting at early stages of the disease (6 weeks of age) ameliorates biochemical, cellular, and behavioral anomalies, and also extends life span. However, in the clinical setting it may be difficult to diagnose and start a treatment at such a young age, before substantial SM storage has occurred. Older patients are likely to have much higher levels of SM, and rapid hydrolysis of this lipid to its downstream products ceramide and sphingosine can be toxic (Testi, 1996; Teichgraber *et al*, 2008; Hagen *et al*, 2009; Murray *et al*, 2015). Therefore, to evaluate whether hydrolysis of high levels of SM by PF‐induced activation of NSM might result in toxicity, we acutely treated 4‐month‐old ASM‐KO and WT mice with a single PF administration by oral gavage at a similar dose to that used in the chronic study (0.1 mg/kg) or at 10 (1 mg/kg)‐ and 50 (5 mg/kg)‐fold higher doses. No evident deleterious effects (i.e., death, lethargy, convulsions, weight, or hair loss) were observed during the following 48 h after administration of any of the doses.

**Figure 6:**
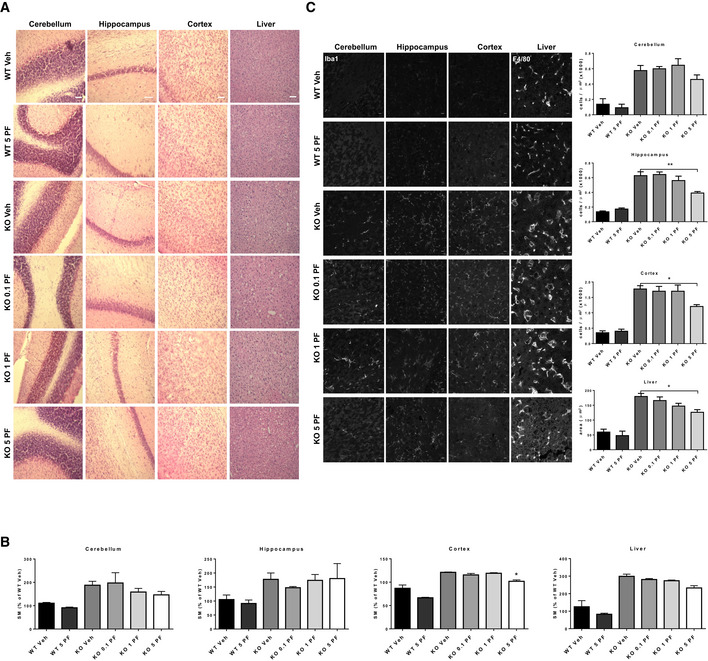
Acute oral treatment with PF at late disease stages is not toxic even at high doses AH&E staining of cerebellum, hippocampus, cortex, and liver from WT and ASM‐KO mice 48 h after one single administration of vehicle or the indicated doses of PF. Scale bar, 100 μm.BMean ± SEM SM levels, expressed as percentage of vehicle values, in cerebellar, hippocampal, cortical, and liver extracts from WT and ASM‐KO mice 48 h after one single administration of vehicle or the indicated doses of PF. (*P_KOVeh Vs KO 5_ = 0.0304, *n *=* *3 mice per group, one‐way ANOVA, Bonferroni *post hoc*)CImmunofluorescences against the microglia marker Iba1 in the cerebellum, hippocampus, and cortex or against the macrophage marker F4/80 in the liver from WT and ASM‐KO mice 48 h after one single administration of vehicle or the indicated doses of PF. Graphs show mean ± SEM number of microglia per area unit in cerebellum, hippocampus, and cortex or mean ± SEM area of macrophages in the liver (hippocampus: **P_KOVeh Vs KO 5_ = 0.0015; cortex: *P_KOVeh Vs KO 5_ = 0.0364; liver: *P_KOVeh Vs KO 5_ = 0.0152, *n *=* *6 mice per group, > 20 cells analyzed per mouse, one‐way ANOVA, Bonferroni *post hoc*). Scale bar, 10 μm. Source data are available online for this figure.

At this time point, mice were sacrificed, and cell toxicity was analyzed by hematoxylin and eosin staining in different brain areas (cerebellum, hippocampus, and cortex), as well as in the liver. PF treatment at any dose did not promote cell loss in any of the tissues analyzed. The acute treatments also did not alter the presence of foam cells, which are characteristic of ASMD (Fig 6A). The absence of significant cell death was confirmed by cleaved caspase 3 staining of the tissues (Appendix Fig S8). Lipid assays showed that PF treatment for 48 h did not affect SM levels at the low and intermediate doses. A tendency to SM reduction was observed at the highest dose (5 mg/kg) that was statistically significant (16%) in the cortex (Fig 6B). Inflammation was analyzed by staining of microglia with Iba1 in the different brain regions and by staining of macrophages with F4/80 in the liver. Accumulation of microglia and increased macrophage area were evident in the tissues of ASM‐KO compared to WT mice (Fig 6C). While the single PF administration at low and intermediate doses did not affect these parameters, administration of 5 mg/kg PF significantly reduced the number of microglia in the hippocampus and cortex, as well as macrophage area in the liver (Fig 6C). Single‐dose administration of PF did not have overt effects in WT mice (Fig 6A–C). These results supported the safety of PF administration at advanced stages of the disease and revealed an effect on SM levels in the cortex after a single administration of high PF doses.

### Downregulation of CB_1_ receptor and benefits of FAAHi treatment in NPC

**Figure 7:**
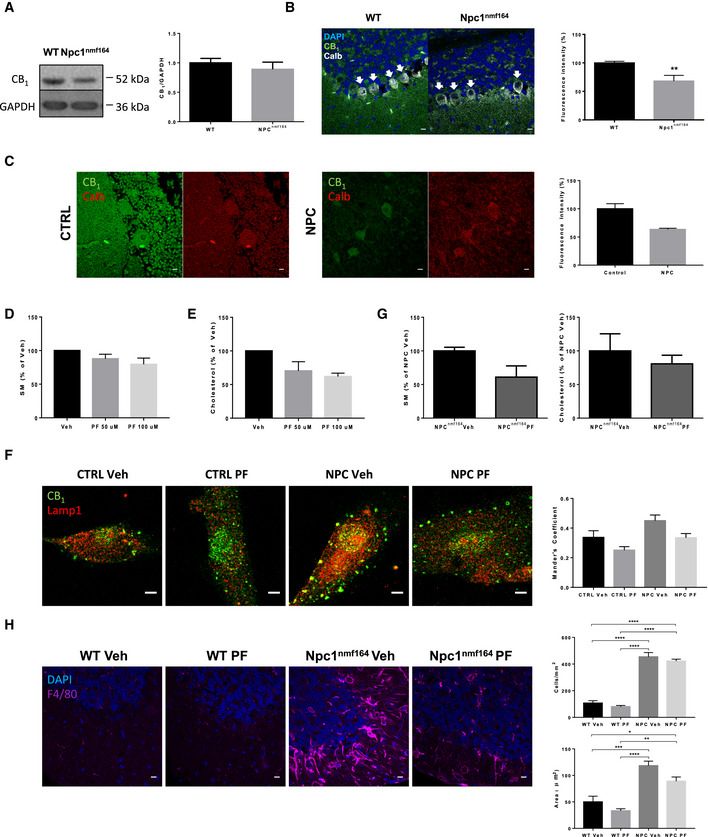
CB_1_ levels are reduced in NPC mouse model and patient brains, and FAAHi prevents aberrant phenotypes in NPC cells and mouse model AWestern blot against CB_1_ and GAPDH (used as loading control) and graph showing mean ± SEM CB_1_ protein levels in cerebellar extracts from WT and Npc1^nmf164^ mice (*n *=* *6).BImmunofluorescence images against CB_1_ and calbindin in the cerebellum of WT and Npc1^nmf164^ mice. DAPI stains cell nuclei. Graphs show mean ± SEM intensity associated with CB_1_ in the Purkinje cells (indicated by arrows) expressed in arbitrary units (**P =* *0.0085, *n *=* *5 mice per group, Student's *t*‐test). Scale bar, 10 μm.CImmunofluorescence images against CB_1_ in Purkinje cells of the cerebellum of age‐matched control and NPC‐affected children. Graph shows mean ± SEM intensity associated with CB_1_ in the Purkinje cells expressed as percentage of the control values (16 and 57 cells in control and NPC, respectively). Scale bar, 10 μm.DGraph shows mean ± SEM SM levels, expressed as percentage of vehicle values, in cultured fibroblasts from a NPC patient treated with vehicle or with PF at 50 or 100 μM (*n* = 2 different cultures)EGraph shows mean ± SEM cholesterol levels, expressed as percentage of vehicle values, in cultured fibroblasts from a NPC patient treated with vehicle or with PF at 50 or 100 μM (*n* = 2 different cultures)FImmunofluorescence images of CB_1_ and Lamp1 in cultured fibroblasts from control subject and NPC patient treated with vehicle or with PF at 100 μM. Graph shows mean ± SEM Mander's coefficient for the co‐localization of CB_1_ with Lamp1 (*n* = 2 different cultures). Scale bar, 5 μm.GGraphs show mean ± SEM SM and cholesterol levels expressed as percentage of the values obtained in the vehicle‐treated mice in the cerebellum of Npc1^nmf164^ after 48 h of a single dose of 5 mg/kg PF (*n* = 3 mice)HImmunofluorescence images against the microglia marker F4/80 in the cerebellum of WT and Npc1^nmf164^ after 48 h of a single dose of 5 mg/kg PF. Graphs show mean ± SEM number (upper) and area (lower) of F4/80-positive cells (upper graph: ****P <* *0.0001; lower graph: ***P_WT Veh vs NPC Veh_ = 0.0008; *P_WT Veh vs NPC PF_ = 0.0286; ****P_WT PF vs NPC Veh_ < 0.0001; **P_WT PF vs NPC PF_ = 0.0022, *n *=* *3 mice per group, > 20 cells analyzed per mouse, one‐way ANOVA, Bonferroni *post hoc*). Scale bar, 10 μm. Source data are available online for this figure.

Sphingomyelin accumulation also is a hallmark of other sphingolipid storage disorders including those with neurological involvement such as NPC (Lloyd‐Evans *et al*, 2008). While the ASM gene, *SMPD1*, is not affected in NPC cells, they do exhibit deficient ASM activity that was corrected by recombinant ASM treatment or *SMPD1* transfection. This, in turn, improved lipid (i.e., cholesterol) and protein trafficking defects (Devlin *et al*, 2010). To understand whether the impairment of eCB signaling is a shared pathological event between ASMD and NPC, and whether enhancement of this system could be considered as a common therapeutic strategy for this and potentially other sphingolipid storage disorders, we analyzed CB_1_ levels in the brain of an NPC mouse model and a patient, as well as the effects of FAAHi in NPC patient‐derived cells and the NPC mouse model. As in the ASM‐KO mice, we did not observe any significant reduction of CB_1_ protein levels by Western blot of cerebellar extracts in NPC^nmf164^ mice, which carry a point mutation in the *npc1* gene and mimic NPC (Maue *et al*, 2012), compared to WT littermates (Fig 7A). However, immunofluorescence analysis showed a 32% reduction of the protein levels in the Purkinje cells of the NPC^nmf164^ mice (Fig 7B). Access to fixed tissue from the cerebellum of unaffected and NPC‐affected children unveiled a 37% reduction in CB_1_ protein levels in the Purkinje cells of the NPC patient (Fig 7C). To determine the efficacy of FAAHi to reduce SM levels in the context of NPC, cultured fibroblasts from NPC patients were treated with 50 or 100 μM PF for 1 h. This intervention induced a dose‐dependent reduction of SM levels (13 and 21% reduction, respectively; Fig 7D). Moreover, and consistent with the reported influence of SM reduction on cholesterol amount in NPC cells (Devlin *et al*, 2010), we observed a PF dose‐dependent reduction on cholesterol levels (30 and 39% reduction, respectively; Fig 7E). In addition, PF treatment reduced the aberrantly increased CB_1_ localization in lysosomes in the NPC fibroblasts (Mander's coefficient changed from 0.45 in the NPC vehicle‐treated fibroblasts to 0.33 in the NPC PF‐treated fibroblasts; Fig 7F). To determine the effects of FAAHi treatment *in vivo*, we measured SM levels in the cerebellum of 3‐month‐old NPC^nmf164^ mice treated (or not) with a single, high dose (5 mg/kg) of PF for 48 h. This acute treatment diminished SM and cholesterol levels by 39 and 20%, respectively (Fig 7G). We did not observe a reduction in the number of microglia cells, which is increased in the cerebellum of vehicle‐treated NPC compared to WT mice, but we detected a significant 25% decrease in the area of the microglia from this single treatment (Fig 7H).

## Discussion

The results obtained in this study reveal multiple impacts of the eCB system in neurovisceral ASMD. On the one hand, the low levels of the CB_1_ receptor found in the neurons of ASM‐KO mice and a severe ASMD patient suggest a pathological contribution of this system. On the other hand, the benefits obtained with a CB_1_ signaling‐enhancing strategy in ASM‐KO mice support the eCB system as a novel therapeutic target for this fatal disease.

The analysis of the eCB system in the context of a disease model characterized by SM accumulation uncovers a feedback loop between this lipid and eCB action. Cell‐specific analysis revealed that, at least in ASMD, neurons are more susceptible to alterations in this feedback loop than astrocytes or microglia. This may also explain why the neuronal‐enriched eCB receptor CB_1_ is preferentially affected compared to the glial (immune cell)‐enriched eCB receptor CB_2_. Physiological amounts of SM at the neuronal plasma membrane are necessary to maintain not only the adequate levels of CB_1_ expression, but also its correct subcellular distribution. Aberrantly high SM amounts reduced both the mRNA and protein levels of CB_1_ in ASM‐KO neurons, similar to what was observed in WT neurons treated with SM. Hence, we demonstrate the existence of a mechanistic link between SM levels and CB_1_ expression, which might be reciprocally complemented by a CB_1_‐evoked reduction of SM levels through the activation of NSM, as previously described in astrocytes (Sanchez *et al*, 2001). This feedback loop would allow SM and CB_1_ to dynamically control each other's levels. We also observed that high SM promotes the loss of CB_1_ from the neuronal processes and induces its accumulation in lysosomes. This may lead to an enhanced degradation of this receptor that would conceivably contribute to the low levels of CB_1_ protein found in ASM‐KO neurons. CB_1_ sensitivity to membrane lipid changes had been previously illustrated by the effect of cholesterol depletion on CB_1_, but not CB_2_, signaling (Bari *et al*, 2005a,b). This effect was related to the specific presence of CB_1_, but not CB_2_, in lipid rafts (Bari *et al*, 2006; Maccarrone, 2008; Rimmerman *et al*, 2008). SM is, together with cholesterol, the main lipid component of rafts. Our findings thus reinforce the view that the composition of these membrane domains is a key factor for CB_1_ physiological actions and that this receptor contributes to regulate such composition through its influence on NSM. Another difference regarding the lipid sensitivity of CB_1_ and CB_2_ receptors is the interaction of the former, but not the later, with sphingosine. In fact, this SM metabolite has been suggested as an endogenous ligand for CB_1_ (Paugh *et al*, 2006). CB_1_ undergoes constitutive internalization and recycling, which explains its movement between the plasma membrane and intracellular compartments (Leterrier *et al*, 2004; Fletcher‐Jones *et al*, 2020). Exposure of CB_1_ to its agonists, for a prolonged time or at high concentrations, may promote the internalization of the receptor, the loss of its surface expression, and its exit from the recycling pathway to be targeted to lysosomes for degradation. This mechanism has been related to CB_1_ desensitization processes (Martini *et al*, 2007). It is possible that, in the context of ASMD, an increase in sphingosine due to SM accumulation, which has been indeed described in ASM‐KO neurons (Camoletto *et al*, 2009), mediates the observed loss of CB_1_ from neuronal process and its enrichment in lysosomes.

The crosstalk between eCB action and different neurotransmission systems contributes to the modulation of glutamatergic and gabaergic signaling. This underlies eCB influence in various neurobiological processes, including cognition and emotion (Zimmermann *et al*, 2019). Particular attention has been given to the link between CB_1_ and members of the type I metabotropic glutamate receptor (mGluR) family. For example, stimulation of postsynaptic mGluR_5_ in the striatum enhances diacylglycerol lipase activity, leading to 2‐AG production (Jung *et al*, 2007; Uchigashima *et al*, 2007). This eCB activates CB_1_ at the presynapses, thereby inhibiting synaptic transmission (Maccarrone *et al*, 2008; Lovinger, 2010). In a previous work, we have described mGluR_5_ deficiency in synapses of ASM‐KO mice (Arroyo *et al*, 2014). This may also contribute to the low CB_1_ amounts described in the present study. Alterations in CB_1_ and mGluR5/2‐AG coupling have been related with stress‐induced anxiety and psychiatric defects in fragile X syndrome (Maccarrone *et al*, 2010; Busquets‐Garcia *et al*, 2013; Chakrabarti *et al*, 2015). Future work will determine whether mGluR5/2‐AG/CB_1_ uncoupling may occur in ASMD, and whether this is linked to the behavioral alterations we have observed in ASM‐KO mice.

The eCB system also has important prohomeostatic and neuroprotective functions (Katona & Freund, 2012; Busquets‐Garcia *et al*, 2013; Mechoulam & Parker, 2013). Alterations in eCB signaling have been related to different neurodegenerative diseases (van der Stelt *et al*, 2005; Blazquez *et al*, 2011; Gomez‐Galvez *et al*, 2016; Aymerich *et al*, 2018), and hence, eCB modulation has gained therapeutic interest in recent years. Our findings link for the first time eCB system alterations with the lysosomal storage diseases, particularly those in which SM accumulates. Indeed, the abnormal downregulation of eCB signaling in ASMD may explain some of the symptoms of this disease, such as sleep and body temperature disturbances or cognitive impairment. While treatment with exogenous cannabinoids can enhance eCB signaling, these compounds are likely to have psychotropic effects, risk of tolerance, and addiction, and are difficult to dose. Currently, pharmacological modulation of eCB regulatory enzymes is considered a safer and more suitable therapeutic avenue (Di Marzo, 2008; Pertwee, 2012). Specifically, inhibition of the eCB‐degrading enzyme FAAH has been successfully tested in animal models for inflammation, chronic pain, and anxiety, without remarkable adverse effects (Kathuria *et al*, 2003; Russo *et al*, 2007; Ahn *et al*, 2009). Of note, eCB enhancement makes even more sense in the context of ASMD since the observed eCB hypofunction would diminish the risk of its pharmacological overactivation, which may have deleterious consequences. Moreover, we show that by enhancing CB_1_ function, we can counteract SM buildup, which is the main pathological hallmark in ASMD. All FAAHi tested in this study reduced SM levels in neuronal cultures with no evident toxicity. The *in vivo* oral administration of one of them (PF), at doses similar to those already used in healthy subjects (Ahn *et al*, 2011; Li *et al*, 2012), reduced SM content in brain and peripheral organs of ASM‐KO mice and prevented motor, cognitive, and psychiatric alterations, as well as neuroinflammation and neurodegeneration. There was also a significant increase in survival. This evidence strongly supports the use of FAAHi as a new ASMD treatment.

Several considerations should be taken into account when treating infantile neurovisceral ASMD patients. First, the time window for therapeutic intervention is very short, as, although the disease may be diagnosed months after birth, it progresses very quickly and generally leads to death by 3 years of age (McGovern *et al*, 2017). It is important to emphasize that here, we started the chronic PF treatment in ASM‐KO mice at 6 weeks of age, when SM levels are already elevated, and the first symptoms of the disease are evident. This reinforces the view that PF treatment would be beneficial even if started after the disease symptoms have already appeared. Second, the sudden conversion of the massive amount of accumulated SM at advanced stages of the disease into its proapoptotic and proinflammatory metabolites, including ceramide and sphingosine, is a potential hazard that has been observed in ASM‐KO mice previously when high doses of recombinant ASM were used (Murray *et al*, 2015). The effects we found upon acute PF administration to 4‐month‐old ASM‐KO mice argue against this possibility in our setting and support the safety of this compound even at very high doses (50‐fold higher than the equivalent dose already used in human studies). Remarkably, our acute study also revealed some extent of efficacy even after a single administration of high doses. Third, a therapy for severe neurovisceral ASMD should have a broad cellular and tissue impact, since ASM deficiency systemically affects all types of body cells. Our findings show the benefits of FAAHi treatment in all cells and tissues analyzed in ASM‐KO mice, including neurons, microglia, astrocytes, and macrophages, and different brain regions and peripheral organs, which, altogether, contribute to the significant extension of the life span. Importantly, an ever‐increasing number of intermediate cases between the infantile severe and the chronic visceral forms of ASMD are being diagnosed. This chronic neurovisceral form of the disease (often referred to as Niemann–Pick type A/B) is characterized by slower progression of neurological symptoms and prolonged survival compared to the infantile neurovisceral ASMD. We believe that the neuronal impact of FAAHi treatment may be useful for these patients in which ataxia, gross motor delays, and learning disabilities are commonly seen (McGovern *et al*, 2017).

Lastly, the feedback loop we have demonstrated herein between SM and CB_1_ prompted us to ask whether a similar SM‐induced CB_1_ downregulation might occur in other lysosomal storage disorders in which SM accumulates. If so, FAAHi treatment could be appropriate for these diseases as well. Supporting this view, here we observed low CB_1_ levels in neurons of a mouse model for NPC, in agreement with a recent report in brain tissue (Oddi *et al*, 2019). We extended this observation to the brain of an NPC patient and showed the efficacy of FAAH inhibition to reduce not only SM levels in NPC patient‐derived cells but also cholesterol accumulation, which is a pathologic hallmark of the disease (Devlin *et al*, 2010). Moreover, acute treatment with high oral doses of a FAAHi showed benefits in the brain of the NPC mouse model. Altogether, these data reinforce the suitability of eCB‐enhancing interventions as a potential common treatment for currently fatal sphingolipid storage disorders.

## Materials and Methods

### Study design

The goal of this study was to characterize alterations in the eCB system and the potential therapeutic value of eCB system enhancement in mouse models and patient‐derived cells and tissue of sphingolipid storage disorders. The sample size for each experiment is included in the figure legends. The number of mice used was selected on the basis of previous phenotyping analyses conducted in the same models and calculating the statistical power of the experiment. Mice were genotyped and randomly assigned to the experimental groups. No outliers were excluded in the study. Investigators performing the experiments were blinded to the mouse genotype. Sample collection, treatment, and processing information are included in the Results and Material and Methods sections. Investigators assessing and measuring results were blinded to the intervention.

### Antibodies

Antibodies against the following proteins were used in Western blots and for immunofluorescence analysis: calbindin (mouse, Swant, 300, dilution 1:500), CB_1_ (rabbit, Frontier Institute, AF380, 1:500), CB_2_ (mouse, R&D Systems, 352110, 1:500), F4/80 (rat, Abcam, ab6640, 1:500), GAPDH (mouse, Abcam, ab8245, 1:5,000), GFAP (mouse, Millipore, MAB3402, 1:1,000), Iba1 (rabbit, Wako, 019‐19741, 1:500), Lamp1 (rat, DSHB, 1D4B, 1:500), MAP2 (chicken, BioLegend, 822501, 1:500), NSM (rat, Santa Cruz, sc‐166637, 1:200), PSD‐95 (mouse, BD Transduction Laboratories, 610495, 1:500), and cleaved caspase 3 (Asp 175; rabbit polyclonal, Cell Signaling, 9661, 1:200). HRP‐conjugated rabbit anti‐mouse or anti‐rat and goat anti‐rabbit (DakoCytomation) were used as secondary antibodies in Western blots. Alexa‐conjugated rabbit anti‐mouse or anti‐rat and goat anti‐rabbit were used as secondary antibodies in immunofluorescence.

### CB_1_ Western blot

Western blots were performed following conventional protocols except for CB_1_ detection. In this case, samples were sonicated, homogenized in DTT‐loading buffer, and loaded without heating into the SDS–PAGE.

### Human samples

Formaldehyde‐fixed brain tissue from a 3‐year‐old ASMD patient, a 14‐day‐old NPC patient, and a 3‐year‐old control child were donated by the Wylder Nation Foundation (http://wyldernation.org), the Vall d′Hebron Hospital, and the Fundación CIEN Brain Bank (http://bt.fundacioncien.es), respectively.

Primary skin fibroblasts AG07471 (Gerontology Research Center Cell Culture), AG07310 (Collection Baltimore Longitudinal Study on Aging), and GM17912 (Niemann–Pick disease, type C1, NPC1, NPC1 gene; NPC1) were purchased from Coriell Institute for Medical Research (New Jersey, USA).

Informed consent was obtained from all human subjects, and the experiments conformed to the principles set out in the WMA Declaration of Helsinki and the Department of Health and Human Services Belmont Report.

### Mice

Breeding colonies were established from ASM heterozygous C57BL/6 mice (Horinouchi *et al*, 1995) kindly donated by Prof. E.H. Schuchman (Mount Sinai School of Medicine, New York, NY, USA) and from C57BL/6J NPC1^*nmf164*^ mice carrying a D1005G mutation in *Npc1* (Maue *et al*, 2012) purchased from Jackson Laboratories. Animals were grouped by genotype and gender. The mice were kept in a 12‐h light/dark cycle in a SPF (specific pathogen free) room. Male/female ASM‐KO, Npc1^*nmf164*^, and WT littermates were analyzed between 1.5 and 9 months of age. No gender‐dependent differences were observed in any of the results. Experiments were conducted according to the ARRIVE guidelines. Procedures followed European Union guidelines and were approved by the CBMSO and Comunidad de Madrid Animal Welfare Committees (PROEX 175/17).

### Neuronal cultures

Primary cultures of hippocampal or cortical neurons were prepared from day 17.5 ASM‐KO and WT embryos as described (Dotti *et al*, 1988). Neurons were kept under 5% CO_2_ at 37°C in Neurobasal Medium (Gibco) plus B27 Supplement (Gibco) and GlutaMAX (Gibco) until 7 days *in vitro* (DIV). The medium was then replaced with Neurobasal Medium plus B27 without GlutaMAX.

### *In vitro* treatments

Primary cultures of hippocampal or cortical neurons were prepared from WT and ASM‐KO mouse embryos as described above. Where indicated AEA (Sigma, dissolved in ethanol) was added to the medium at 5–100 μM for 1 h, SM (Sigma, dissolved in ethanol) at 40 μM for 48 h, SMase (Sigma, dissolved in phosphate buffer saline) at 0.1 U/100 μl for 24 h, the CB_1_ inhibitor SR141716 (Sigma, dissolved in DMSO) at 1 μM for 1 h, the NSM inhibitor GW4869 (Cayman Chemical, dissolved in DMSO) at 15 μM for 1 h, and the autophagy inhibitor bafilomycin (Enzo Life Sciences, dissolved in DMSO) at 0.1 μM for 24 h. In some instances, the following FAAH inhibitors were added for 1 h at a final concentration of 50 μM from stocks dissolved in DMSO: JNJ‐1661010 (Sigma), PF‐04557845 (MedChem), and URB597 (Selleckchem).

Primary skin fibroblasts were cultured in DMEM. PF‐04557845 (MedChem) was added to the medium for 1 h at final concentrations of 50 and 100 μM from stocks dissolved in DMSO.

### *In vivo* treatments

PF‐04557845 (kindly provided by Pfizer) was solubilized in DMSO and dissolved in saline buffer at 0.1, 0.5, or 5 mg/kg and was administered to the mice by oral gavage. As a control, WT and ASM‐KO received the same volume of vehicle (saline buffer). The chronic study was initiated at 6 weeks of age and prolonged for another 8 weeks or until death in the survival experiment. PF or vehicle was administered every 3 days. In the acute study, a single administration of PF or vehicle by oral gavage was given to WT and ASM‐KO mice at 4 months of age or to WT and NPC^nmf164^ mice at 3 months of age. Mice were sacrificed 48 h after administration.

### Immunofluorescence

Cultured neurons at 14 DIV were fixed in 4% PFA 0.12M sucrose and incubated overnight with primary antibodies and subsequently for 1 h with Alexa 488‐ or Alexa 555‐conjugated secondary antibodies. Images were taken using a confocal microscope LSM510 (Zeiss) or LSM800 (Zeiss). The background was subtracted and the image quantified using the Fiji software (Schindelin *et al*, 2012). The Mander's coefficient for CB_1_ co‐localization with Lamp1 was obtained by applying the threshold IsoData and using the JACoP plugin.

Mouse brains were dissected, fixed in 4% PFA 0.12M sucrose, and cryoprotected for 24 h in 30% sucrose phosphate buffer saline. The tissue was then frozen in Tissue‐Tek optimal cutting temperature compound (Sakura Finetek, Torrance, CA, USA), and 30‐μm sagittal sections were obtained with a cryostat (CM 1950 Ag Protect freezing: Leica, Solms, Germany). The sections were incubated overnight at 4°C with the primary antibodies and then with the corresponding Alexa‐conjugated secondary antibodies. Finally, the sections were incubated for 10 min with DAPI (Merck) or TOPRO (Thermo Fisher), washed, and mounted with ProLong Gold Antifade (Thermo Fisher). Images were obtained on a confocal LSM710 or LSM800 microscope (Zeiss) and quantified using the Fiji software.

Human sections were deparaffinized in decreasing concentrations of ethanol and xylene and subjected to heat‐mediated antigen retrieval in Tris–EDTA buffer (pH 9.0). Afterward, the same protocol performed for mouse sections was carried out. Finally, sections were dehydrated by immersion in increasing concentrations of ethanol and xylene and mounted using FluorSave (Merck).

### Hematoxylin and eosin staining

Mouse brain and liver sections were stained using conventional protocols (Feldman & Wolfe, 2014).

### Lipid measurements

Sphingomyelin levels were measured according to protocols modified from Hojjati and Jiang (2006). Lipid extracts were dried in the presence of Thesit, and SM was converted into peroxide by incubation with sphingomyelinase, alkaline phosphatase, and choline oxidase. Peroxide was measured fluorimetrically in the presence of peroxidase and homovanillic acid (Van Veldhoven *et al*, 1997). Cholesterol levels were determined using the Amplex^®^ Red Cholesterol Assay Kit (Invitrogen). AEA and 2‐AG were measured by LC‐HRMS analysis using an Acquity ultra‐high‐performance liquid chromatography (UHPLC) system (Waters, USA) connected to a time‐of‐flight (LCT Premier XE) detector.

### Quantitative RT–PCR

Total RNA from WT and ASM‐KO brains was extracted with the TRIzol Reagent (Ambion/RNA Life Technologies Co.) following the manufacturer's instructions using the RNeasy Mini Kit (Qiagen, Hilden, Germany). RNA was quantified by absorbance at 260 nm using a NanoDrop ND‐100 (Thermo scientific; Thermo Fisher Scientific Inc.). Retrotranscription to first‐strand cDNA was performed using RevertAid H Minus First‐Strand cDNA Synthesis Kit (Thermo scientific; Thermo Fisher Scientific Inc.). 10 ng of synthesized cDNA was used to perform fast qPCR using the GoTaq qPCR Master Mix (Promega Co., Madison, WI, USA) in ABI PRISM 7900HT SDS (Applied Biosystems; Life Technologies Co.) following the manufacturer's instructions. The primers purchased from Sigma‐Aldrich (mouse CB_1_: forward: 5′‐GCACCTTCACGGTTCTGG‐3′ and reverse: 5′‐GACTGCGGGAGTGAAGGAT‐3′; mouse CB_2_: forward: 5′‐CTACAAAGCTCTAGTCACCCGT‐3′ and reverse: 5′‐CCATGAGCGGCAGGTAAGAAA‐3′) were used at 0.5 μM final concentration. Three housekeeping genes (Gapdh, GusB, and Pgk1) were used as endogenous controls.

### Behavioral tests

The rotarod test was performed in an accelerating rotarod apparatus (Ugo Basile, Varese, Italy), on which the mice were trained for 2 days at a constant speed: the first day—four times at 4 r.p.m. for 1 min; and on the second day—four times at 8 r.p.m. for 2 min. On the third day, the rotarod was set to progressively accelerate from 4 to 40 r.p.m. for 5 min, and the mice were tested four times. During the accelerating trials, the latency to fall from the rod was measured.

The Y‐maze was performed as previously described (Cognato *et al*, 2010). During the first training trial (lasting 7 min), the mice only explored two arms (the initial arm and one other arm), maintaining the third or novel arm closed. After 1 h, mice were placed in the same starting arm with free access to all three arms for 5 min. The time spent in the novel arm was counted and expressed as a percentage of the total exploration time.

The tail suspension test (Can *et al*, 2012) was performed by suspending the mice through the tail over a 6‐min period. The time in which the animals were immobile was measured and expressed as percentage of the total time.

The elevated plus maze was performed as described (Lister, 1987). Each mouse was placed in the central square of the maze facing one of the closed arms and was recorded for 5 min. The time spent in the open arms of the maze was measured and expressed as a percentage of the total time.

### Statistical analysis

Data from at least three different experimental groups were quantified and presented as the mean ± SEM. Normality of the data was tested using the Shapiro–Wilk test. For two‐group comparisons, the Mann–Whitney *U*‐test for non‐parametric data or a two‐sample Student's *t*‐test for data with parametric distribution was used. For multiple comparisons, data with a normal distribution were analyzed by one‐way ANOVA followed by Bonferroni, Tukey, or Games–Howell *post hoc* test. The statistical significance of non‐parametric data was determined by the Kruskal–Wallis test to analyze all experimental groups. The Mann–Whitney *U*‐test was used to analyze paired genotypes, applying the Bonferroni correction. Linear regression test with 95% confidence interval was used to analyze slope differences. *P*‐values (*P*) < 0.05 were considered significant. In the figures, asterisks indicate the *P*‐values: *< 0.05; **< 0.005; ***< 0.001. GraphPad Prism 5.0 software (GraphPad Software, La Jolla, CA, USA) was used for all statistical analysis.

## Data and software availability

This study includes no data deposited in external repositories.

## Author contributions

AB and AT‐Z designed, performed, and analyzed the experiments. JC measured eCB levels. MG and EHS reviewed the data and provided advice. MDL designed the experiments, analyzed the data, and wrote the manuscript.

## Conflict of interest

MDL, AB, and EHS are co‐inventors on a patent describing the use of FAAHi for the treatment of lysosomal storage diseases.

## Supporting information

Appendix

Source Data for Appendix

Revieew Process File

Source Data for Figure 1

Source Data for Figure 2

Source Data for Figure 3

Source Data for Figure 4

Source Data for Figure 5

Source Data for Figure 6

Source Data for Figure 7
