# Supplementary material for: Inhibition of fatty acid amide hydrolase prevents pathology in neurovisceral acid sphingomyelinase deficiency by rescuing defective endocannabinoid signaling
Source: EMBO Mol Med. 2020 Oct 5;12(11):e11776. doi: 10.15252/emmm.201911776 (PMC7645369; doi:10.15252/emmm.201911776)
Supplement: Supplementary file 1 — Appendix [file EMMM-12-e11776-s001.pdf]

Appendix Supplementary Figures

Inhibition of Fatty Acid Amide Hydrolase Prevents Pathology in Neurovisceral Acid Sphingomyelinase Deficiency by Rescuing Defective Endocannabinoid Signaling

Adrián Bartoll, Ana Toledano-Zaragoza, Josefina Casas,

Manuel Guzmán, Edward H. Schuchman, María Dolores Ledesma

Table of content

Appendix Figure S1. CB_1 levels in hippocampus and cortex of WT and ASM-KO mice

Appendix Figure S2. Increased amount of astrocytes and microglia in the cerebellum of ASM-KO mice.

Appendix Figure S3. CB_1 levels in neurons of the medium bulb of control and infantile neurovisceral ASMD-affected children

Appendix Figure S4. Effects of SMase treatment in WT cultured neurons

Appendix Figure S5. Effects of AEA and FAAHi treatments on CB_1 protein levels measured by Western blot in ASM-KO cultured neurons

Appendix Figure S6. Effects of AEA and FAAHi treatments on SM levels in WT cultured neurons

Appendix Figure S7. SM levels in peripheral organs of WT and ASM-KO mice after chronic treatment with PF.

Appendix Figure S8. Cell death in the cerebellum of WT and ASM-KO mice after acute treatment with PF.

Appendix Figure S1

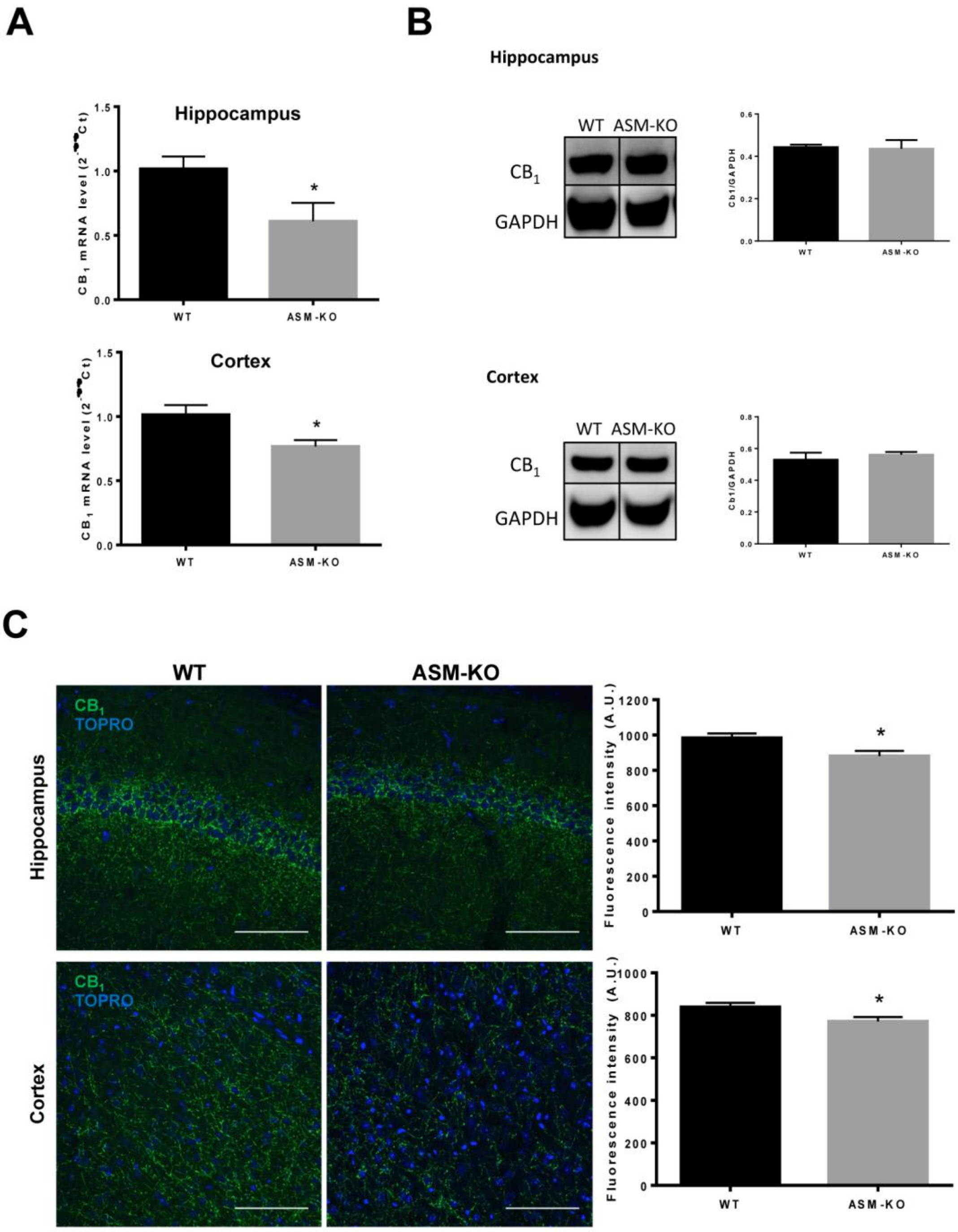

Appendix Figure S1. CB1 levels in hippocampus and cortex of WT and ASM-KO mice

A. Mean ± SEM CB1 mRNA levels in extracts from the hippocampus and prefrontal cortex of WT and ASM-KO mice (P_{hip} = 0.0464; P_{cor} =0.0154; *n* = 6 mice per group, Student's *t*-test).

B. Western blot against CB1 and GAPDH (used as loading control). Images belong to the same blot but to non-consecutive lanes as indicated by the boxes. Graphs

show mean ± SEM CB_1 protein levels in extracts from the hippocampus and prefrontal cortex of WT and ASM-KO mice (*n* = 6 mice per group).

C. Immunofluorescence images against CB_1 in the hippocampus and prefrontal cortex of WT and ASM-KO mice. TOPRO stains cell nuclei. Graphs show mean ± SEM intensity associated to CB_1 per area, expressed in arbitrary units (P_{hip} = 0.0109; P_{cor}= 0.0193, *n* = 4 mice per group, Student's *t*-test). Scale bar, 100 µm.

Appendix Figure S2

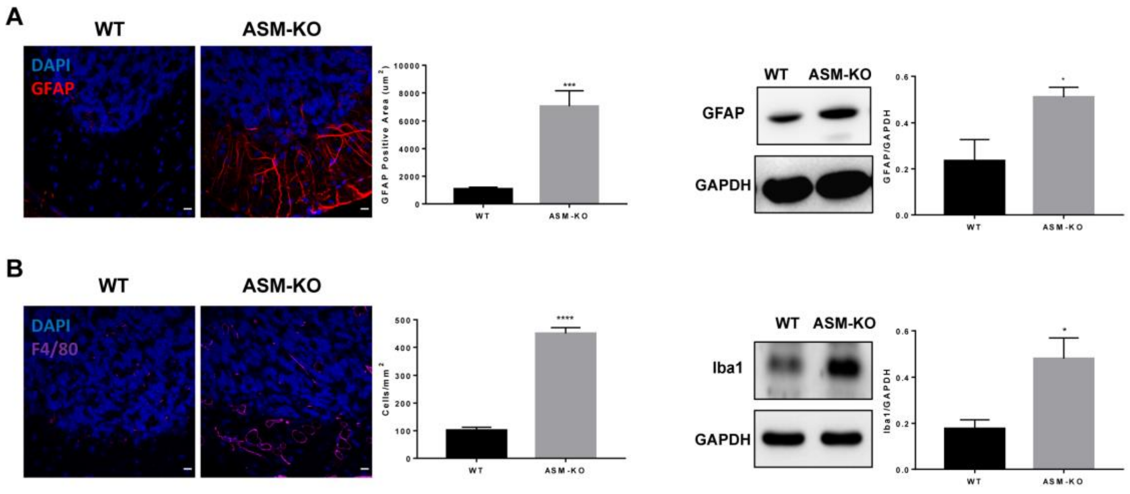

Appendix Figure S2. Increased amount of astrocytes and microglia in the cerebellum of ASM-KO mice.

A. To the left, immunofluorescence images against the astrocytic marker GFAP and graph showing mean ± SEM intensity associated to GFAP per area unit in the cerebellum of WT and ASM-KO mice (*P*=0.0008, *n* = 5 mice per group, Student's *t*-test). Scale bar, 10 μm. To the right, Western blot against GFAP and GAPDH (used as loading control) and graph showing mean ± SEM GFAP protein levels in cerebellar extracts of WT and ASM-KO mice (*P*=0.045, *n* = 3 mice per group, Student's *t*-test)

B. To the left, immunofluorescence images against the microglia marker F4/80 and graph showing mean ± SEM number of F4/80 positive cells per area unit. (*P*<0.0001, *n* = 5 mice per group, Student's *t*-test). Scale bar, 10 μm. To the right, Western blot against the microglia marker Iba-1 and GAPDH (used as loading control) and graph showing mean ± SEM Iba-1 protein levels in cerebellar extracts of WT and ASM-KO mice (*P*=0.014, *n* = 3 mice per group, Student's *t*-test)

Appendix Figure S3

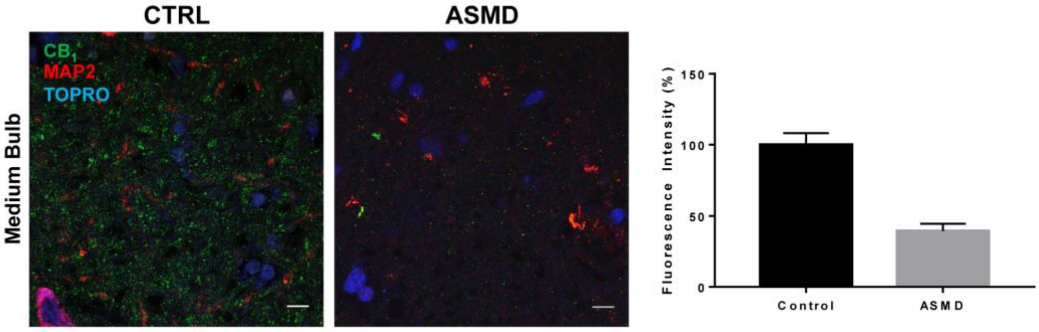

Appendix Figure S3. CB1 levels in neurons of the medium bulb of control and infantile neurovisceral ASMD-affected children.

Immunofluorescence images against CB1 and the neuronal marker MAP2 in the medium bulb of age-matched control and ASMD-affected children. TOPRO stains cell nuclei. Graph shows mean ± SEM intensity associated to CB1 in the MAP2-positive cells expressed as percentage of the control values. Scale bar, 10 μm.

Appendix Figure S4

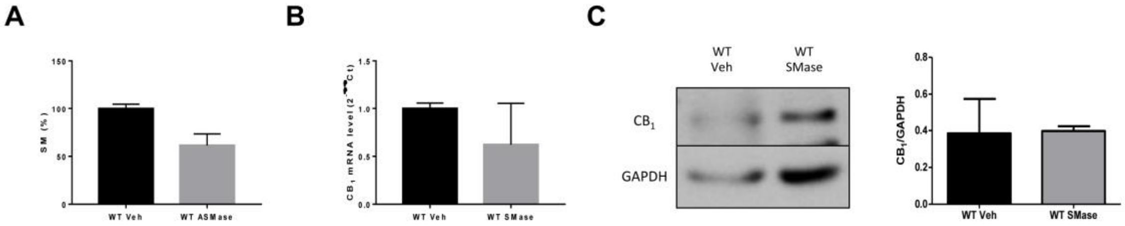

Appendix Figure S4. Effects of SMase treatment in WT cultured neurons

A. Mean ± SEM SM levels in cultured neurons from WT mice incubated with exogenous SMase expressed as percentage of the values obtained in vehicle-treated cultures (*n* = 3 independent cultures).

B. Mean ± SEM CB1 mRNA levels in cultured neurons from WT mice incubated or not with exogenous SMase (*n* = 3 independent cultures).

C. Western blot against CB1 and GAPDH (used as loading control). Graph shows mean ± SEM CB1 protein levels normalized to GAPDH in cultured neurons from WT mice incubated or not with exogenous SMase (*n* = 3 independent cultures).

Appendix Figure S5

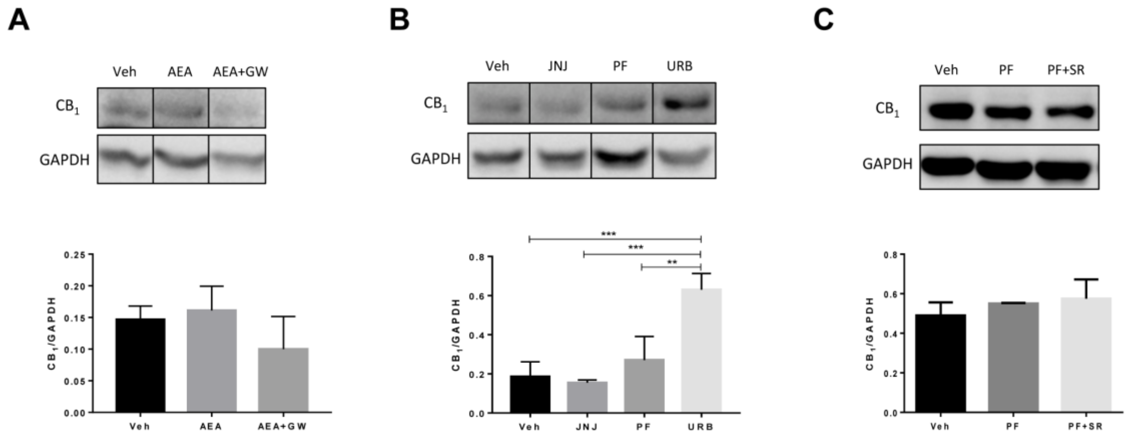

Appendix Figure S5. Effects of AEA and FAAHi treatments on CB_1 protein levels measured by Western blot in ASM-KO cultured neurons

- **A.** Western blot against CB_1 and GAPDH (used as loading control). Images belong to the same blot but to non-consecutive lanes as indicated by the boxes. Graph shows mean ± SEM CB_1 protein levels in ASM-KO neurons incubated with vehicle, AEA or AEA and GW (*n*= 3 independent cultures).
- **B.** Western blot against CB_1 and GAPDH (used as loading control). Images belong to the same blot but to non-consecutive lanes as indicated by the boxes. Graph shows mean ± SEM CB_1 protein levels in ASM-KO neurons incubated with vehicle, JNJ, PF or URB ($P_{\text{Veh vs. URB}} = 0.0008$; $P_{\text{JNJ vs. URB}} = 0.0005$; $P_{\text{PF vs. URB}} = 0.030$; *n*= 3 independent cultures, one - way ANOVA, Tukey *post hoc*).
- **C.** Western blot against CB_1 and GAPDH (used as loading control). Graph shows mean ± SEM CB_1 protein levels in ASM-KO neurons incubated with vehicle, PF or PF and SR (*n*=3 independent cultures).

Appendix Figure S6

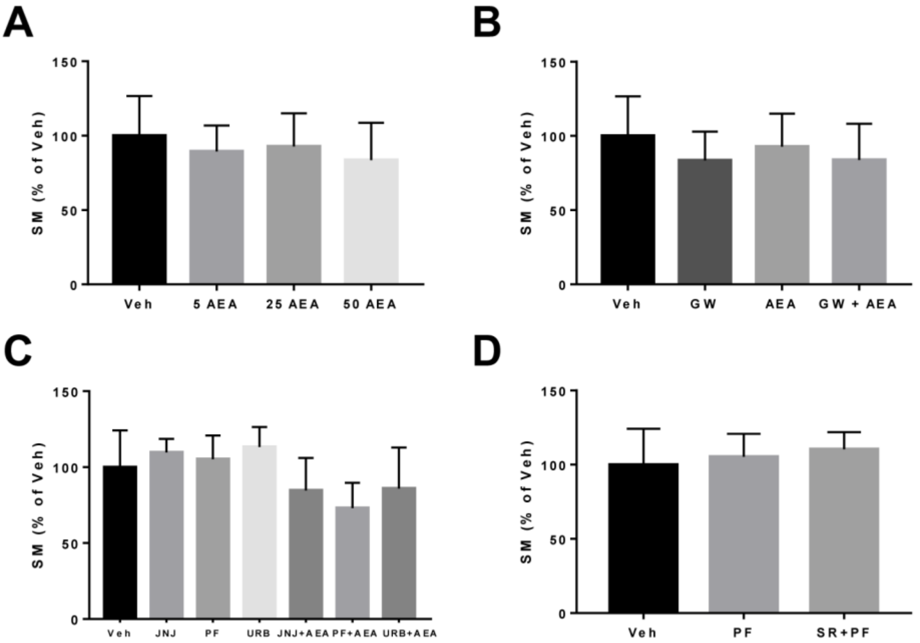

Appendix Figure S6. Effects of AEA and FAAHi treatments on SM levels in WT cultured neurons

A. Mean ± SEM SM levels in cultured neurons from WT mice treated with vehicle or with the indicated concentrations of AEA expressed as percentage of the values obtained in the vehicle-treated cultures (*n* = 3 independent cultures).
B. Mean ± SEM SM levels in cultured neurons from WT mice treated with vehicle, the inhibitor of NSM GW, AEA or the combination of GW and AEA, expressed as percentage of values obtained in the vehicle-treated cultures (*n* = 3 independent cultures).
C. Mean ± SEM SM levels in cultured neurons from WT mice treated with vehicle, or with JNJ, PF or URB in the presence or absence of AEA, expressed as percentage of values obtained in the vehicle-treated cultures (*n* = 3 independent cultures).
D. Mean ± SEM SM levels in cultured neurons from WT mice treated with vehicle, with PF or with SR141716 + PF, expressed as percentage of values obtained in the vehicle-treated cultures (*n* = 3 independent cultures).

Appendix Figure S7

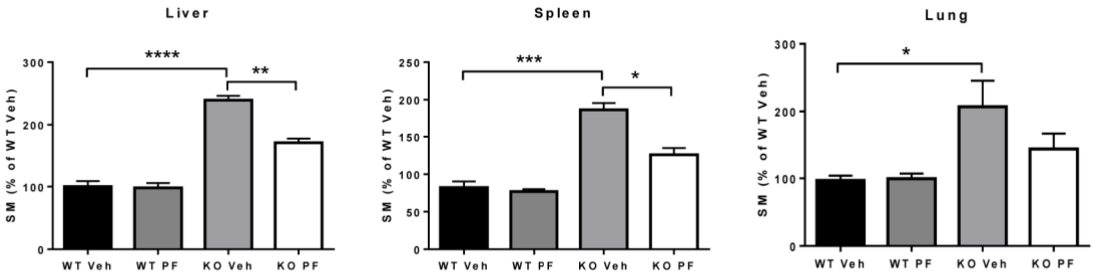

Appendix Figure S7. SM levels in peripheral organs of WT and ASM-KO mice after chronic treatment with PF. Mean ± SEM SM levels, expressed as percentage of WT vehicle- treated values, in liver, spleen and lung extracts from WT and ASM-KO mice after two months of vehicle or PF treatment ($P_{\text{Liver WTVeh Vs KO Veh}} < 0.0001$, $P_{\text{Liver KO Veh Vs KO PF}} = 0.0013$; $P_{\text{Spleen WTVeh Vs KO Veh}} < 0.0001$, $P_{\text{Spleen KO Veh Vs KO PF}} = 0.0018$; $P_{\text{Lung WTVeh Vs KO Veh}} = 0.0163$; $n = 4$ mice per group, one - way ANOVA, Bonferroni *post hoc*)

Appendix Figure S8

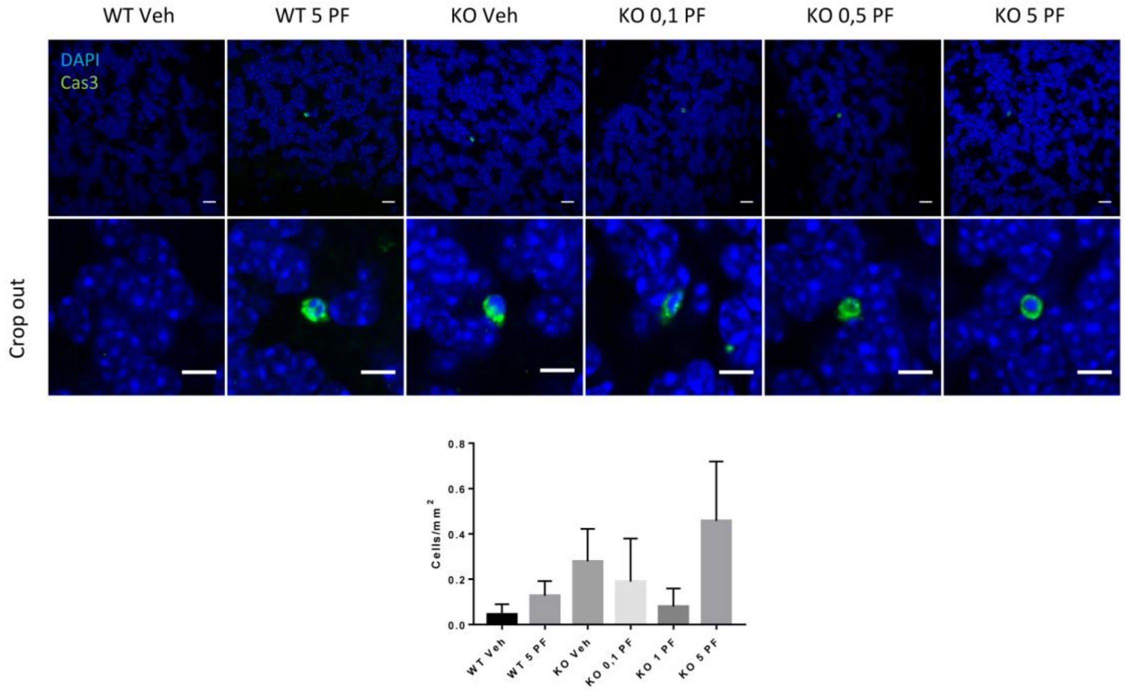

Appendix Figure S8. Cell death in the cerebellum of WT and ASM-KO mice after acute treatment with PF.

Immunofluorescence images against cleaved caspase3, a cell death marker, in the cerebellum of WT and ASM-KO mice 48 hours after one single administration of vehicle or the indicated doses of PF. DAPI stains cell nuclei. Scale bar, 10 μm. Lower

panels show high magnification images of the cleaved-caspase3 positive cells in the upper panels. Graph shows mean ± SEM number of caspase3 positive cells per mm^2 (n = 3 mice per group).
