# Supplementary figures and images for: Inhibition of fatty acid amide hydrolase prevents pathology in neurovisceral acid sphingomyelinase deficiency by rescuing defective endocannabinoid signaling
Source: EMBO Mol Med. 2020 Oct 5;12(11):e11776. doi: 10.15252/emmm.201911776 (PMC7645369; doi:10.15252/emmm.201911776)

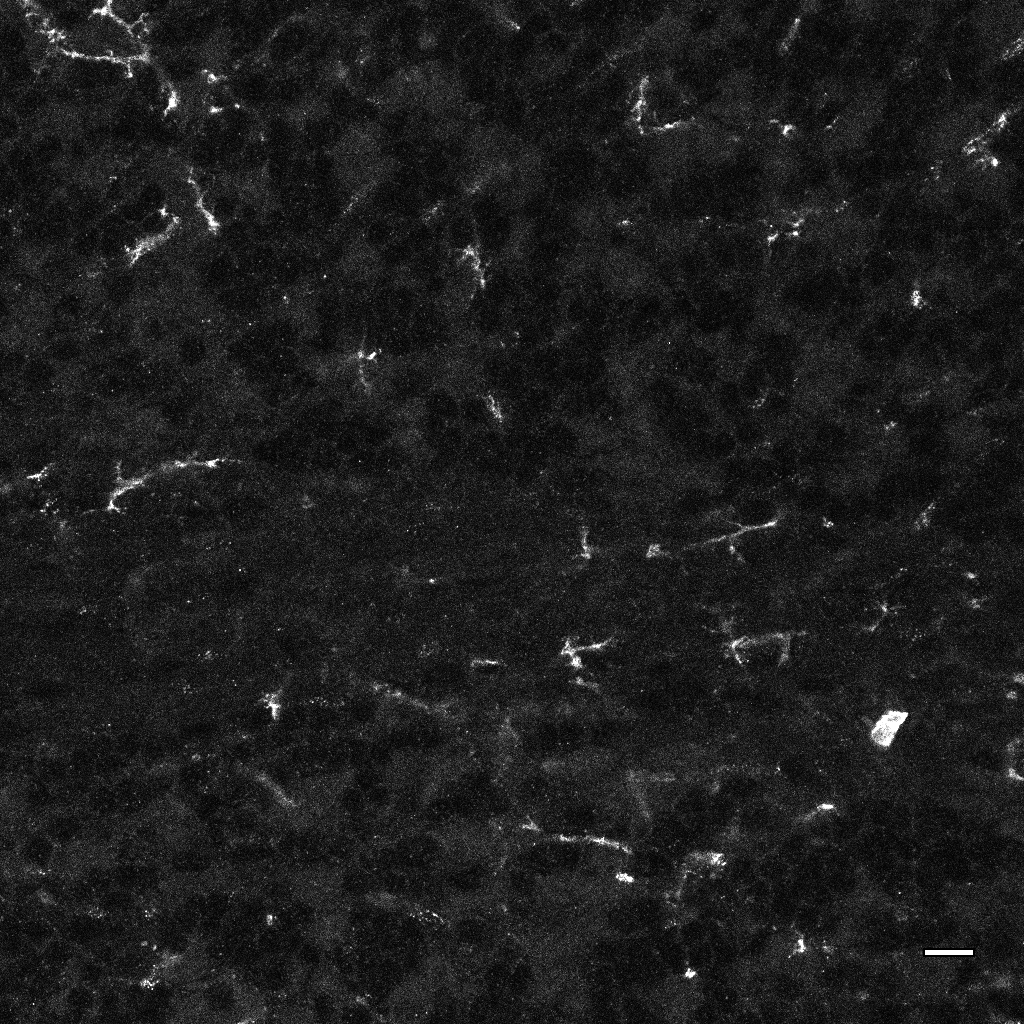

Supplement: Supplementary file 9 — Source Data for Figure 6 [file EMMM-12-e11776-s007.zip › Fig 6 source data 1 September/01kocer.tif]

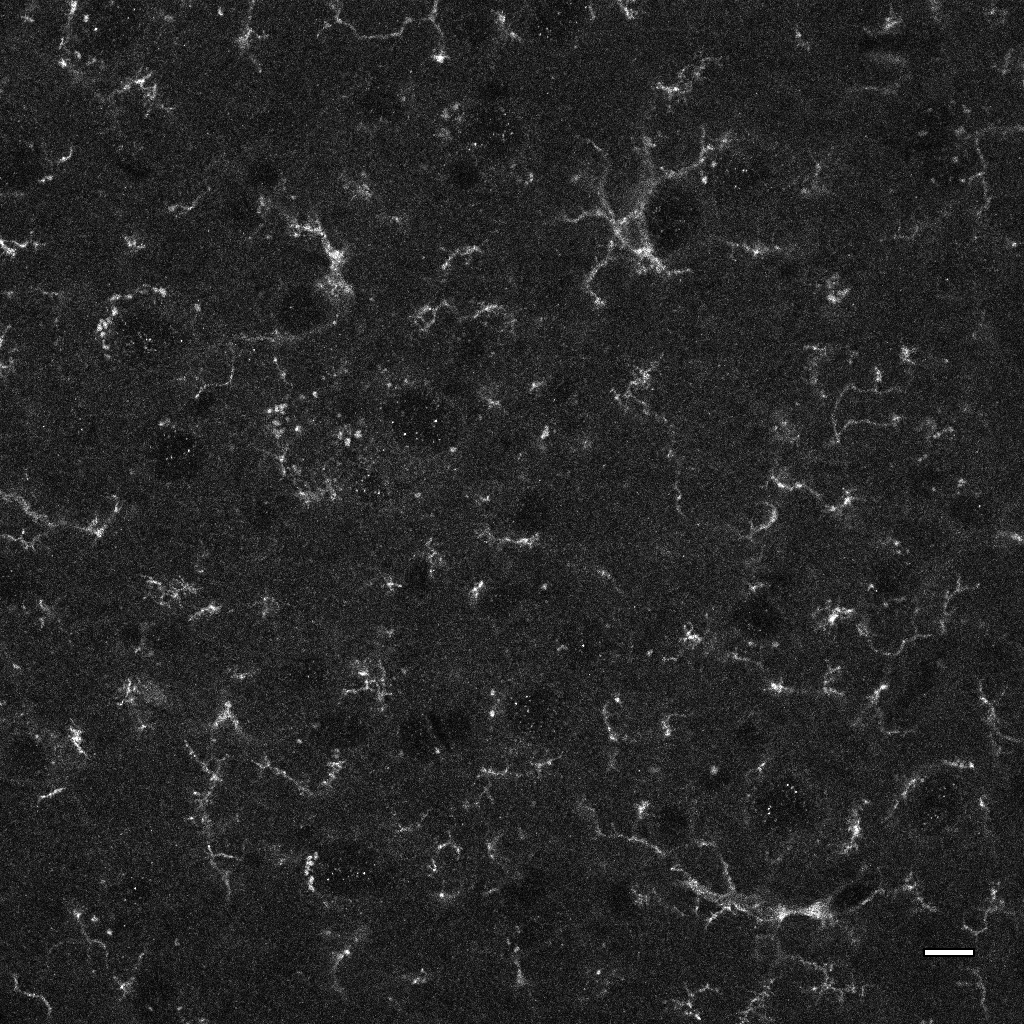

Supplement: Supplementary file 9 — Source Data for Figure 6 [file EMMM-12-e11776-s007.zip › Fig 6 source data 1 September/01kocx.tif]

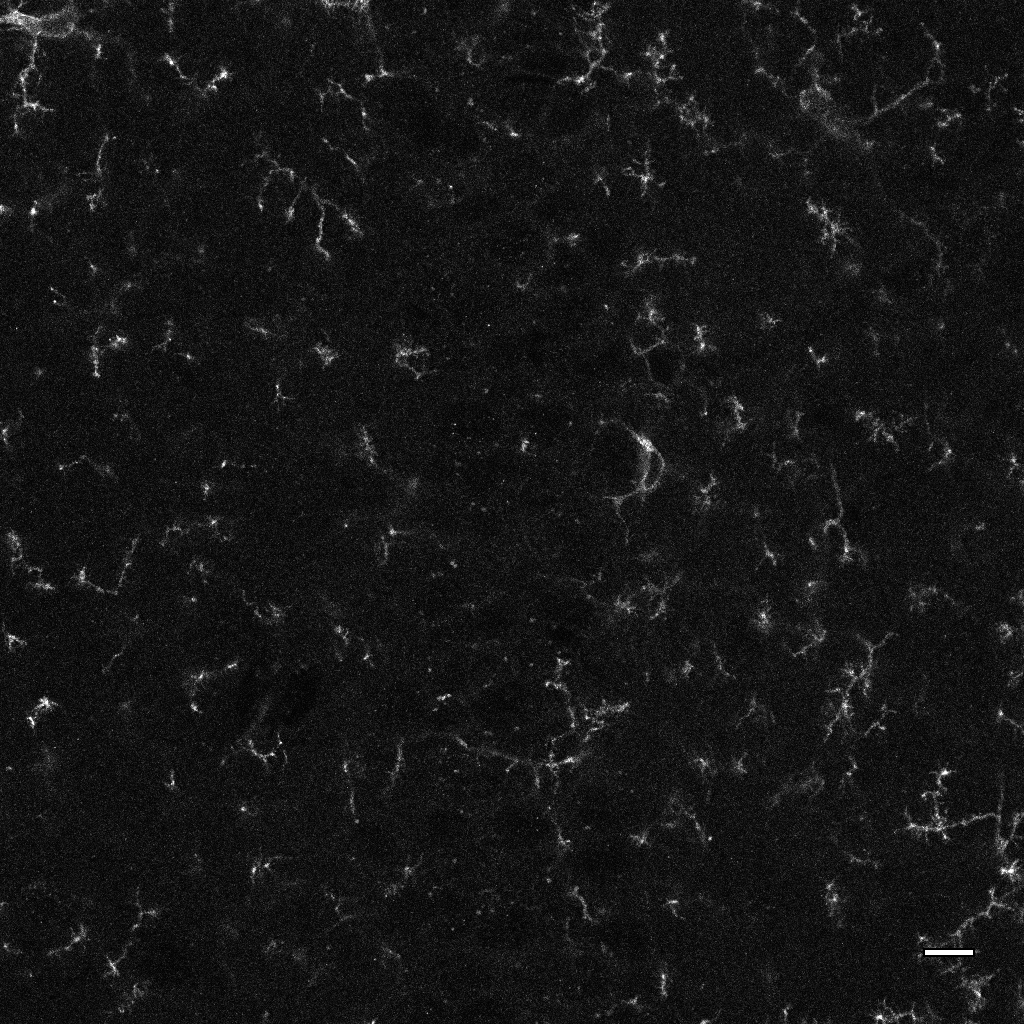

Supplement: Supplementary file 9 — Source Data for Figure 6 [file EMMM-12-e11776-s007.zip › Fig 6 source data 1 September/01kohipp.tif]

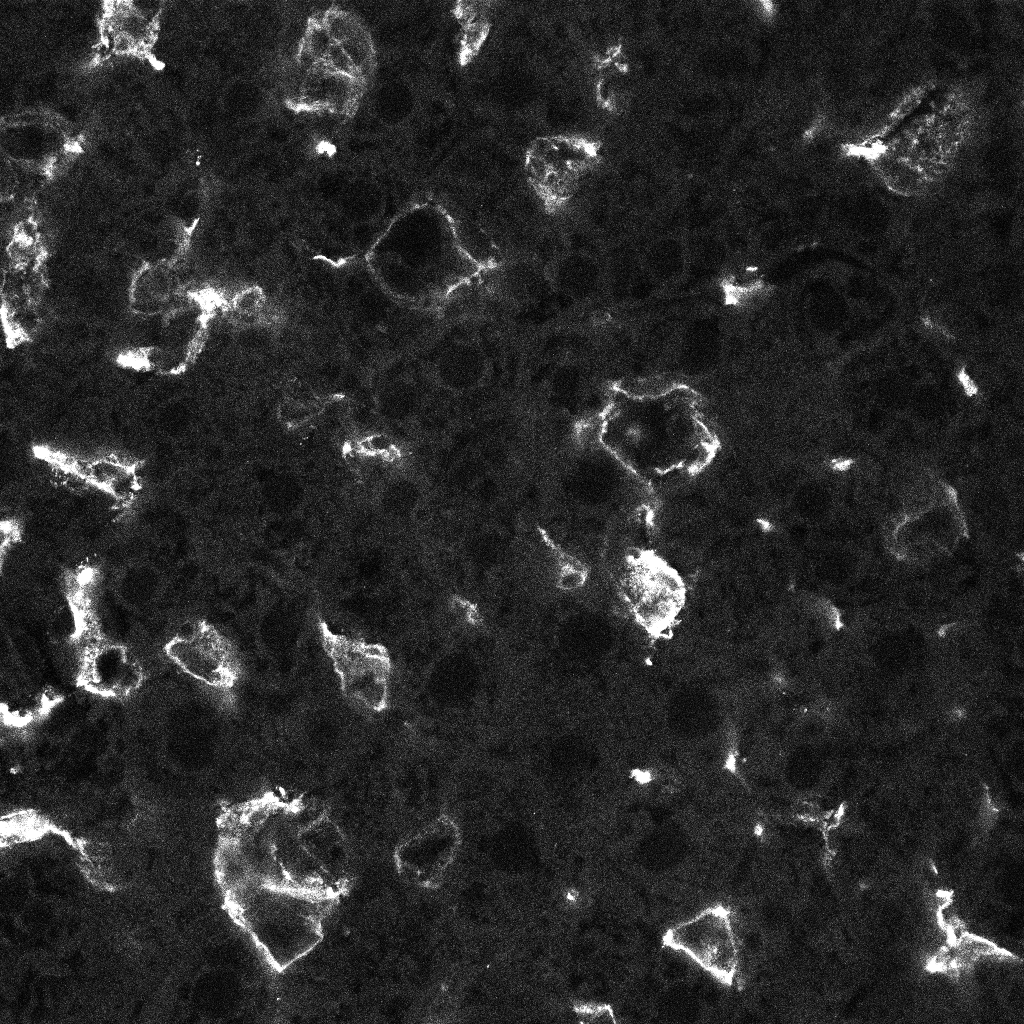

Supplement: Supplementary file 9 — Source Data for Figure 6 [file EMMM-12-e11776-s007.zip › Fig 6 source data 1 September/01Koliv.tif]

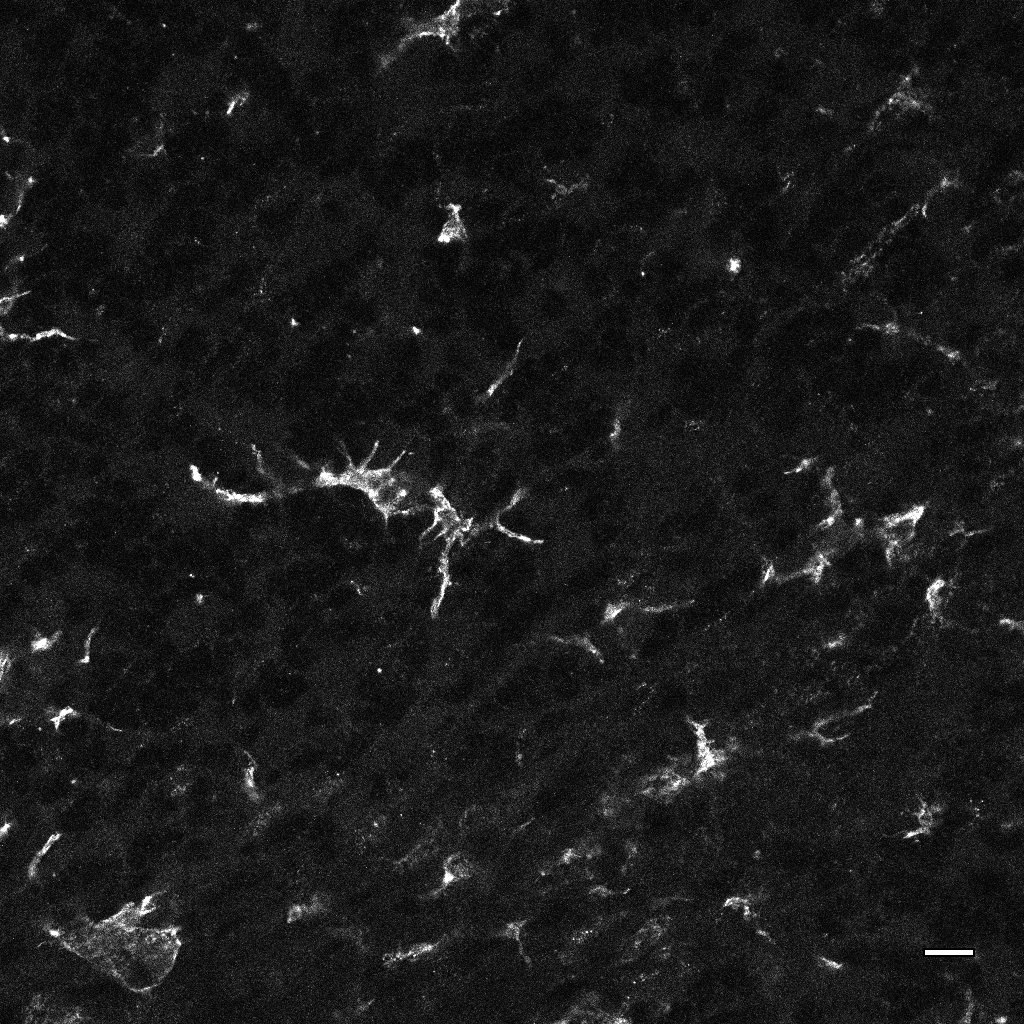

Supplement: Supplementary file 9 — Source Data for Figure 6 [file EMMM-12-e11776-s007.zip › Fig 6 source data 1 September/1kocer.tif]

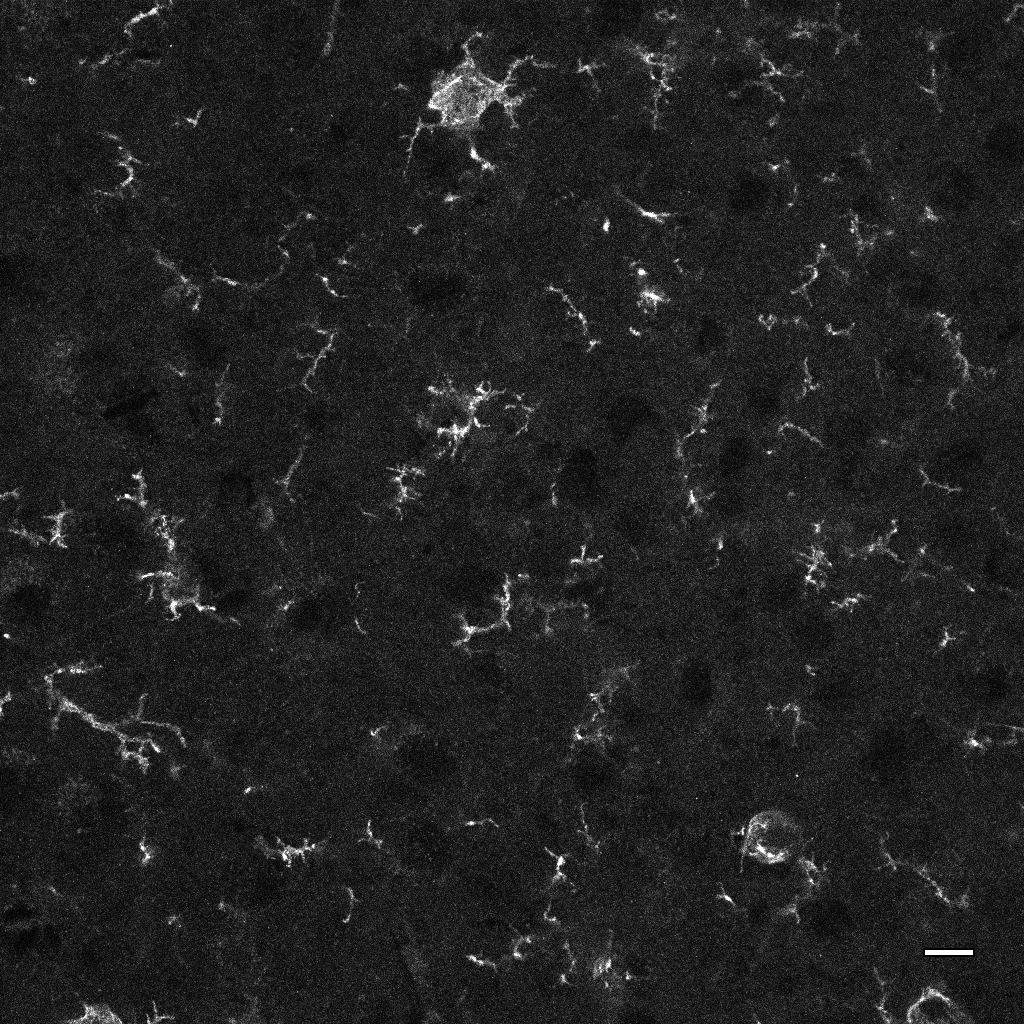

Supplement: Supplementary file 9 — Source Data for Figure 6 [file EMMM-12-e11776-s007.zip › Fig 6 source data 1 September/1kocx.tif]

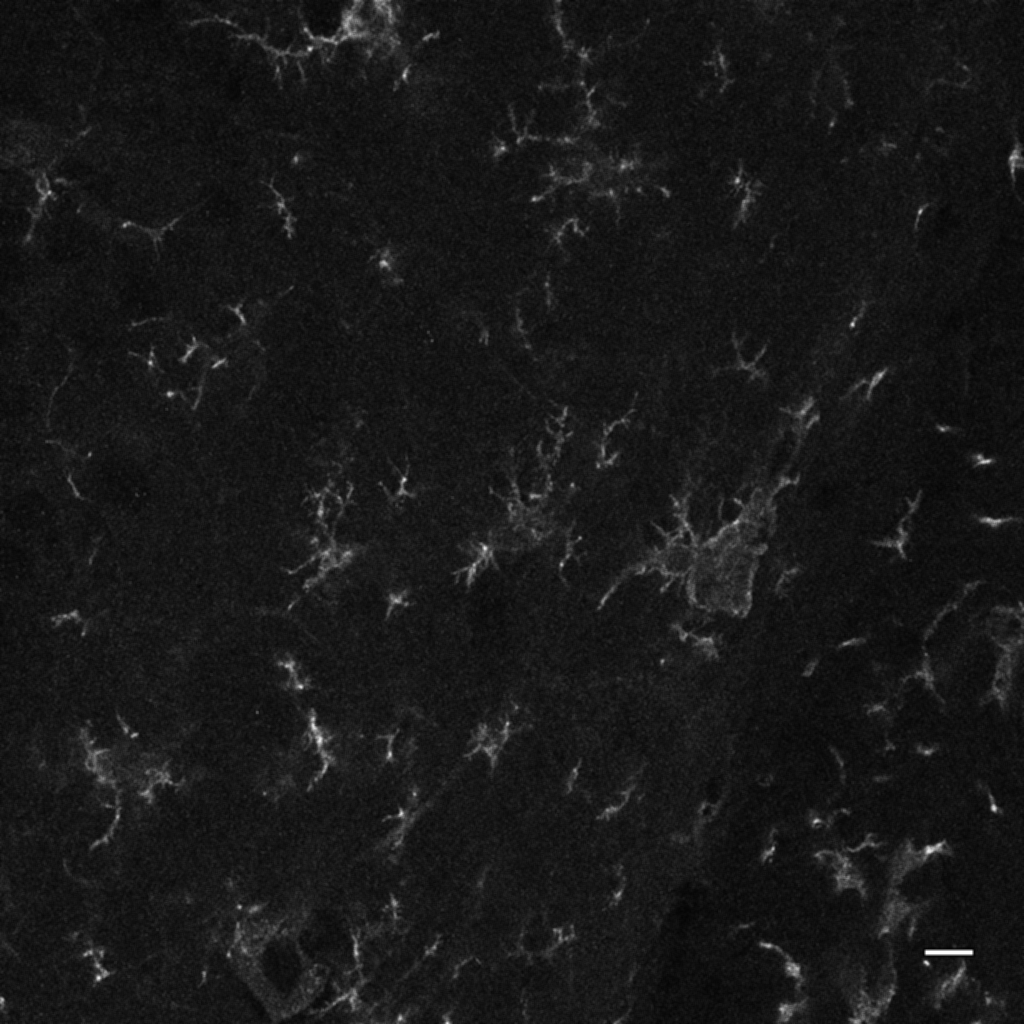

Supplement: Supplementary file 9 — Source Data for Figure 6 [file EMMM-12-e11776-s007.zip › Fig 6 source data 1 September/1kohipp.tif]

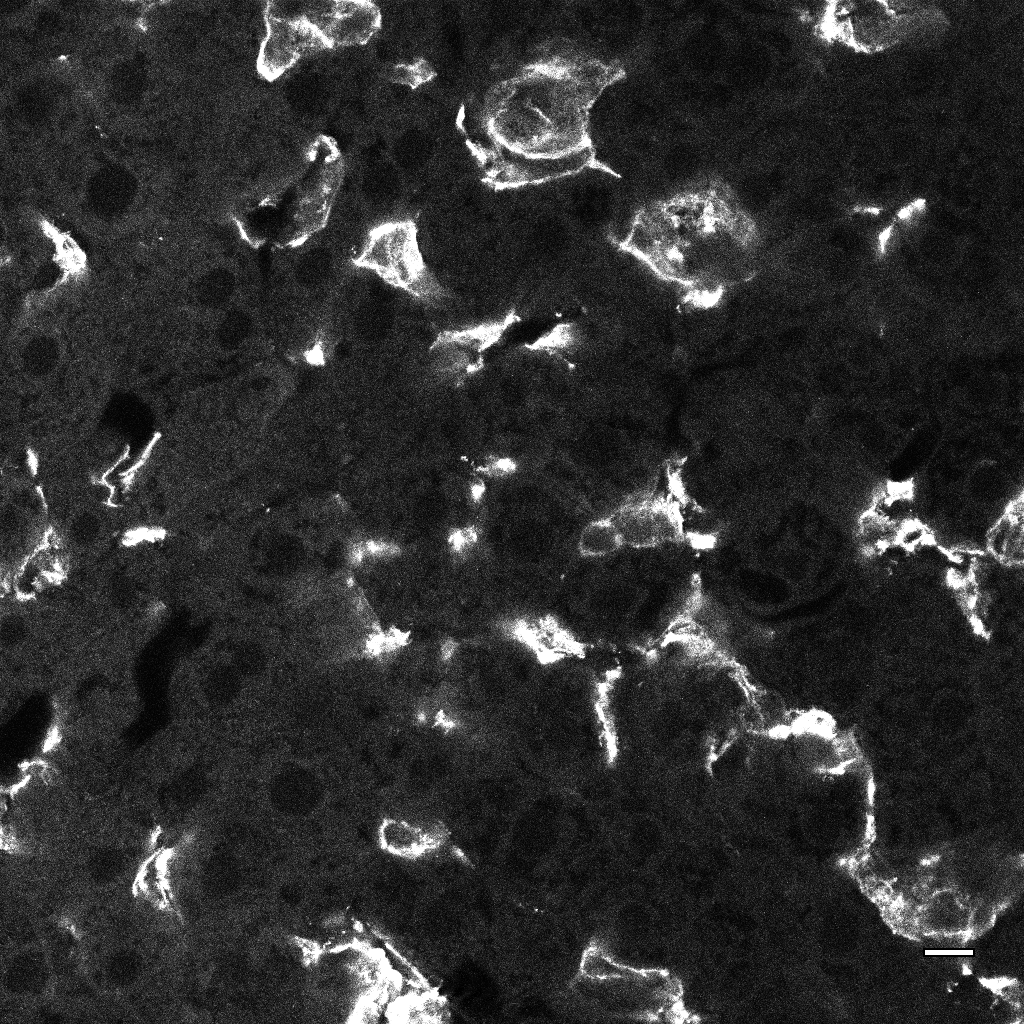

Supplement: Supplementary file 9 — Source Data for Figure 6 [file EMMM-12-e11776-s007.zip › Fig 6 source data 1 September/1koliv.tif]

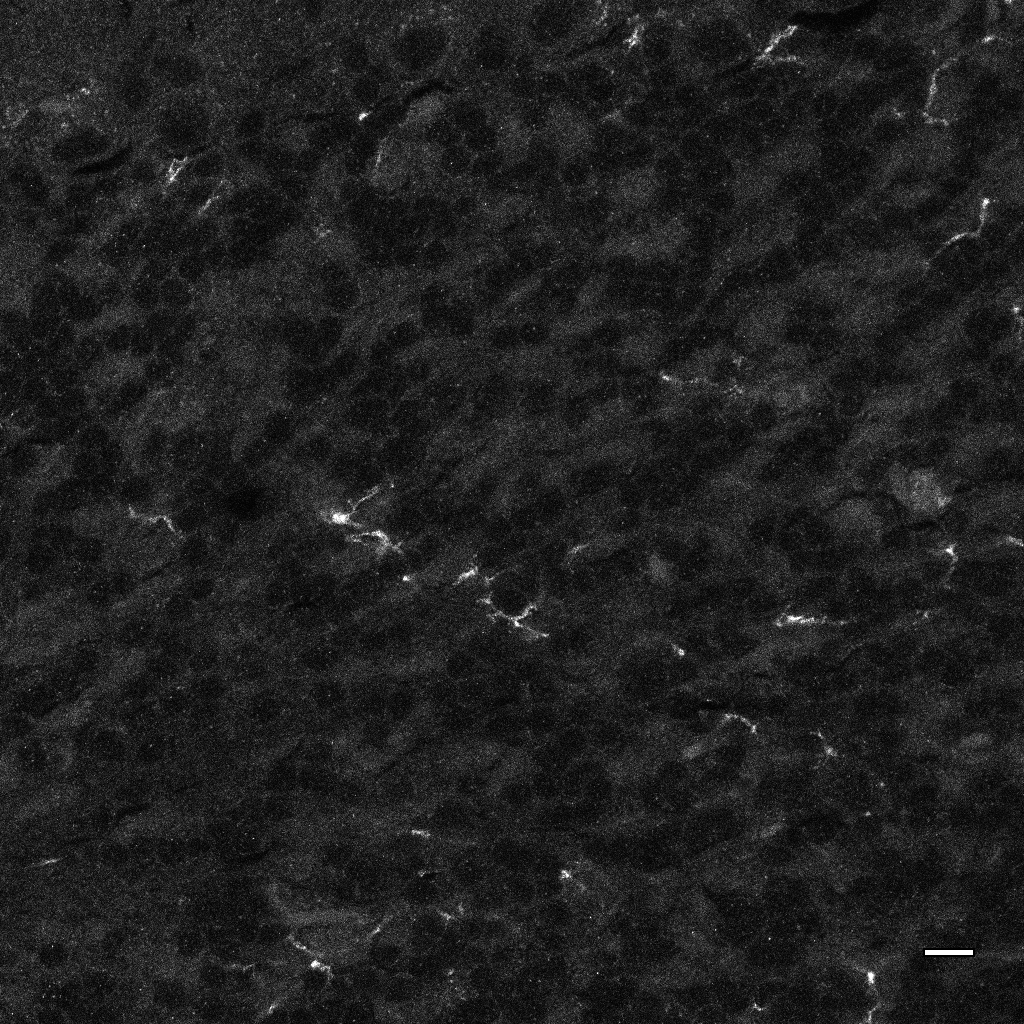

Supplement: Supplementary file 9 — Source Data for Figure 6 [file EMMM-12-e11776-s007.zip › Fig 6 source data 1 September/5kocer.tif]

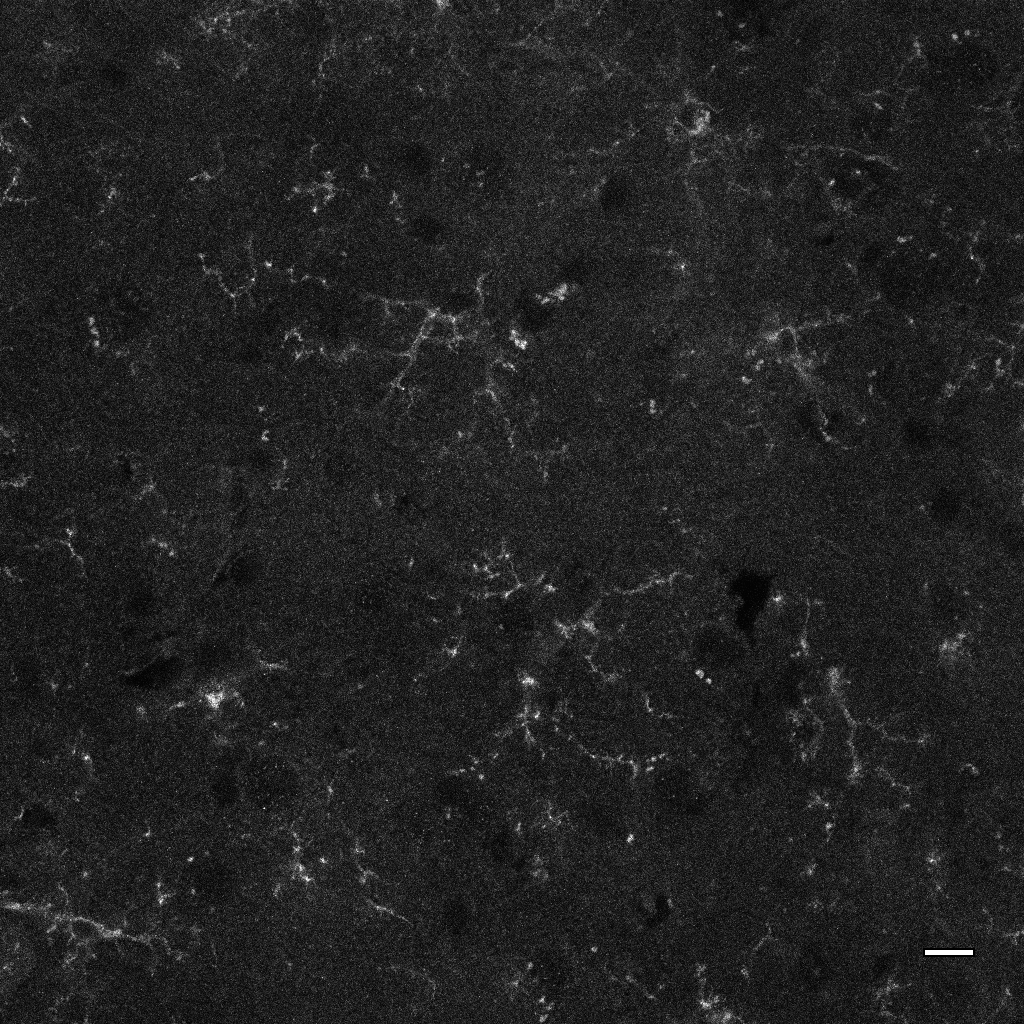

Supplement: Supplementary file 9 — Source Data for Figure 6 [file EMMM-12-e11776-s007.zip › Fig 6 source data 1 September/5kocx.tif]

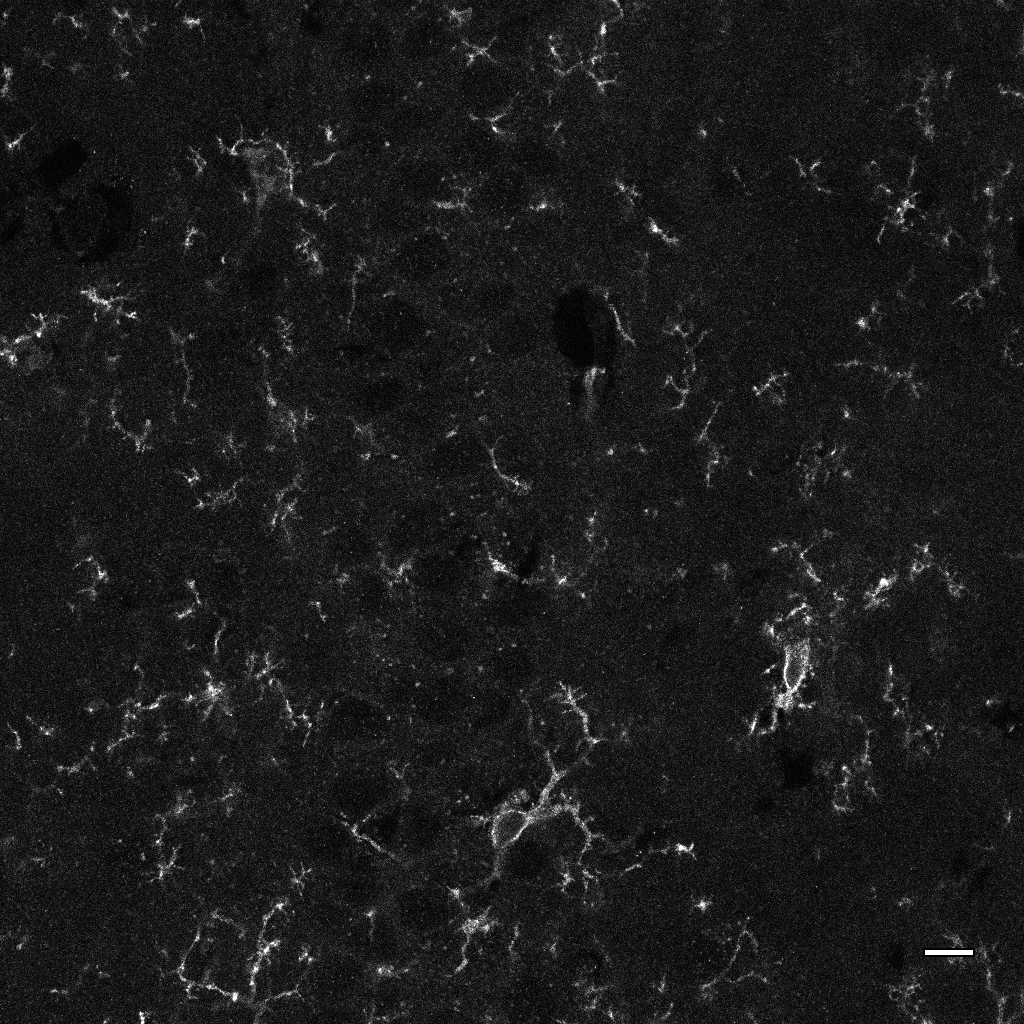

Supplement: Supplementary file 9 — Source Data for Figure 6 [file EMMM-12-e11776-s007.zip › Fig 6 source data 1 September/5kohipp.tif]

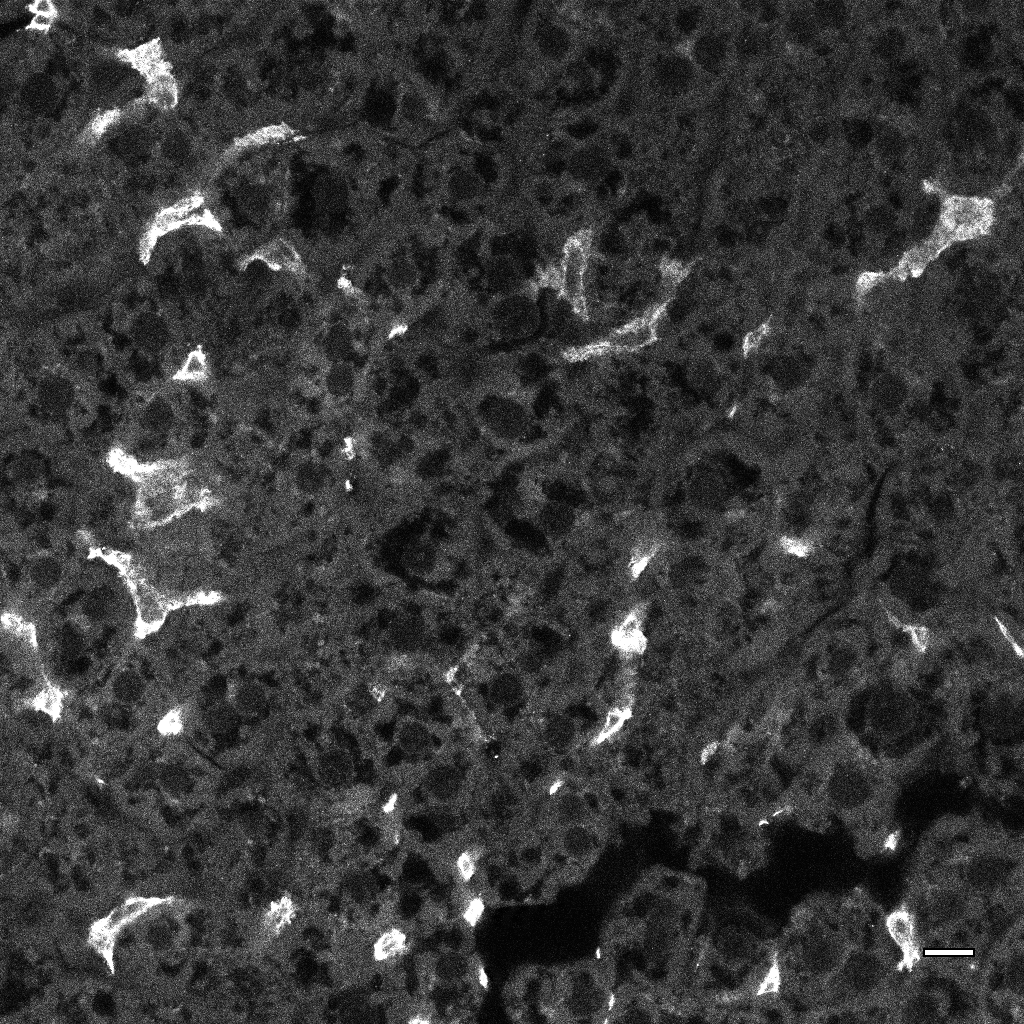

Supplement: Supplementary file 9 — Source Data for Figure 6 [file EMMM-12-e11776-s007.zip › Fig 6 source data 1 September/5koliv.tif]

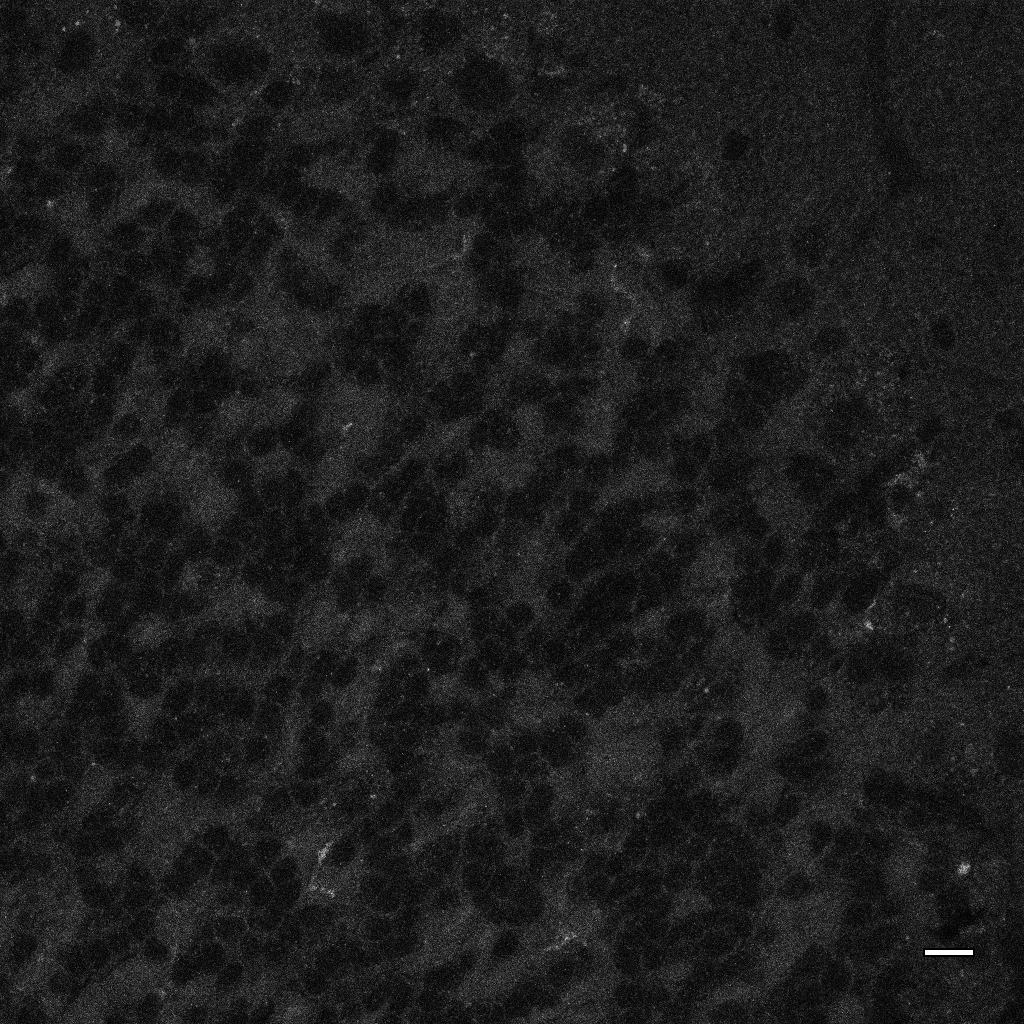

Supplement: Supplementary file 9 — Source Data for Figure 6 [file EMMM-12-e11776-s007.zip › Fig 6 source data 1 September/5wtcer.tif]

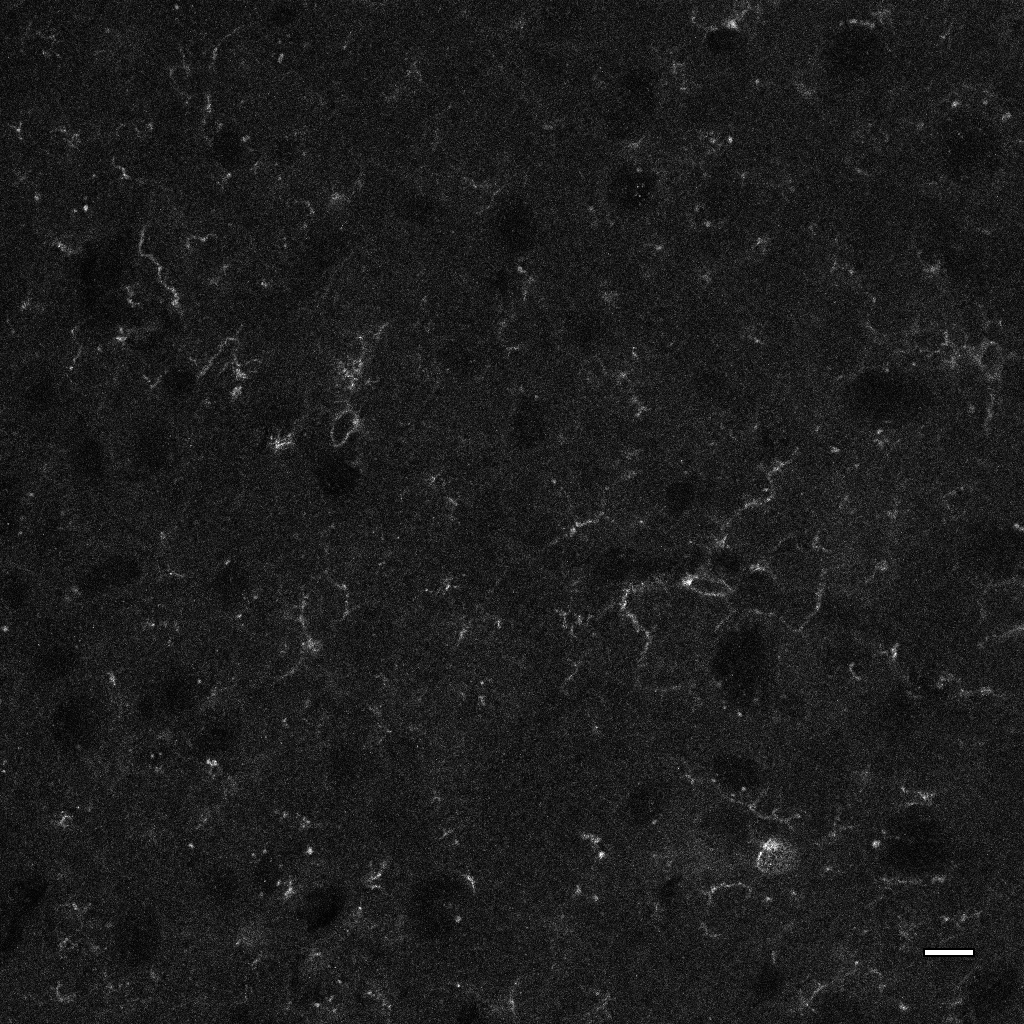

Supplement: Supplementary file 9 — Source Data for Figure 6 [file EMMM-12-e11776-s007.zip › Fig 6 source data 1 September/5wtcx.tif]

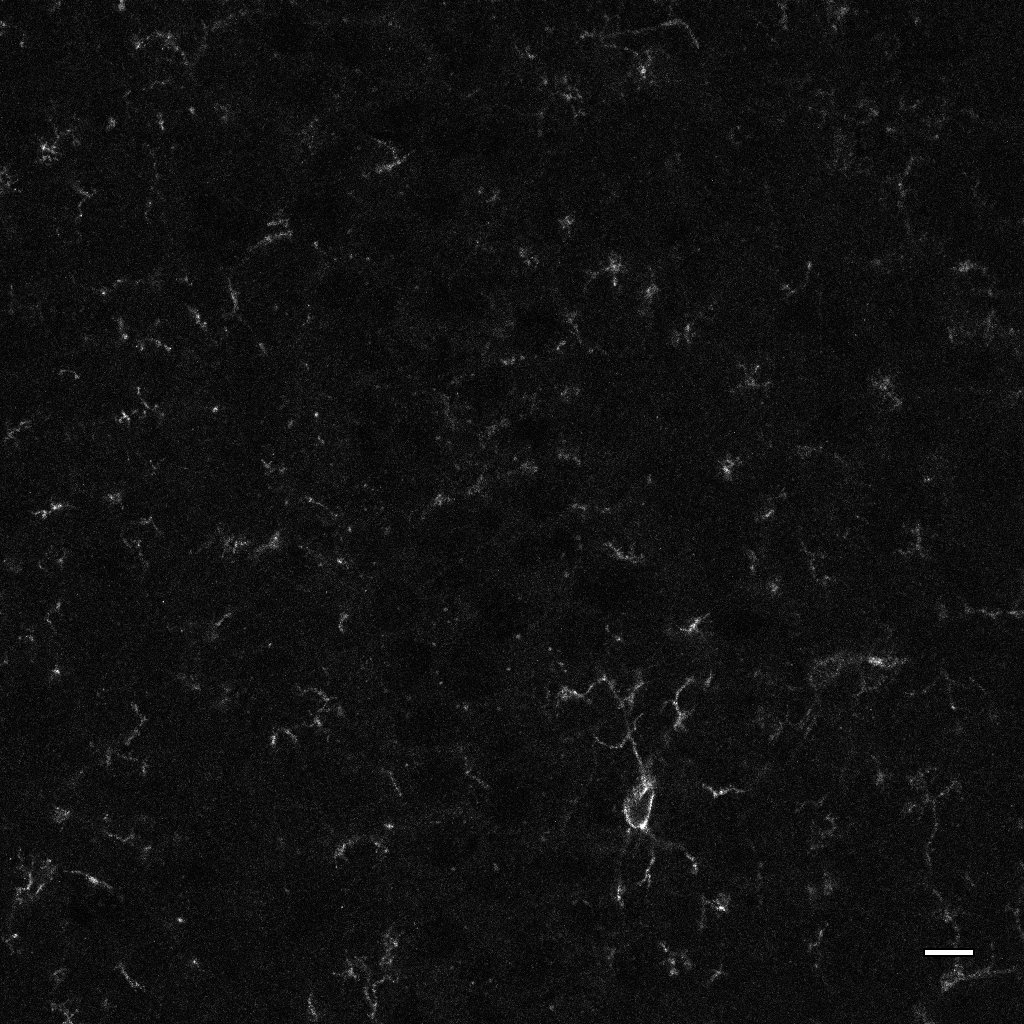

Supplement: Supplementary file 9 — Source Data for Figure 6 [file EMMM-12-e11776-s007.zip › Fig 6 source data 1 September/5wthipp.tif]

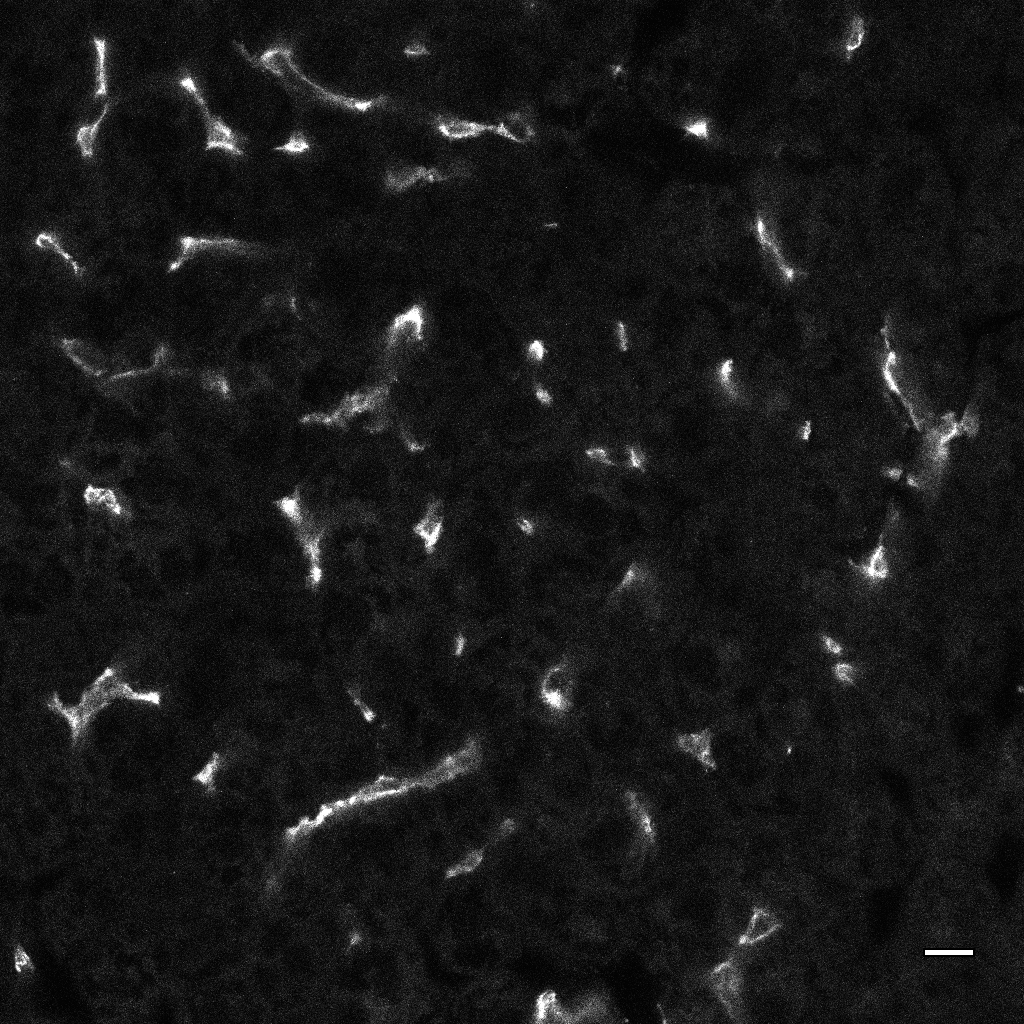

Supplement: Supplementary file 9 — Source Data for Figure 6 [file EMMM-12-e11776-s007.zip › Fig 6 source data 1 September/5wtliv.tif]

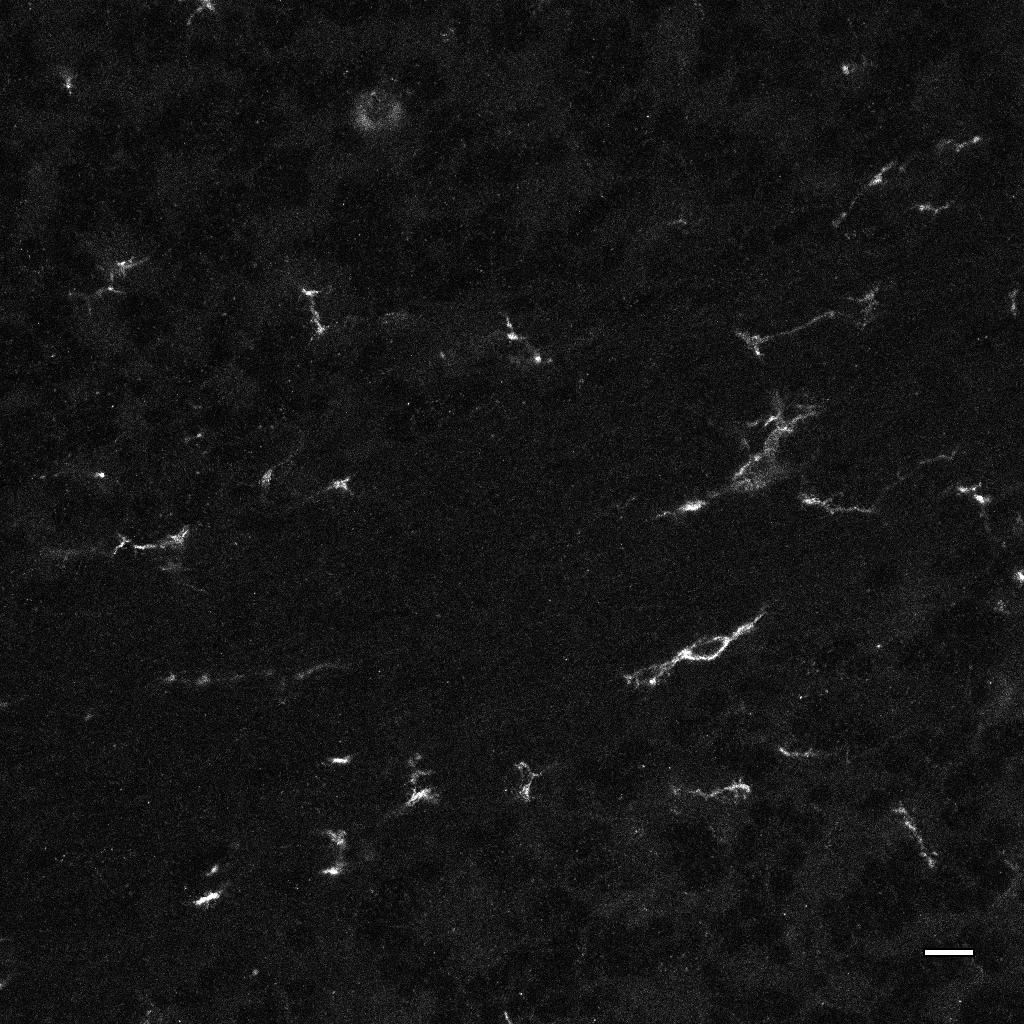

Supplement: Supplementary file 9 — Source Data for Figure 6 [file EMMM-12-e11776-s007.zip › Fig 6 source data 1 September/vehkocer.tif]

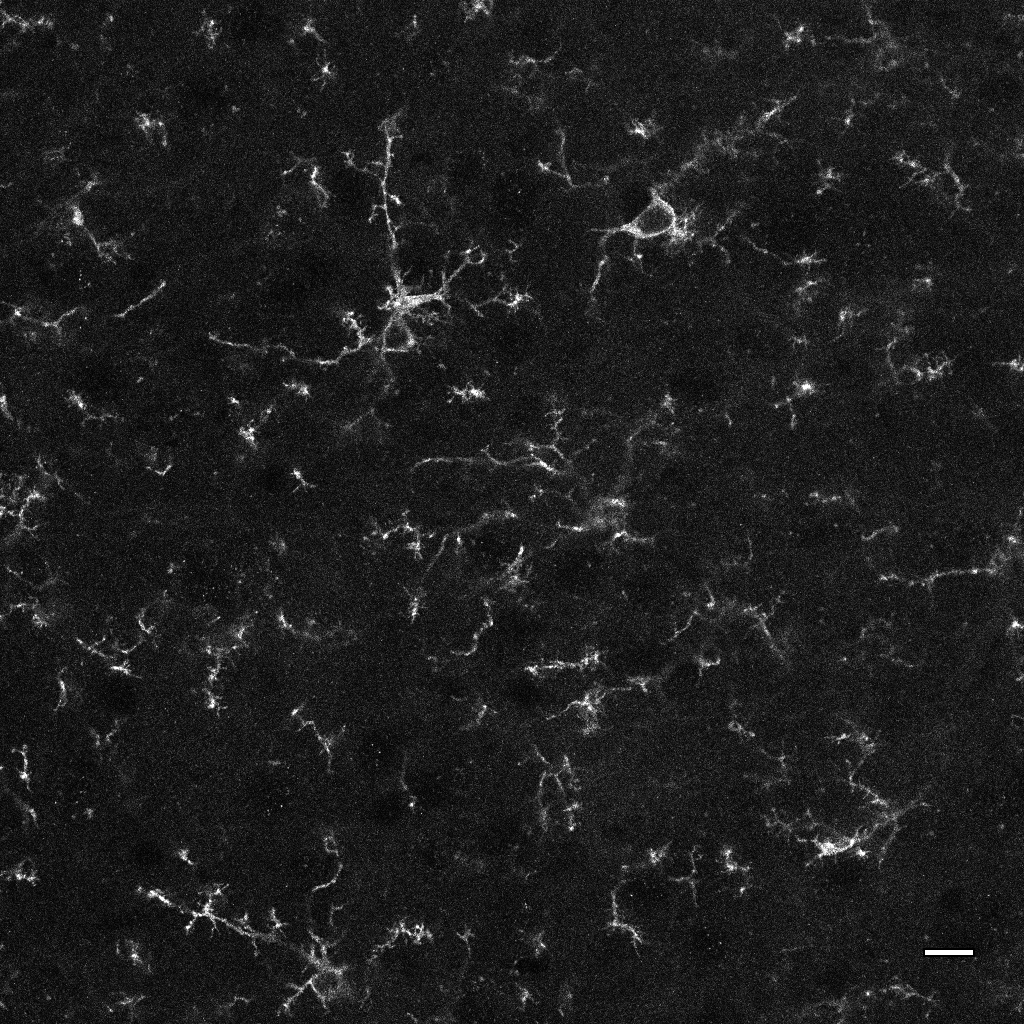

Supplement: Supplementary file 9 — Source Data for Figure 6 [file EMMM-12-e11776-s007.zip › Fig 6 source data 1 September/VehKOcx.tif]

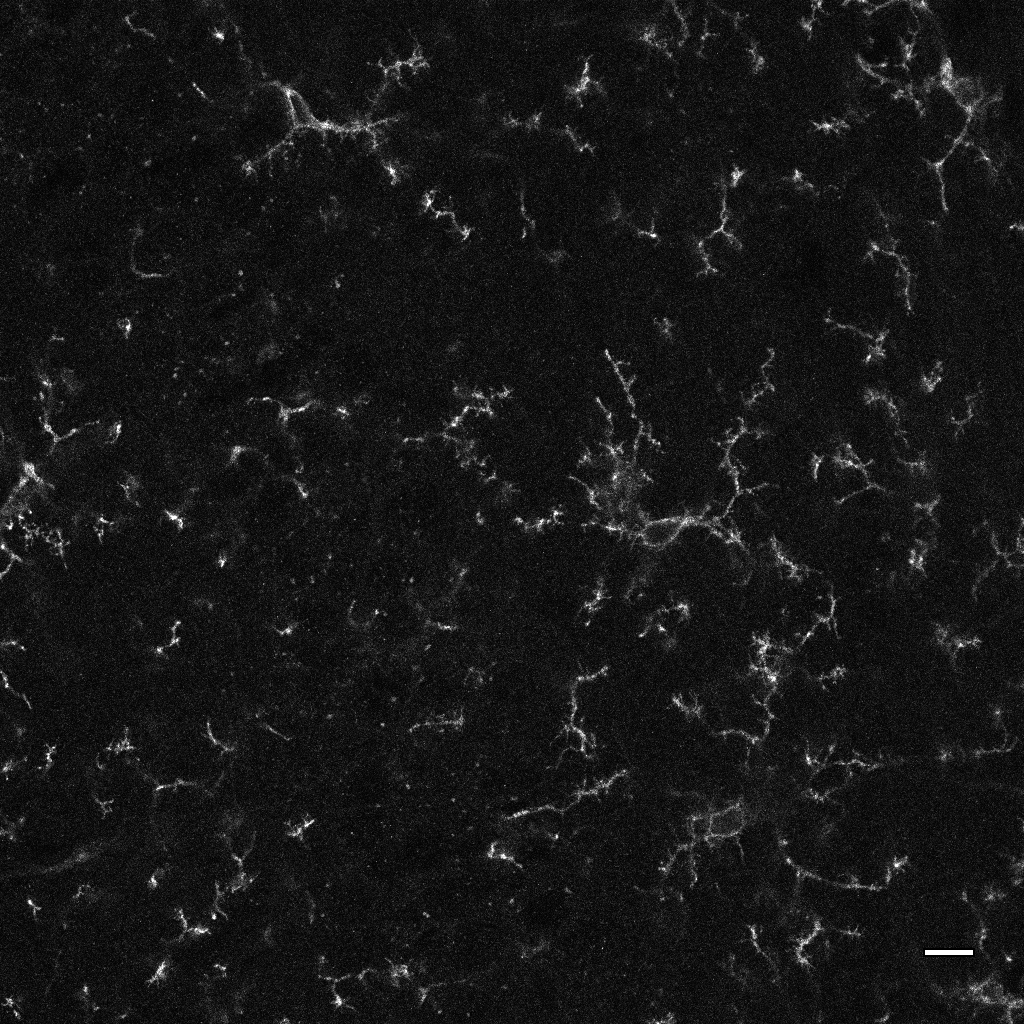

Supplement: Supplementary file 9 — Source Data for Figure 6 [file EMMM-12-e11776-s007.zip › Fig 6 source data 1 September/vehkohipp.tif]

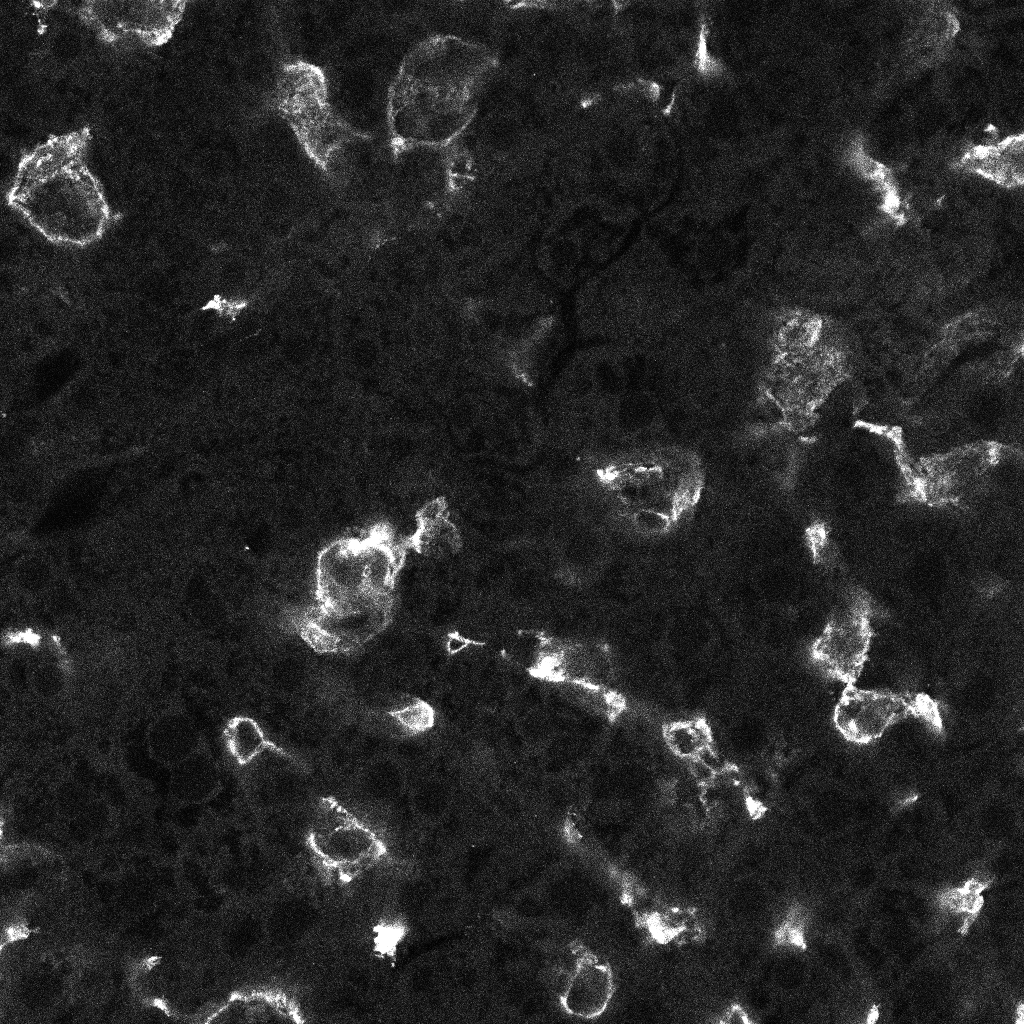

Supplement: Supplementary file 9 — Source Data for Figure 6 [file EMMM-12-e11776-s007.zip › Fig 6 source data 1 September/VehKoliv.tif]

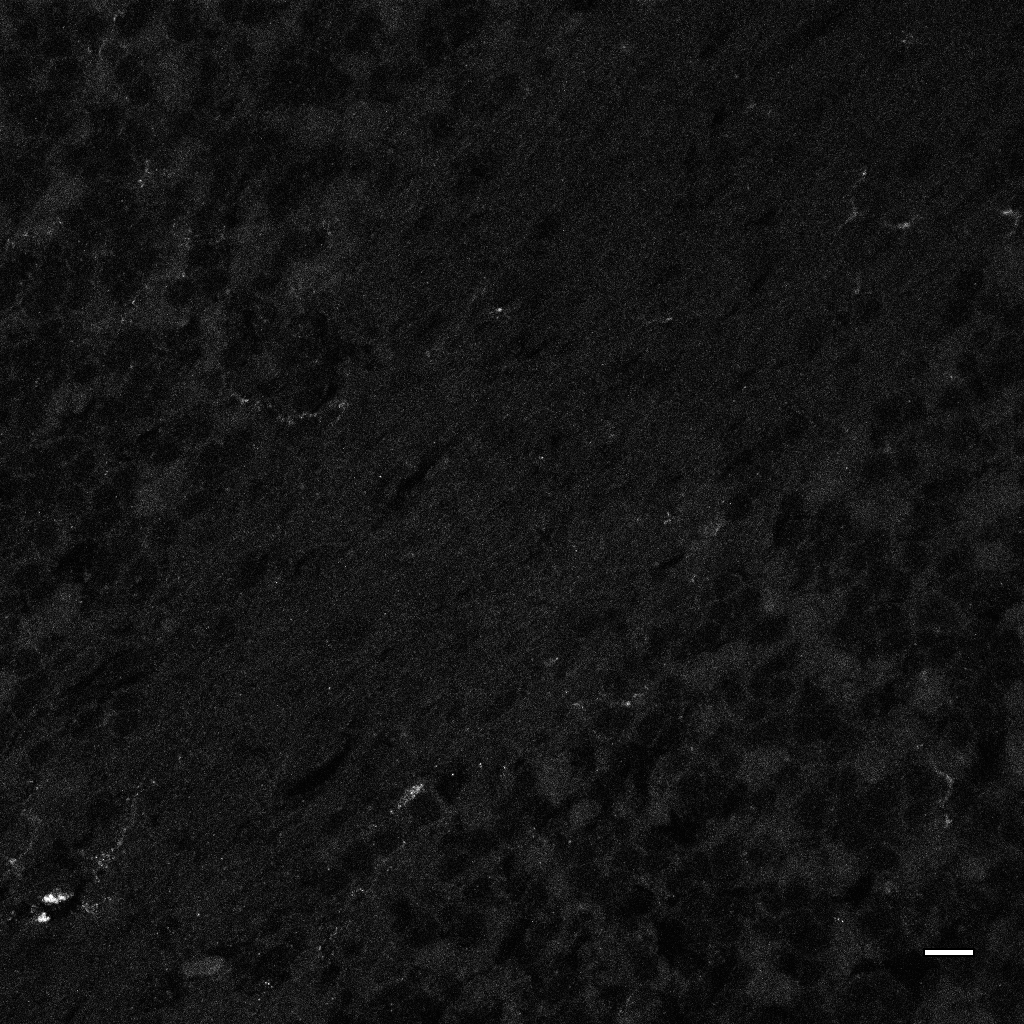

Supplement: Supplementary file 9 — Source Data for Figure 6 [file EMMM-12-e11776-s007.zip › Fig 6 source data 1 September/vehwtcer.tif]

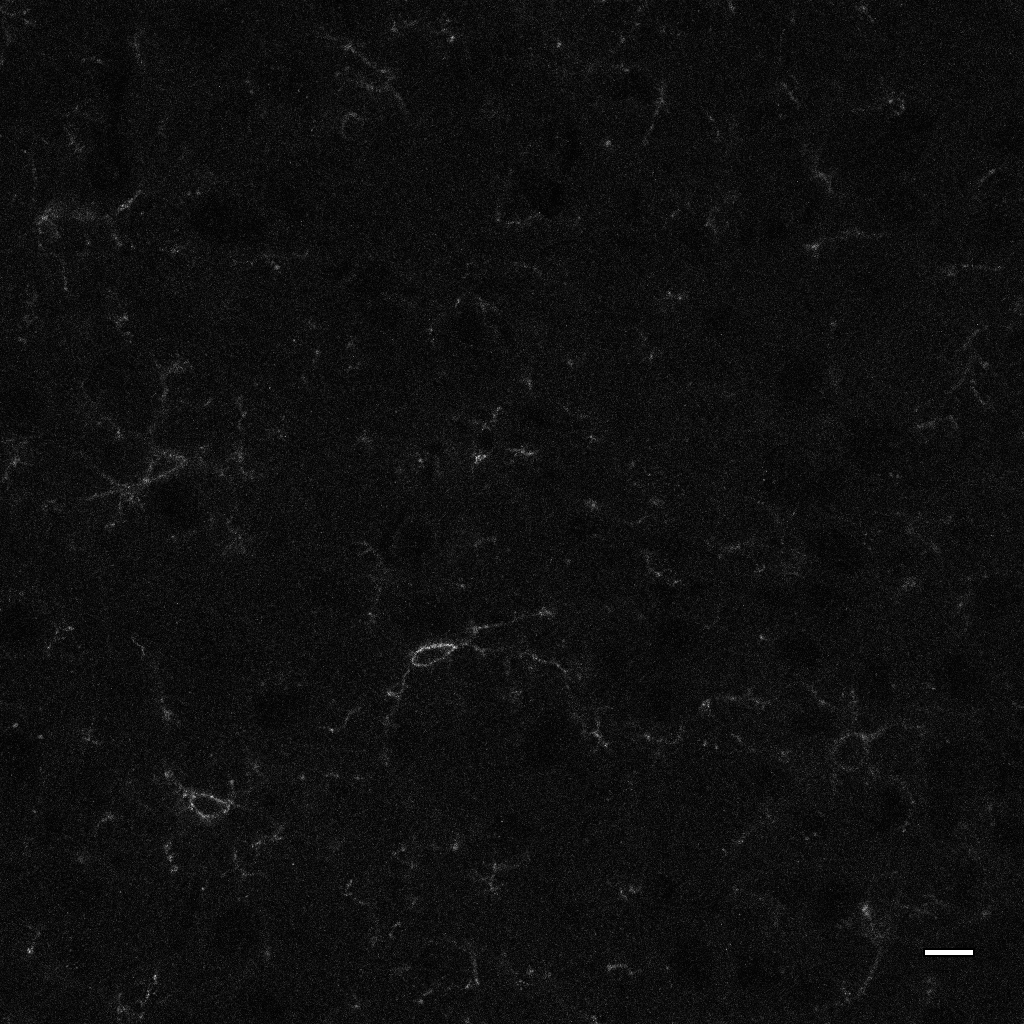

Supplement: Supplementary file 9 — Source Data for Figure 6 [file EMMM-12-e11776-s007.zip › Fig 6 source data 1 September/vehwtcx.tif]

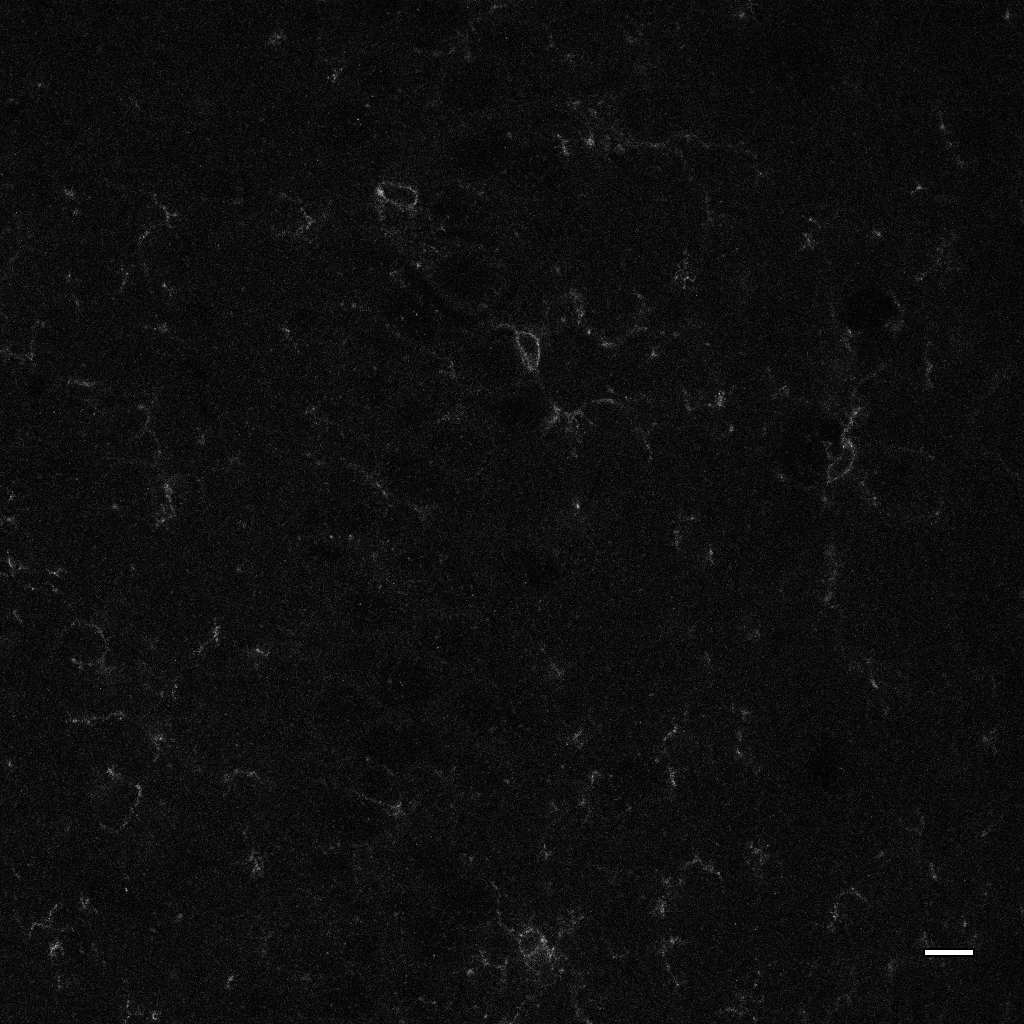

Supplement: Supplementary file 9 — Source Data for Figure 6 [file EMMM-12-e11776-s007.zip › Fig 6 source data 1 September/vehwthipp.tif]

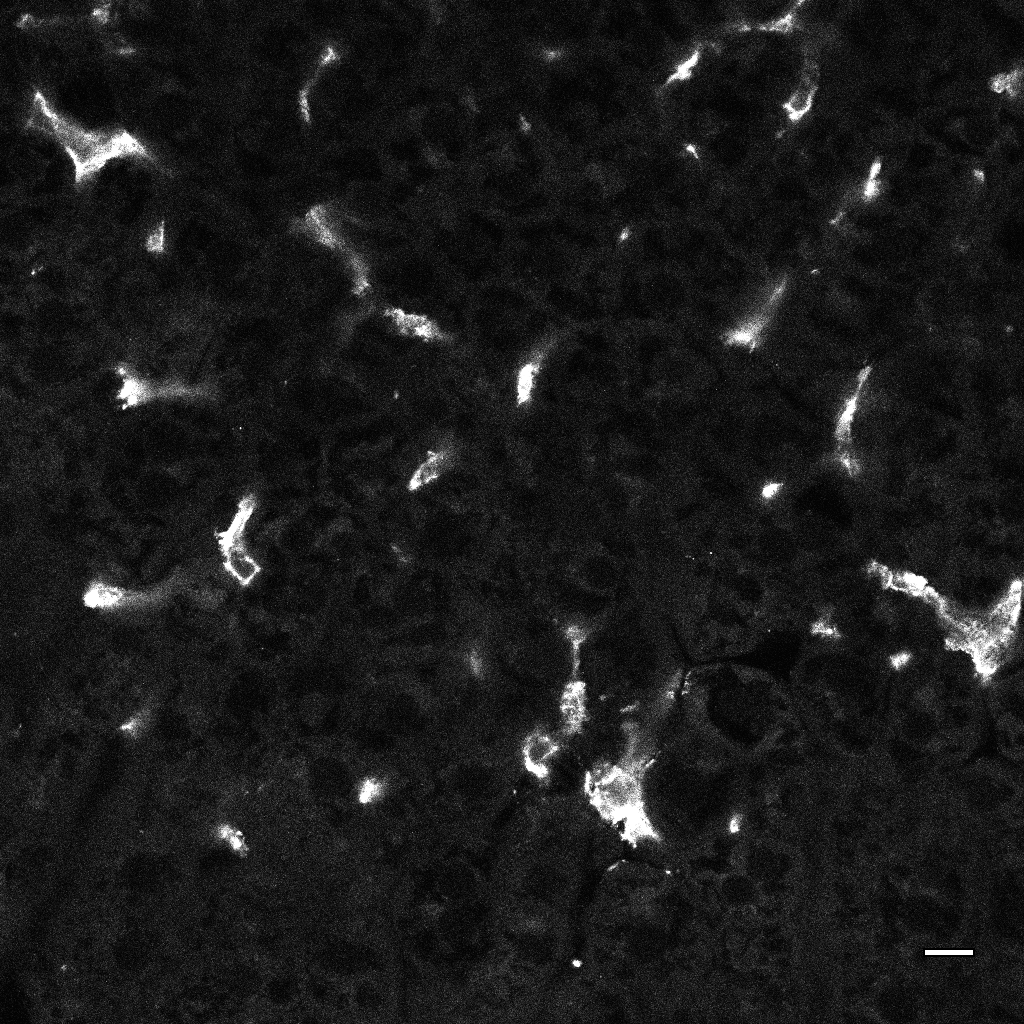

Supplement: Supplementary file 9 — Source Data for Figure 6 [file EMMM-12-e11776-s007.zip › Fig 6 source data 1 September/vehwtliv.tif]
