# Supplementary material for: Inhibition of fatty acid amide hydrolase prevents pathology in neurovisceral acid sphingomyelinase deficiency by rescuing defective endocannabinoid signaling
Source: EMBO Mol Med. 2020 Oct 5;12(11):e11776. doi: 10.15252/emmm.201911776 (PMC7645369; doi:10.15252/emmm.201911776)
Supplement: Supplementary file 3 — Revieew Process File [file EMMM-12-e11776-s010.pdf]

EMBO Molecular Medicine

Inhibition of FAAH Prevents Pathology in ASMD by Rescuing Endocannabinoid Signaling

Maria Dolores Ledesma, Adrian Bartoll, Ana Toledano-Zaragoza, Josefina Casas, Manuel Guzmán, and Edward Schuchman
DOI: 10.15252/emmm.201911776

Corresponding authors: Maria Dolores Ledesma (dledesma@cbm.csic.es)

Review Timeline:

Submission Date:	18th Nov 19
Editorial Decision:	13th Dec 19
Revision Received:	18th Jul 20
Editorial Decision:	1st Aug 20
Revision Received:	12th Aug 20
Editorial Decision:	20th Aug 20
Revision Received:	1st Sep 20
Accepted:	14th Sep 20

Editor: Celine Carret

Transaction Report:

(Note: With the exception of the correction of typographical or spelling errors that could be a source of ambiguity, letters and reports are not edited. Depending on transfer agreements, referee reports obtained elsewhere may or may not be included in this compilation. Referee reports are anonymous unless the Referee chooses to sign their reports.)

13th Dec 2019

Dear Dr. Ledesma,

Thank you for the submission of your manuscript to EMBO Molecular Medicine. We have now heard back from the two referees whom we asked to evaluate your manuscript.

You will see that while they found the study interesting and timely, they also have shared and often overlapping concerns while providing suggestions to strengthen the data and consolidate the conclusions. I won't detail these concerns further as they are clear and straightforward.

We would therefore welcome the submission of a revised version within three to 4 months for further consideration and would like to encourage you to address all the criticisms raised as suggested to improve conclusiveness and clarity. Please note that EMBO Molecular Medicine strongly supports a single round of revision and that, as acceptance or rejection of the manuscript will depend on another round of review, your responses should be as complete as possible.

EMBO Molecular Medicine has a "scooping protection" policy, whereby similar findings that are published by others during review or revision are not a criterion for rejection. Should you decide to submit a revised version, I do ask that you get in touch after three months if you have not completed it, to update us on the status.

Please also contact us as soon as possible if similar work is published elsewhere. If other work is published we may not be able to extend the revision period beyond three months.

Please read below for important editorial formatting and consult our author's guidelines for proper formatting of your revised article for EMBO Molecular Medicine.

I look forward to receiving your revised manuscript.

Yours sincerely,

Celine Carret

Celine Carret, PhD
Senior Editor
EMBO Molecular Medicine

***** Reviewer's comments *****

Referee #1 (Remarks for Author):

The current manuscript by Bartoll and co-workers reveals that endocannabinoid system, in particular CB1 receptor, is downregulated upon sphingomyelin accumulation, as for example observed in the acid sphingomyelinase knockout (ASM-KO) mice. Application of the fatty acid amide hydrolase (FAAH) inhibitor restored CB1 signaling and correlated with the reduced levels of sphingomyelin (SM), reduced inflammation, neurodegeneration and prolonged life span. Data presented in this manuscript suggest that modulating CB1 signaling may provide benefits for treatment of acid sphingomyelinase deficiency (ASMD).
This is an interesting and timely report, as ASMD is a fatal and incurable disease. However, the results presented in the manuscript do not fully support major conclusions and additional lines of evidence should be included. As anticipated by the authors, mechanistic link between CB1 and sphingomyelin has already been established. Although this link has now been explored upon ASAD, the manuscript does not provide additional mechanistic information on how this complex regulatory loop is regulated and whether there are regional or cell-type specific differences that may be of relevance for disease pathology and therapeutic treatment.
Major issues
1) It is very difficult to judge protein levels of CB1 from the presented immunofluorescent images (Fig 1D). This result should be confirmed by Western blot analysis.
2) Immunofluorescent analysis of a single ASMD patient is not conclusive. It is hard to estimate whether the aggregation signal observed in the NPA patient is indeed specific, and if so, this may not be a common pattern. MAP2 signal is very low, even in the control, and almost absent from the NPA patient. Severe neurodegeneration would also interfere with the interpretation of the CB1 levels.
3) The authors suggest that cellular misdistribution of CB1 occurs in ASM-KO neurons and upon sphingomyelin supplementation. It would be beneficial to include lower magnification images showing more than a single neuron. Aggregation phenotype of CB1 is more obvious in Fig 2C and F, compared to Fig 2B and E. Can the cellular misdistribution phenotype of CB1 also be observed in ASM-KO neurons in vivo?
4) Lysosomal accumulation of CB1 has been observed in ASM-KO or upon SM supplementation. The authors discuss that lysosomal degradation may contribute to the reduced levels of CB1 (discussion, page 12). Would inhibition of lysosomal degradation in cultured primary neurons (for example upon addition of SM) effect CB1 levels and its cellular distribution? Along these lines, can normal cellular distribution of CB1 be restored upon treatment of ASM-KO with SMase?
5) Western blot image of CB1 in Fig 2I does not support the reported 45% increase in CB1 protein levels.
6) The authors report a body weight gain in ASM-KO mice treated with PF and claim that no differences were observed in the WT. However, differences can also be observed upon PF application in the WT (Fig 4A). No statistics was included to support the rescue phenotype.
7) Histopathological analysis of the ASM-KO cohort treated longer with PF and included into the survival analysis (Fig 4F) has not been performed and could add additional, therapeutically relevant information when compared with the treatment efficacy over 8 weeks.
8) Figure 5 should be improved to have the analysis more consistent. AEA and NSM levels were

monitored in the hippocampal extracts only (Fig 5A and B). SM analysis was done for the cerebellum, hippocampus and the cortex (hippocampus was indeed less affected). Further immunohistological analysis only revealed changes in the cerebellum. This makes it difficult for a reader to follow the logic. It would be helpful to have AEA, NSM, CB1 and SM levels plus histological examination for the same brain region (or at least the one affected most). Showing only Lamp1 staining in Fig 5D is not beneficial. Cellular marker or at least dapi images should be included to judge what the authors aim to present. Similar to comment #3, also in Fig 5F and G it would be beneficial to have a larger overview to better judge the GFAP and Iba1 pathology.
9) Is the treatment with PF able to correct the cellular distribution phenotype of CB1?
10) Acute treatment is not offering any additional information beyond toxicity assessment. SM levels were only slightly reduced in the cortex. However, the neuroinflammatory effect was also seen in the hippocampus where no SM reduction could be detected. The authors should consider if the statement on page 11 "...and also revealed efficacy after a single administration of high PF doses." is fully supported by the data.
11) I appreciate the efforts towards examining CB1 signaling in other diseases where SM accumulates. However, if the authors wish to study CB1 signaling in NPC, more evidence should be provided, including Western blot analysis of CB1 across different brain regions and their corresponding SM levels. Moreover, brain material and cultured cells from more than 1 NPC patient should be included to make this result conclusive. Also in Fig 7 more consistency would be helpful for a reader. Fig 7A shows CB1 levels in the cerebellum of an NPC1-deficient mouse model, human tissue reveals CB1 staining of a hippocampus and cultured cells of an NPC patient treated with PF were only examined for the levels of SM (CB1 analysis is missing). Analysis of other phenotypes such as cellular distribution of CB1, lysosomal morphology or levels of cholesterol upon PF treatment would provide additional information to judge the therapeutic potential of CB1 modulation in NPC disease.
Minor issues
1) Authors should discuss differences between CB1 (neuronal) and CB2 (immune cells) expression patterns that may result in different signaling networks in neurons vs microglia. Potential common (or diverse) mechanism of FAAHi effect in different nervous system cells could be discussed.
2) Authors should consider improving figure legends such as in Fig 4A and F to distinguish more easily between different conditions.
3) Consider re-phrasing "...dual impact" on page 11 of the discussion.
4) Reference Nr 50 on page 15 of the discussion should be corrected according to the common reference style.

Referee #3 (Remarks for Author):

The authors describe the importance of the eCB system in the development of severe neurological disorders like ASMD and propose a new therapeutic strategy based on the modulation of eCB system by acting on the CB1 receptor for rescuing the pathological storage of SM, inflammation and behavioral abnormalities in the ASM-KO mouse model.
The invitro study performed by using cultured hippocampal neuronal cells represents an optimal method to validate the role of CB1 receptor and its direct link with the SM accumulation.
Starting from this invitro validation, the authors tried first to confirm in ASM-KO mice the role of CB1 receptors in the brain pathology development and after to test the efficacy of a therapeutic approach based on the use of FAAH inhibitors for rescuing the neuropathology in this animal model and also in NPC human cell line.
While the study is of potential interest some data should be improved to better validate the

effectiveness of this strategy for the treatment of neuropathology in ASMD mouse model.

Major Comments:
-Figure 1 and Results: The fluorescence intensity measurement doesn't represent a quantitative analysis of CB1 protein. The authors should perform the quantitative analysis of CB1 by WB experiments in the same brain regions used for qPCR experiments.

-Results page 5: Since the CB1 receptors are well abundant in the prefrontal cortex, it should be interesting if the authors indicate in text and in the figure legend of Fig.1C which cortical regions have been analyzed and if they performed all the experiments in the same mouse cortical regions.

-Figure 1D: the author should improve the resolution of cerebellum images in which it is difficult to appreciate the signal of CB1 receptor.

-Figure 1E: In the IF staining on cerebellum, the MAP2 signal is almost undetectable in the slides of both CTR and NPA patients and it is also complicated to understand the morphology of this brain region.
The author should improve the IF experiments in order to better analyze the decrease of CB1 signal in the cerebellum of NPA patients. They could also try to perform a labeling with Calbindin/CB1/TOPRO markers.

Figure 2
-In vitro study:

Figure 2A: In order to quantify the expression levels of CB1 receptor, the authors should perform the WB analysis on WT and ASM-KO hippocampal cells. The immunofluorescence experiments represent only a semi-quantitative analysis able to support the WB data.
Moreover, since the authors explain in the results that the main pathological hallmark of the ASMD is represented by the increase of SM levels, it is important to quantify the amount of sphingomyelin in both WT and ASM-KO neuronal cells.

-Figure 2C: Since the measure of CB1 fluorescent intensity has been already quantified in the figure 2B, the authors should replace the CB1 quantification graph in Fig 2C with another one in which they show the LAMP1 quantification as marker of autophagy impairment.
Moreover, the authors showed an increase of CB1 in the cell body of ASM-KO cells. Since this increase is associated with an increase of SM levels, the author should show a quantification of SM levels among WT and ASM-KO cells.

-Figure 2D-2E: The authors could show the qPCR and the IF experiments in WT and also ASM-KO cell lines. Furthermore, they should perform the WB analysis for CB1 receptor with the relative protein quantifications in the following neuronal cell lines: WT, WT+40uM SM and ASM-KO.

-Figure 2F: Since the authors quantified in both panels 2E and 2F the CB1 signal using the same cell lines, they should replace the figure 2E with figure 2F.

-Figure 2G, 2H, 2I: The authors should also perform the experiments in the WT cell lines as positive control.

-Figure 3A-3E: the authors should also perform the experiments in the WT neuronal cell line as CTR cell line.

-Page 7: The authors demonstrated that FAAH inhibitors act with the participation of CB1 receptors by an indirect experiment in which they measured the SM levels in ASM-KO cells treated with PF-04457845 and with SR+PF-04457845.
The authors should also evaluate the expression levels of the CB1 receptor by WB experiments on ASM-KO cells treated with PF-04457845 and SR+PF-04457845.

Figure 3C: The authors should directly quantify of CB1 receptor levels by WB experiments in WT and ASM-KO cells treated with AEA with or without the GW4869.

Figure 3F: In order to both evaluate the neuronal morphology and CB1 neuronal distribution, the authors should perform a Co-IF experiments with MAP2 and CB1 receptor markers on ASM-KO treated cells and WT cells. The authors should also quantify the levels of CB1 in ASM-KO cells by WB analysis upon the treatment with FAAH inhibitors.

Figure 5B: The authors quantified the NSM protein in hippocampal extracts from WT and KO mice. They should provide an image of the entire WB filter with all experimental groups of mice. Moreover, in order to quantify the levels of CB1 receptor they should perform a WB analysis of this protein in hippocampal extracts of treated mice.

Page 9: the authors performed the molecular and biochemical analyses on hippocampal, cerebellum and cortex extracts of ASAM-KO treated mice. They could provide immunofluorescence experiments for LAMP1,IBA1 and GFAP markers also in the hippocampus of KO treated mice.

Figure 5D, 5E: The authors show in the panel D, the LAMP1 staining in cerebellum samples of treated mice.
Since it is difficult to appreciate to LAMP1 localization and the cerebellum morphology, the authors should perform a co-labeling with LAMP1 markers and DAPI.

Moreover, since the authors demonstrated an increase in the LAMP1 and CB1 colocalization in ASM-KO cells, they should also perform a co-labeling experiment with LAMP1 and CB1 markers in cerebellum and hippocampus of treated mice. This data should support the biochemical experiments performed in the same animals.

Figure 5F-5G: the authors show the IBAI and GFAP staining in the cerebellum of treated mice. It is difficult to appreciate the cerebellum morphology, probably because they used a different magnification respect to the previous images. In order to better understand the signal of both markers, the author should provide images with higher resolution and lower magnification. Moreover, they could provide the inflammation analysis (IBA1 and GFAP markers) in the hippocampus of treated mice.

Figure 6A: the authors should perform a tunnel assay in a representative brain region of KO mice treated with higher doses of PF in order to exclude any toxic effect on the neuronal cells.

Figure 6C: the authors performed the IF experiments with IBA marker in order to evaluate the microglia activation in the brain regions of KO mice treated with different concentration of PF. In order to better appreciate the morphology of brain regions and IBA distribution, the authors should perform immunofluorescence experiments in the mouse brain regions with IBA1 marker and DAPI.

Page 11: To understand the role of CB1 in the neuropathology development, the authors should improve the invitro studies in NPC fibroblasts by:
-Quantifying the CB1 receptor in WT and NPC fibroblast cell line from patients with WB experiments.
-Treating the NPC and WT fibroblast cell line with SMase and evaluating the SM and CB1 levels upon the treatment.
-Performing IF analysis for CB1 and LAMP1 markers in order to evaluate the CB1 co-localization with lysosomes and a possible block of autophagy in NPC fibroblast cell line.

Moreover, in order to quantify the levels of CB1 receptors they should perform WB experiments in the brain samples and also Immunofluorescence staining for CB1 receptor in the hippocampus of WT and NPC1 nmf164 mice.
To test the therapeutic effectiveness of the PF treatment in NPC1 nmf164 mouse models, the authors could perform a short term study with the best dosage of PF and analyze the CB1 and SM levels in the brain samples of treated mice.

Minor comments:

FigureS1A: the authors should explain how they calculated the fluorescence intensity in the hippocampus region of treated mice and if they normalized the data respect to the brain areas or to the number of cells analyzed.

-Page 6: In the results it has been described a change in CB1 distribution in the ASM-KO neuronal mouse cell line the. The authors should comment this part in the results and in the discussion.

Figure 6: the authors should indicate which cortex regions they have analyzed.

Figure legend 7A: In the figure 7A and in its figure legend there is a discordance about the nuclei marker used in the experiment. The authors should indicate the nuclei marker.

Figure 7A, 7B: The authors should show immunofluorescence images at low magnification in order to better appreciate the CB1 reduction and distribution in hippocampus NPC1 nmf164 mice.

POINT-BY-POINT ANSWER TO REFEREES

Please find below the Point-by-Point answer to referees in which we detailed the experiments and modifications done in our manuscript EMM-2019-11776. These are highlighted in the main text (underlined and yellow labelling) and in the following new Figures: Figure 1D,E,F,G,H; Figure 2B,D,E,H,J,N; Figure 4A; Figure 5C,D,E,F; Figure 7A,C,D,E,F,G,H and new Appendix Supplementary Figures S1, S2, S3, S4, S5, and S7.

Referee #1

We thank this referee for considering our report interesting and timely. We are grateful for his/her careful evaluation and queries that have been addressed as follows:

Major points
1) It is very difficult to judge protein levels of CB1 from the presented immunofluorescent images (Fig 1D). This result should be confirmed by Western blot analysis.
We have performed Western blot analysis to quantify CB_1 levels in cerebellar, hippocampal and cortical extracts. No significant differences were observed although a tendency to reduction was found in the cerebellum (Figures 1D and S1). This moved us to improve the original immunofluorescence analysis and checked in detail the cell-type specific expression of CB_1 by co-labelling with neuronal, astrocytic and microglia markers in the cerebellum (the most affected area in the disease). CB_1 levels were significantly reduced in the Purkinje cells (identified by calbindin staining) as well as the co-localization of CB_1 with these neurons as indicated by a diminished Mander´s coefficient (Figure 1E). However, CB1 levels were not significantly reduced in astrocytes (identified by GFAP staining) or microglia (identified by F4/80) (Figure 1F,G). While the co-localization studies revealed an unchanged CB_1 level in microglia it was notably increased in astrocytes, probably due to the higher number of these cells in the cerebellum of ASM-KO compared to WT mice (Figure 1F,G). These results, together with the reduced levels of CB1 observed in neurons of the cerebellum and medium bulb in the ASMD patient (Figures 1H and S2) as well as the results in cultured hippocampal neurons from the ASM-KO mice (Figures 2A,B), lead us to conclude that reduction in CB_1 in ASMD mainly affects neuronal cells compared to glia cells. This may explain why we see no significant differences in CB_1 levels when monitored by Western blot in total extracts. This is also discussed on pages 6 and 13 of the revised text.

2) Immunofluorescent analysis of a single ASMD patient is not conclusive. It is hard to estimate whether the aggregation signal observed in the NPA patient is indeed specific, and if so, this may not be a common pattern. MAP2 signal is very low, even in the control, and almost absent from the NPA patient. Severe neurodegeneration would also interfere with the interpretation of the CB1 levels.
We agree with the reviewer that analysis of a single ASMD patient is not conclusive. However, being such a rare disease tissue samples from ASMD patients, especially from the brain, are almost impossible to obtain. We are indeed very grateful to the Wylder Nation Foundation for sharing with us the only brain tissue they had available.

We believe the results from this single individual are worth showing, particularly since information on human patients is so limited in the literature. Nevertheless, to address this concern, in the revised text we no longer highlight the aggregation pattern and now stress that the results in the human samples are not conclusive since they are derived from only one patient (page 6).
To rule out that the decreased CB_1 levels observed in the NPA patient are due to severe neurodegeneration we have performed co-labelling with calbindin (for Purkinje cells of the cerebellum) and with MAP2 (for neurons of the medium bulb) and chose for CB_1 quantification only those neurons showing similar integrity in the control and NPA samples. We include these images and quantification in the Figures 1H and S2, which confirmed the significant reduction of CB_1 levels in neurons of the NPA patient.

3) The authors suggest that cellular misdistribution of CB1 occurs in ASM-KO neurons and upon sphingomyelin supplementation. It would be beneficial to include lower magnification images showing more than a single neuron. Aggregation phenotype of CB1 is more obvious in Fig 2C and F, compared to Fig 2B and E. Can the cellular misdistribution phenotype of CB1 also be observed in ASM-KO neurons in vivo?
We agree with this referee, and a similar comment from referee 3, that the way we depicted the results in the original panels C, F and B, E was redundant and confusing. Following the suggestion of referee 3 we have now merged panel C with F and B with E in Figures 2C and 2I, respectively. However, we believe that the high magnification images provide a better illustration of the CB_1 cellular misdistribution than low magnification ones. This, together with the graphs included in the figure showing the quantification of CB_1 associated fluorescence and misdistribution in at least 30 neurons per culture in three different cultures move us to kindly ask this referee to keep the original high magnification images. To address the query about CB_1 misdistribution *in vivo* we have quantified the degree of co-localization of CB_1 and the lysosomal marker LAMP1 in Purkinje cells of the cerebellum of WT and ASM-KO mice. In agreement with the *in vitro* data we observed increased localization (quantified by the Mander´s coefficient) of CB_1 in lysosomes in the ASM-KO compared to WT mice (Figure 2D).

4) Lysosomal accumulation of CB1 has been observed in ASM-KO or upon SM supplementation. The authors discuss that lysosomal degradation may contribute to the reduced levels of CB1 (discussion, page 12). Would inhibition of lysosomal degradation in cultured primary neurons (for example upon addition of SM) effect CB1 levels and its cellular distribution?
Following the suggestion of this referee, we have quantified CB_1 levels under conditions of lysosomal function inhibition. We have done so in WT neuronal cultures in which SM was added or not in the presence or absence of the lysosomal inhibitor Bafilomycin. Lysosomal inhibition prevented the SM-induced reduction of CB_1 levels by 61% (Figure 2J). This result supports the concept that lysosomal accumulation and degradation of CB_1 upon high SM levels contributes to CB_1 reduction.
Along these lines, can normal cellular distribution of CB1 be restored upon treatment of ASM-KO with SMase?
Yes, SMase treatment reduced the aberrantly high co-localization of CB_1 with lysosomes in ASM-KO cultured neurons (Figure 2N).

5) Western blot image of CB1 in Fig 2I does not support the reported 45% increase in CB1 protein levels.

We now show a more representative WB example of the reported 45% mean increase (Figure 2M). However, as we also indicate in the text, this result did not reach statistical significance (page 7).

6) The authors report a body weight gain in ASM-KO mice treated with PF and claim that no differences were observed in the WT. However, differences can also be observed upon PF application in the WT (Fig 4A). No statistics was included to support the rescue phenotype.
We have performed statistical analysis of the slopes of the weekly weight data. This analysis indicated a significant difference between the vehicle treated ASM-KO mice with respect to the other three groups (vehicle treated WT mice and PF treated WT and ASM-KO mice) (Figure 4A).

7) Histopathological analysis of the ASM-KO cohort treated longer with PF and included into the survival analysis (Fig 4F) has not been performed and could add additional, therapeutically relevant information when compared with the treatment efficacy over 8 weeks.
We apologize for not being able to perform the histopathological analysis this referee suggests since we did not collect tissue samples from the mice devoted to the survival analysis.

8) Figure 5 should be improved to have the analysis more consistent. AEA and NSM levels were monitored in the hippocampal extracts only (Fig 5A and B). SM analysis was done for the cerebellum, hippocampus and the cortex (hippocampus was indeed less affected). Further immunohistological analysis only revealed changes in the cerebellum. This makes it difficult for a reader to follow the logic. It would be helpful to have AEA, NSM, CB1 and SM levels plus histological examination for the same brain region (or at least the one affected most).
We apologize for the confusing presentation of the data in the original figure. Following the reviewer's suggestion we now present AEA, NSM, CB_1 and SM levels plus histological examination all from the cerebellum, which is the most affected area in the disease (Figure 5).
Showing only Lamp1 staining in Fig 5D is not beneficial. Cellular marker or at least dapi images should be included to judge what the authors aim to present. Similar to comment #3, also in Fig 5F and G it would be beneficial to have a larger overview to better judge the GFAP and Iba1 pathology.
9) Is the treatment with PF able to correct the cellular distribution phenotype of CB1?
We now show co-labelling of CB_1 with DAPI in the cerebellum (Figure 5E) and co-labelling of CB_1 with Lamp1 in Purkinje cells (Figure 5F). This has allowed us to quantify lysosomal area in these cells and to determine that PF treatment reduced the aberrant high co-localization of CB_1 with these organelles in the ASM-KO mice as indicated by changes in the Mander´s coefficient (Figure 5F).

10) Acute treatment is not offering any additional information beyond toxicity assessment. SM levels were only slightly reduced in the cortex. However, the neuroinflammatory effect was also seen in the hippocampus where no SM reduction could be detected. The authors should consider if the statement on page 11 "...and also revealed efficacy after a single administration of high PF doses." is fully supported by the data.
We have corrected this statement accordingly.

11) I appreciate the efforts towards examining CB1 signalling in other diseases where SM accumulates. However, if the authors wish to study CB1 signalling in NPC, more evidence should be provided, including Western blot analysis of CB1 across different brain regions and their corresponding SM levels. Moreover, brain material and cultured cells from more than 1 NPC patient should be included to make this result conclusive. Also in Fig 7 more consistency would be helpful for a reader. Fig 7A shows CB1 levels in the cerebellum of an NPC1-deficient mouse model, human tissue reveals CB1 staining of a hippocampus and cultured cells of an NPC patient treated with PF were only examined for the levels of SM (CB1 analysis is missing). Analysis of other phenotypes such as cellular distribution of CB1, lysosomal morphology or levels of cholesterol upon PF treatment would provide additional information to judge the therapeutic potential of CB1 modulation in NPC disease.
We thank the referee for this suggestion, which encouraged us to deepen our analysis of NPC. We have focused on the cerebellum, which is a most affected brain area in the disease. As for the ASM-KO mouse we did not find significant changes in CB_1 levels analyzed by Western blot in cerebellar extracts of NPC^{nmf164} compared to WT mice (Figure 7A). However, immunofluorescence analysis showed a significant 32% CB_1 protein reduction in the Purkinje cells (Figure 7B). As with the NPA patient it is very difficult to obtain brain tissue form NPC patients. Still, we believe the findings in the human scenario, while not conclusive, are worth showing. To be more consistent with the mouse data we have analyzed CB_1 levels in the Purkinje cells of the cerebellum of the control and NPC-affected children finding a significant reduction in the latter (Figure 7C). To satisfy this referee query we have analyzed several phenotypes in control and NPC cultured fibroblasts treated or not with PF. PF treatment in NPC fibroblasts reduced SM and cholesterol levels, and diminished the aberrantly high co-localization of CB_1 in lysosomes (Figures 7D, E, F). Besides extending the analysis in the cultured fibroblasts, we have performed an acute *in vivo* treatment with PF. A single oral administration of the high dose (5mg/kg) of PF reduced, after 48 hours, the levels of SM and cholesterol in the cerebellum of NPC^{nmf164} mice (Figure 7G) and diminished inflammation as indicated by the lower area of microglia in the PF treated mice (Figure 7H).

Minor issues

1) Authors should discuss differences between CB1 (neuronal) and CB2 (immune cells) expression patterns that may result in different signalling networks in neurons vs microglia. Potential common (or diverse) mechanism of FAAHi effect in different nervous system cells could be discussed.
This is now discussed on page 13.

2) Authors should consider improving figure legends such as in Fig 4A and F to distinguish more easily between different conditions.
The figure legends have been improved.

3) Consider re-phrasing "...dual impact" on page 11 of the discussion.
Dual impact has been rephrased to “multiple impacts”.

4) Reference Nr 50 on page 15 of the discussion should be corrected according to the common reference style.

We thank this reviewer for the careful revision of our paper. The style of these references has been corrected.

Referee #3

We thank this referee for the comments, for finding the study of interest, and for acknowledging the *in vitro* study in cultured neuronal cells as an optimal method to validate the role of CB_1 receptor and its direct link with the SM accumulation. We explain below how we have addressed his/her queries:

Major Comments:

-Figure 1 and Results: The fluorescence intensity measurement doesn't represent a quantitative analysis of CB1 protein. The authors should perform the quantitative analysis of CB1 by WB experiments in the same brain regions used for qPCR experiments.

Western blot analysis of CB_1 levels in the cerebellum is shown in the Figure 1D. For the sake of simplicity, and following the recommendation of referee 1, we have focused on this brain area in all panels of Figure 1, since it is the most severely affected in the disease. WB analysis of CB1 levels in the hippocampus and cortex is shown in Figure S1. The additional analysis of cell type expression of CB_1 by immunofluorescence, which uncovered the specific CB_1 reduction in neurons and not in glial cells (Figures 1E,F,G), may explain why we do not see differences in the levels of this receptor by Western blot of total extracts. This is discussed in the revised text.

-Results page 5: Since the CB1 receptors are well abundant in the prefrontal cortex, it should be interesting if the authors indicate in text and in the figure legend of Fig.1C which cortical regions have been analyzed and if they performed all the experiments in the same mouse cortical regions.

We performed all experiments in the prefrontal cortex. This is now indicated in the legend of Figure S1 and in page 6.

-Figure 1D: the author should improve the resolution of cerebellum images in which it is difficult to appreciate the signal of CB1 receptor.

We now provide with better resolution images of CB_1 co-labelled with different cellular markers such as Calbindin (for Purkinje cells), GFAP (for astrocytes) and F4/80 (for microglia) in the cerebellum of WT and ASM-KO mice (Figures 1E,F,G). We also show the quantification of CB_1 intensity and its degree of co-localization (Mander´s coefficient) with each cell type (Figures 1E,F,G).

-Figure 1E: In the IF staining on cerebellum, the MAP2 signal is almost undetectable in the slides of both CTR and NPA patients and it is also complicated to understand the morphology of this brain region. The author should improve the IF experiments in order to better analyze the decrease of CB1 signal in the cerebellum of NPA patients. They could also try to perform a labelling with Calbindin/CB1/TOPRO markers.

We have improved the MAP2 staining in the medium bulb of the control and NPA patients (Figure S2). Since MAP2 is not a good marker for neurons in the cerebellum we have followed this reviewer suggestion and performed triple labelling with CB_1, the specific Purkinje cell marker calbindin, and TOPRO (Figure 1H). Quantification of CB_1

reduction in the Purkinje cells of the NPA patient compared to the control child is added in the graphs of Figures 1H and S2.

Figure 2

-In vitro study:

Figure 2A: In order to quantify the expression levels of CB1 receptor, the authors should perform the WB analysis on WT and ASM-KO hippocampal cells. The immunofluorescence experiments represent only a semi-quantitative analysis able to support the WB data. Moreover, since the authors explain in the results that the main pathological hallmark of the ASMD is represented by the increase of SM levels, it is important to quantify the amount of sphingomyelin in both WT and ASM-KO neuronal cells.

We now provide WB analysis of CB_1 levels (Figure 2B) showing a 66% reduction in the ASM-KO compared to WT cultured hippocampal neurons. We have also measured the SM levels confirming a 78% increase in the ASM-KO neurons (Figure 2E).

-Figure 2C: Since the measure of CB1 fluorescent intensity has been already quantified in the figure 2B, the authors should replace the CB1 quantification graph in Fig 2C with another one in which they show the LAMP1 quantification as marker of autophagy impairment. Moreover, the authors showed an increase of CB1 in the cell body of ASM-KO cells. Since this increase is associated with an increase of SM levels, the author should show a quantification of SM levels among WT and ASM-KO cells.

We apologize for the redundancy in the original figure and thank this reviewer for the suggestion. We have now merged panels B and C in the Figure 2C in which we also include the colocalization of CB1 with Lamp1 in WT and ASM-KO cultured cells quantified by the Mander´s coefficient. We have also quantified the accumulation of Lamp1 as marker of autophagy impairment in the ASM-KO neurons. Although we do not include the data in the figure, since it confirms those published in our previous work (Gabande-Rodriguez et al., Cell Death Diff 2014), Lamp1-associated intensity and the area of lysosomes increased by 64% and 74%, respectively in the ASM-KO neurons. We now provide the quantification of SM levels showing that they are 78% higher in ASM-KO neurons compared to WT (Figure 2E). In addition we show that the increased co-localization of CB_1 and Lamp1 also occurs *in vivo* in the Purkinje cells of the cerebellum of ASM-KO compared to WT mice (Figure 2D).

-Figure 2D-2E: The authors could show the qPCR and the IF experiments in WT and also ASM-KO cell lines. Furthermore, they should perform the WB analysis for CB1 receptor with the relative protein quantifications in the following neuronal cell lines: WT, WT+40uM SM and ASM-KO.

We now show qPCR results and WB analysis of CB_1 in WT and ASM-KO neuronal cultures (Figures 2A and 2B) and in WT cultures treated or not with SM (Figures 2G and 2H).

-Figure 2F: Since the authors quantified in both panels 2E and 2F the CB1 signal using the same cell lines, they should replace the figure 2E with figure 2F.

As with the original panels B and C we apologize for the redundancy and have merged the original panels E and C in the Figure 2I in which we quantified not only the levels of CB_1 but also the Mander´s coefficient of CB_1 and Lamp1 co-localization in WT neuronal cultures treated or not with SM.

-Figure 2G, 2H, 2I: The authors should also perform the experiments in the WT cell lines as positive control.
These experiments are now shown in Figure S3 and mentioned in page 8.

-Figure 3A-3E: the authors should also perform the experiments in the WT neuronal cell line as CTR cell line.
These experiments are now shown in Figure S5 and mentioned in page 9.

-Page 7: The authors demonstrated that FAAH inhibitors act with the participation of CB1 receptors by an indirect experiment in which they measured the SM levels in ASM-KO cells treated with PF-04457845 and with SR+PF-04457845. The authors should also evaluate the expression levels of the CB1 receptor by WB experiments on ASM-KO cells treated with PF-04457845 and SR+PF-04457845.
Figure 3C: The authors should directly quantify of CB1 receptor levels by WB experiments in WT and ASM-KO cells treated with AEA with or without the GW4869
Figure 3F: In order to both evaluate the neuronal morphology and CB1 neuronal distribution, the authors should perform a Co-IF experiments with MAP2 and CB1 receptor markers on ASM-KO treated cells and WT cells. The authors should also quantify the levels of CB1 in ASM-KO cells by WB analysis upon the treatment with FAAH inhibitors.

The evaluation of expression levels of CB_1 receptor by WB in ASM-KO neurons treated with AEA with or without GW4869, with the different FAAH inhibitors, or with PF and SR-PF are now shown in the Figure S4 and mentioned in pages 8-9.

Figure 5B: The authors quantified the NSM protein in hippocampal extracts from WT and KO mice. They should provide an image of the entire WB filter with all experimental groups of mice. Moreover, in order to quantify the levels of CB1 receptor they should perform a WB analysis of this protein in hippocampal extracts of treated mice. Page 9: the authors performed the molecular and biochemical analyses on hippocampal, cerebellum and cortex extracts of ASM-KO treated mice. They could provide immunofluorescence experiments for LAMP1, IBA1 and GFAP markers also in the hippocampus of KO treated mice.
Figure 5D, 5E: The authors show in the panel D, the LAMP1 staining in cerebellum samples of treated mice. Since it is difficult to appreciate to LAMP1 localization and the cerebellum morphology, the authors should perform a co-labelling with LAMP1 markers and DAPI. Moreover, since the authors demonstrated an increase in the LAMP1 and CB1 colocalization in ASM-KO cells, they should also perform a co-labelling experiment with LAMP1 and CB1 markers in cerebellum and hippocampus of treated mice. This data should support the biochemical experiments performed in the same animals.
Figure 5F-5G: the authors show the IBAI and GFAP staining in the cerebellum of treated mice. It is difficult to appreciate the cerebellum morphology, probably because they used a different magnification respect to the previous images. In order to better understand the signal of both markers, the author should provide images with higher resolution and lower magnification. Moreover, they could provide the inflammation analysis (IBA1 and GFAP markers) in the hippocampus of treated mice.

Following the reviewer recommendations, and to avoid the confusing mixture of results in different brain areas, we have focused our quantifications in the cerebellum that is the most affected region in the disease. Thus, we have analyzed in this brain area all the parameters requested for this referee in the hippocampus. NSM levels have been analysed in the cerebellum by WB in the Figure 5C. We also provide with intensity analysis of CB_1 in the cerebellum (Figure 5E) and with co-localization analysis of Lamp1 and CB_1 in Purkinje cells of WT and ASM-KO quantified by Mander´s coefficient (Figure 5F). GFAP and Iba1 immunofluorescence analysis in the cerebellum remain as in the original figure (Figures 5H and 5I).

Figure 6A: the authors should perform a tunnel assay in a representative brain region of KO mice treated with higher doses of PF in order to exclude any toxic effect on the neuronal cells.

To determine cell toxicity we have used cleaved caspase3 staining as a tunnel assay to detect apoptotic cells, in the cerebellum of WT and ASM-KO mice treated with the different PF doses. These results confirm the lack of significant toxicity of PF treatments, with the highest dose showing great variability, and are shown in the Figure S7 and mentioned in page 12.

Figure 6C: the authors performed the IF experiments with IBA marker in order to evaluate the microglia activation in the brain regions of KO mice treated with different concentration of PF. In order to better appreciate the morphology of brain regions and IBA distribution, the authors should perform immunofluorescence experiments in the mouse brain regions with IBA1 marker and DAPI.

DAPI staining made less clear the Iba1 labelling, which is the focus on Figure 6C. Therefore, we kindly ask this referee to keep the images as Iba1 single staining.

Page 11: To understand the role of CB1 in the neuropathology development, the authors should improve the in vitro studies in NPC fibroblasts by:

-Quantifying the CB1 receptor in WT and NPC fibroblast cell line from patients with WB experiments. Treating the NPC and WT fibroblast cell line with SMase and evaluating the CB1 levels upon the treatment. Moreover, in order to quantify the levels of CB1 receptors they should perform WB experiments in the brain samples and also Immunofluorescence staining for CB1 receptor in the hippocampus of WT and NPC1 nmf164 mice.

Detection of CB_1 by WB is not straightforward. We were able to set specific conditions to do so in the mouse samples but none of the antibodies tested worked to detect CB_1 in the human fibroblasts. As in the ASM-KO mice, quantification of CB_1 levels by WB of total cerebellar extracts from $NPC1^{nmf164}$ mice did not show significant differences compared to age-matched WT mice (Figure 7A), likely because the CB_1 reduction is specific to neurons. However, by immunofluorescence we observed a significant 32% reduction of CB_1 levels in the Purkinje cells of the cerebellum of $NPC1^{nmf164}$ mice (Figure 7B) (page 13).

-Performing IF analysis for CB1 and LAMP1 markers in order to evaluate the CB1 co-localization with lysosomes and a possible block of autophagy in NPC fibroblast cell line.

We performed co-labelling of CB_1 and Lamp1 in control and NPC fibroblasts. The quantification of the Mander´s coefficient indicated an increased co-localization of CB_1 and Lamp1 in the NPC compared to the control fibroblasts that was diminished by PF treatment (Figure 7F) (page 13).

-To test the therapeutic effectiveness of the PF treatment in NPC1 nmf164 mouse models, the authors could perform a short term study with the best dosage of PF and analyze the CB1 and SM levels in the brain samples of treated mice. Following this referee's suggestion we conducted a short-term study by administering a single dose of PF (5mg/kg) to $NPC1^{nmf164}$ mice. After 48 hours we observed a reduction in SM and cholesterol levels in the cerebellum of the PF-treated mice compared to the vehicle-treated (Figure 7G). Moreover, the acute PF treatment diminished the inflammation as indicated by the reduction in the area of microglia (Figure 7H) (page 13).

Minor comments:

FigureS1A: the authors should explain how they calculated the fluorescence intensity in the hippocampus region of treated mice and if they normalized the data respect to the brain areas or to the number of cells analyzed.
We now clarify this issue in the methods. In the original studies fluorescence intensity was normalized to the brain area. In the new immunofluorescence studies performed in specific cell types fluorescence intensity was calculated per cell area.

-Page 6: In the results it has been described a change in CB1 distribution in the ASM-KO neuronal mouse cell line. The authors should comment this part in the results and in the discussion.
These results are commented in page 7 and discussed in page 14.

Figure 6: the authors should indicate which cortex regions they have analyzed. The prefrontal cortex was analyzed. This is now indicated in the legend of the Figure S1 and in page 6.

Figure legend 7A: In the figure 7A and in its figure legend there is a discordance about the nuclei marker used in the experiment. The authors should indicate the nuclei marker.
We apologize for the discrepancy that has now been corrected

Figure 7A, 7B: The authors should show immunofluorescence images at low magnification in order to better appreciate the CB1 reduction and distribution in hippocampus NPC1 nmf164 mice.
Following the referee's suggestion, and to avoid mixed data from different brain areas, we have now focused the study on the cerebellum, which is the most affected brain area in the disease. CB_1 levels and distribution have been analyzed by immunofluorescence in the Purkinje cells of $NPC1^{nmf164}$ mice (Figure 7B) and of the NPC patient (Figure 7C).

1st Aug 2020

Dear Dr. Ledesma,

Thank you for the submission of your manuscript to EMBO Molecular Medicine. We have now heard back from the two referees whom we asked to re-evaluate your manuscript.

You will see that while the 2nd referee is now satisfied, the 1st one still isn't and we decided to give you another chance to address the following issues:
-statistical significance must be provided (exact n and p-values, not a range, along with the statistical test used)
-CB1 misdistribution in vivo & LAMP1 staining
-immunohistochemistry data in human specimen
-and finally, we would like you to also try at least to confirm the IFA data with biochemical experiment using positive and negative controls that would eventually attest to the population heterogeneity/cell specificity should this attempt be unsuccessful.

Please revise your article as requested and provide a point-by-point letter. According to the mature of the revision, we may reserve the right to ask the referee to evaluate the new data.

I look forward to seeing a revised form of your manuscript as soon as possible.

Yours sincerely,

Celine Carret

Celine Carret, PhD
Senior Editor
EMBO Molecular Medicine

***** Reviewer's comments *****

Referee #1 (Remarks for Author):

I appreciate the efforts that authors put into performing additional experiments that were requested and improving the manuscript quality. However, few concerns regarding this study still

remain. The major remaining concern is that immunofluorescent data could to a larger extent not be validated by other means such as biochemical experiments. The authors argue with cell specificity of their effects, but taking into account that neuronal population is not a minor population in brain extracts, one would have expected effects to be detectable by biochemical analysis as well. Apart from this issue, statistical significance is missing in several experiments, questioning the robustness of the data. Furthermore, CB1 misdistribution in vivo relies on LAMP1 staining that shows an unusual pattern in the ASM KO that differs between the presented figures (Fig 2D and Fig 5F), making data interpretation difficult. Is the reduction in Fig 7B specific for CB1 (would calbindin staining of the same image still reveal preserved Purkinje neurons, was the Purkinje cell marker included into this analysis)?. In addition, immunohistochemistry data in human specimens still remain hard to interpret, beside the fact that only 1 patient has been analyzed.

Referee #3 (Comments on Novelty/Model System for Author):

The revised version of the manuscript has clearly showed the clinical potential of a new therapeutic approach for the treatment of severe neurological pathologies like the acid sphingomyelinase deficiency (ASMD).
The in vitro and in vivo models used are adequate for studying and validating the efficacy of the treatment for ASMD and other sphingolipidoses.
Moreover, the new data shown have increased the quality and medical impact of the article.

Referee #3 (Remarks for Author):

The revised version of the manuscript from Bartoll et al., entitled: "Inhibition of Fatty Acid Amide Hydrolase Prevents Pathology in Neurovisceral Acid Sphingomyelinase Deficiency by Rescuing Defective Endocannabinoid Signaling" has been significantly improved.
The authors accurately have addressed all the requests and suggestions previously indicated.
In particular, they thoroughly performed the new in-vivo and in-vitro experiments providing an in-depth description of the showed data.
In addition, it is worth noting that the authors notably ameliorated the quality of images and improved the discussion.

As a conclusion, this new version of manuscript is suitable for the publication in EMBO Molecular Medicine.

POINT-BY-POINT LETTER, EMM-2019-11776-V2
12th August 2020

Below I detail the experiments and modifications done, which are highlighted in the revised text and in the modified Figure 2D and the new Appendix Supplementary Figure S2.

1.The major remaining concern is that immunofluorescent data could to a larger extent not be validated by other means such as biochemical experiments. The authors argue with cell specificity of their effects, but taking into account that neuronal population is not a minor population in brain extracts, one would have expected effects to be detectable by biochemical analysis as well.
To attest cell population heterogeneity and imbalance in the ASM-KO compared to WT mice we now provide with the quantification of astrocytes and microglia by immunofluorescence and also by Western blot using specific cell markers in cerebellar extracts (New Supplementary Figure S2). These data confirm that the population of astrocytes and microglia, where we do not find reduced CB1, increase by 6-fold and 4-fold, respectively (as indicated by immunofluorescence). In contrast neuronal population diminishes (Figure 5F). These results strongly support that the presence of remarkable astrogliosis and microgliosis, could prevent detecting the neuronal specific reduction of CB1 in the biochemical experiments (now mentioned in page 6).

2. *Statistical significance is missing in several experiments, questioning the robustness of the data.*
Exact n and p-values along with the statistical test used is now provided in the figure legends for all significant values. Due to the Covid19 outbreak during the time of revision of this manuscript we were obliged to drastically reduce mouse colonies and cell culture work. This, together with the low fertility of NPC mice and the difficulty to grow enough cells from the human NPC fibroblast line, prevented the analysis of a larger sample size. This may explain why in several experiments in Figure 7 the clear trend we observe in the data does not reach statistical significance.

3. CB1 misdistribution in vivo relies on LAMP1 staining that shows an unusual pattern in the ASM KO that differs between the presented figures (Fig 2D and Fig 5F), making data interpretation difficult.
We realized that the brightness in the images shown in figure 2D was higher than in those in Figure 5F. This might have been confusing for the referee. We now show images with the same brightness settings that evidence the similarities in the patterns in both figures. Indeed, Lamp1 staining show an unusual pattern in the ASM-KO brains compared to WT since lysosomes are enlarged due to lipid

accumulation. For quantification the same threshold was set for Lamp1 staining in WT and ASM-KO samples.

4. Is the reduction in Fig 7B specific for CB1 (would calbindin staining of the same image still reveal preserved Purkinje neurons, was the Purkinje cell marker included into this analysis)?

We now add in Fig 7B images the staining for Calbindin, which was indeed included for the analysis, showing preserved Purkinje neurons in which CB1 reduction is specific.

5. Immunohistochemistry data in human specimens still remain hard to interpret, beside the fact that only 1 patient has been analyzed.

Following this referee suggestion we stressed in the text that the results obtained in the human specimens are not conclusive since they belong to just one patient. However, the reductions observed in Purkinje cells identified with the Calbindin marker were very clear: 58% in the ASMD patient and 37% in the NPC patient. We believe this information is worth showing and hope to confirm it in the future as soon as more human specimens of these rare and ultra-rare diseases will be available.

20th Aug 2020

Dear Dr. Ledesma,

Thank you for the submission of your revised manuscript to EMBO Molecular Medicine. We have now received the enclosed report from the referee who was asked to re-assess it. As you will see the reviewer is now supportive and I am pleased to inform you that we will be able to accept your manuscript pending final editorial amendments.

Please submit your revised manuscript within three weeks. I look forward to seeing a revised form of your manuscript as soon as possible.

Yours sincerely,

Celine Carret

Celine Carret, PhD
Senior Editor
EMBO Molecular Medicine

***** Reviewer's comments *****

Referee #1 (Remarks for Author):

The authors have addressed my remaining concerns adequately.

EMBO PRESS

YOU MUST COMPLETE ALL CELLS WITH A PINK BACKGROUND

PLEASE NOTE THAT THIS CHECKLIST WILL BE PUBLISHED ALONGSIDE YOUR PAPER

Corresponding Author Name: MARIA DOLORES LEDESMA
Journal Submitted to: EMBO MOLECULAR MEDICINE
Manuscript Number: EMM-2019-11776

USEFUL LINKS FOR COMPLETING THIS FORM

http://www.antibodypedia.com
http://1degreebio.org
http://www.equator-network.org/reporting-guidelines/improving-bioscience-research-repor
http://grants.nih.gov/grants/olaw/olaw.htm
http://www.mrc.ac.uk/Ourresearch/Ethicsresearchguidance/Useofanimals/index.htm
http://ClinicalTrials.gov
http://www.consort-statement.org
http://www.consort-statement.org/checklists/view/32-consort/66-title
http://www.equator-network.org/reporting-guidelines/reporting-recommendations-for-tumo
http://datadryad.org
http://figshare.com
http://www.ncbi.nlm.nih.gov/gap
http://www.ebi.ac.uk/ega
http://biomodels.net/
http://biomodels.net/miriam/
http://jjj.biochem.sun.ac.za
http://oba.od.nih.gov/biosecurity/biosecurity_documents.html
http://www.selectagents.gov/

Reporting Checklist For Life Sciences Articles (Rev. June 2017)

This checklist is used to ensure good reporting standards and to improve the reproducibility of published results. These guidelines are consistent with the Principles and Guidelines for Reporting Preclinical Research issued by the NIH in 2014. Please follow the journal's authorship guidelines in preparing your manuscript.

A- Figures
1. Data
The data shown in figures should satisfy the following conditions:

- the data were obtained and processed according to the field's best practice and are presented to reflect the results of the experiments in an accurate and unbiased manner.
- figure panels include only data points, measurements or observations that can be compared to each other in a scientifically meaningful way.
- graphs include clearly labeled error bars for independent experiments and sample sizes. Unless justified, error bars should not be shown for technical replicates.
- if n< 5, the individual data points from each experiment should be plotted and any statistical test employed should be justified
- Source Data should be included to report the data underlying graphs. Please follow the guidelines set out in the author ship guidelines on Data Presentation.

2. Captions

Each figure caption should contain the following information, for each panel where they are relevant:

- a specification of the experimental system investigated (eg cell line, species name).
- the assay(s) and method(s) used to carry out the reported observations and measurements
- an explicit mention of the biological and chemical entity(ies) that are being measured.
- an explicit mention of the biological and chemical entity(ies) that are altered/varied/perturbed in a controlled manner.

- the exact sample size (n) for each experimental group/condition, given as a number, not a range;
- a description of the sample collection allowing the reader to understand whether the samples represent technical or biological replicates (including how many animals, litters, cultures, etc.).
- a statement of how many times the experiment shown was independently replicated in the laboratory.
- definitions of statistical methods and measures:
  - common tests, such as t-test (please specify whether paired vs. unpaired), simple χ2 tests, Wilcoxon and Mann-Whitney tests, can be unambiguously identified by name only, but more complex techniques should be described in the methods section;
  - are tests one-sided or two-sided?
  - are there adjustments for multiple comparisons?
  - exact statistical test results, e.g., P values = x but not P values < x;
  - definition of 'center values' as median or average;
  - definition of error bars as s.d. or s.e.m.

Any descriptions too long for the figure legend should be included in the methods section and/or with the source data.

In the pink boxes below, please ensure that the answers to the following questions are reported in the manuscript itself. Every question should be answered. If the question is not relevant to your research, please write NA (non applicable). We encourage you to include a specific subsection in the methods section for statistics, reagents, animal models and human subjects.

B- Statistics and general methods

Please fill out these boxes (Do not worry if you cannot see all your text once you press return)

1.a. How was the sample size chosen to ensure adequate power to detect a pre-specified effect size?	The number of mice used was selected on the basis of previous phenotyping analyses conducted in the same models and calculating the statistical power of the experiment. (page 17)
1.b. For animal studies, include a statement about sample size estimate even if no statistical methods were used.	PAGThe sample size for each experiment is included in the figure legends. (page 17)
2. Describe inclusion/exclusion criteria if samples or animals were excluded from the analysis. Were the criteria pre-established?	No samples or animals were excluded from the analysis (page 17)
3. Were any steps taken to minimize the effects of subjective bias when allocating animals/samples to treatment (e.g. randomization procedure)? If yes, please describe.	Mice were genotyped and according to the genotype randomly assigned to the experimental groups. No outliers were excluded in the study. (page 17)
For animal studies, include a statement about randomization even if no randomization was used.	Mice were genotyped and according to the genotype randomly assigned to the experimental groups. (Page 17)
4.a. Were any steps taken to minimize the effects of subjective bias during group allocation or/and when assessing results (e.g. blinding of the investigator)? If yes please describe.	Investigators assessing and measuring results were blinded to the intervention. (page 18)
4.b. For animal studies, include a statement about blinding even if no blinding was done	Investigators performing the experiments were blinded to the mouse genotype. (page 18)
5. For every figure, are statistical tests justified as appropriate?	Yes, see next paragraph
Do the data meet the assumptions of the tests (e.g., normal distribution)? Describe any methods used to assess it.	Normality of the data was tested using the Shapiro–Wilk test. For two-group comparisons, the Mann–Whitney U-test for non-parametric data or a two-sample Student's t-test for data with parametric distribution was used. For multiple comparisons, data with a normal distribution were analyzed by one-way ANOVA followed by Bonferroni, Tukey or Games–Howell post hoc test. The statistical significance of non-parametric data was determined by the Kruskal–Wallis test to analyze all experimental groups. The Mann–Whitney U-test was used to analyze paired genotypes, applying the Bonferroni correction. Linear Regression test with 95% confidence interval was used to analyze slope differences (page 23)
Is there an estimate of variation within each group of data?	There is an implicit variation in the experimental groups (e.g. gender, age, conditions) that has been addressed by normality tests (as described below) to perform parametric or non-parametric analyzes (page 23)

Is the variance similar between the groups that are being statistically compared?	The assumption of homogeneity of variances was tested using the Shapiro–Wilk test. Homogeneity of variances was required to perform parametric tests (t-test and ANOVA) and when there was no homogeneity of variances, non-parametric tests were performed (Kruskal – Wallis test) (page 23)

C- Reagents

6. To show that antibodies were profiled for use in the system under study (assay and species), provide a citation, catalog number and/or clone number, supplementary information or reference to an antibody validation profile. e.g., Antibodypedia (see link list at top right), 1DegreeBio (see link list at top right).	Antibodies against the following proteins were used in western blots and for immunofluorescence analysis: Calbindin (Mouse, Swant, 300, dilution 1:500), CB1 (Rabbit, Frontier Institute, AF380, 1:500), CB2 (Mouse, R&D Systems, 352110, 1:500), F4/80 (Rat, Abcam, ab6640, 1:500), GAPDH (Mouse, Abcam, ab8245, 1:5000), GFAP (Mouse, Millipore, MAB3402, 1:1000), Iba1 (Rabbit, Wako, 019-19741, 1:500), Lamp1 (Rat, DSHB, 1D4B, 1:500), MAP2 (Chicken, Biolegend, 822501, 1:500), NSM (Rat, Santa Cruz, sc-166637, 1:200) and PSD-95 (Mouse, BD Transduction Laboratories, 610495, 1:500), cleaved caspase 3 (Asp 175) (Rabbit polyclonal, Cell Signaling, 9661, 1:200). page 18
7. Identify the source of cell lines and report if they were recently authenticated (e.g., by STR profiling) and tested for mycoplasma contamination.	NA

* for all hyperlinks, please see the table at the top right of the document

D- Animal Models

8. Report species, strain, gender, age of animals and genetic modification status where applicable. Please detail housing and husbandry conditions and the source of animals.	Breeding colonies were established from ASM heterozygous C57BL/6 mice (Horinouchi, Erlich et al., 1995) kindly donated by Prof. E.H. Schuchman (Mount Sinai School of Medicine, New York, NY, USA) and from C57BL/6J NPC1nmf164 mice carrying a D1005G mutation in Npc1 (Maue et al., 2012) purchased from Jackson laboratories. Animals were grouped by genotype and gender. The mice were kept in a 12-h light/dark cycle in a SPF (specific pathogen free) room. Male/female ASM-KO, Npc1nmf164 and WT littermates were analyzed between 1.5 and 9 months of age. (page 19)
9. For experiments involving live vertebrates, include a statement of compliance with ethical regulations and identify the committee(s) approving the experiments.	Procedures followed European Union guidelines and were approved by the CBMSO and Comunidad de Madrid Animal Welfare Committees (PROEX 175/17). (page 19)
10. We recommend consulting the ARRIVE guidelines (see link list at top right) (PLoS Biol. 8(6), e1000412, 2010) to ensure that other relevant aspects of animal studies are adequately reported. See author guidelines, under 'Reporting Guidelines'. See also: NIH (see link list at top right) and MRC (see link list at top right) recommendations. Please confirm compliance.	Compliance confirmed in page 19

E- Human Subjects

11. Identify the committee(s) approving the study protocol.	NA
12. Include a statement confirming that informed consent was obtained from all subjects and that the experiments conformed to the principles set out in the WMA Declaration of Helsinki and the Department of Health and Human Services Belmont Report.	Included in page 19
13. For publication of patient photos, include a statement confirming that consent to publish was obtained.	NA
14. Report any restrictions on the availability (and/or on the use) of human data or samples.	NA
15. Report the clinical trial registration number (at ClinicalTrials.gov or equivalent), where applicable.	NA
16. For phase II and III randomized controlled trials, please refer to the CONSORT flow diagram (see link list at top right) and submit the CONSORT checklist (see link list at top right) with your submission. See author guidelines, under 'Reporting Guidelines'. Please confirm you have submitted this list.	NA
17. For tumor marker prognostic studies, we recommend that you follow the REMARK reporting guidelines (see link list at top right). See author guidelines, under 'Reporting Guidelines'. Please confirm you have followed these guidelines.	NA

F- Data Accessibility

18: Provide a "Data Availability" section at the end of the Materials & Methods, listing the accession codes for data generated in this study and deposited in a public database (e.g. RNA-Seq data: Gene Expression Omnibus GSE39462, Proteomics data: PRIDE PXD000208 etc.) Please refer to our author guidelines for 'Data Deposition'.<br><br>Data deposition in a public repository is mandatory for:<br>a. Protein, DNA and RNA sequences<br>b. Macromolecular structures<br>c. Crystallographic data for small molecules<br>d. Functional genomics data<br>e. Proteomics and molecular interactions	NA
19. Deposition is strongly recommended for any datasets that are central and integral to the study; please consider the journal's data policy. If no structured public repository exists for a given data type, we encourage the provision of datasets in the manuscript as a Supplementary Document (see author guidelines under 'Expanded View' or in unstructured repositories such as Dryad (see link list at top right) or Figshare (see link list at top right).	NA
20. Access to human clinical and genomic datasets should be provided with as few restrictions as possible while respecting ethical obligations to the patients and relevant medical and legal issues. If practically possible and compatible with the individual consent agreement used in the study, such data should be deposited in one of the major public access-controlled repositories such as dbGAP (see link list at top right) or EGA (see link list at top right).	NA
21. Computational models that are central and integral to a study should be shared without restrictions and provided in a machine-readable form. The relevant accession numbers or links should be provided. When possible, standardized format (SBML, CellML) should be used instead of scripts (e.g. MATLAB). Authors are strongly encouraged to follow the MIRIAM guidelines (see link list at top right) and deposit their model in a public database such as Biomodels (see link list at top right) or JWS Online (see link list at top right). If computer source code is provided with the paper, it should be deposited in a public repository or included in supplementary information.	NA

G- Dual use research of concern

22. Could your study fall under dual use research restrictions? Please check biosecurity documents (see link list at top right) and list of select agents and toxins (APHIS/CDC) (see link list at top right). According to our biosecurity guidelines, provide a statement only if it could.	NA
